# High-resolution shape models of Phobos and Deimos from stereophotoclinometry

**DOI:** 10.1186/s40623-023-01814-7

**Published:** 2023-06-25

**Authors:** Carolyn M. Ernst, R. Terik Daly, Robert W. Gaskell, Olivier S. Barnouin, Hari Nair, Benjamin A. Hyatt, Manar M. Al Asad, Kielan K. W. Hoch

**Affiliations:** 1 Johns Hopkins University Applied Physics Laboratory, Laurel, MD 20723 USA; 2 Planetary Science Institute, Tucson, AZ 85719 USA; 3 Northwestern University, Evanston, IL 60208 USA; 4 University of Maryland, Baltimore County, Baltimore, MD 21250 USA; 5 University of British Columbia, Vancouver, BC Canada USA; 6 Brown University, Providence, RI 02912 USA; 7 University of California, La Jolla, San Diego, CA 92093 USA; 8 Space Telescope Science Institute, Baltimore, MD 21218 USA

**Keywords:** Phobos, Deimos, Martian moons, Small bodies, Shape, Topography, Stereophotoclinometry, Martian Moons eXploration

## Abstract

We created high-resolution shape models of Phobos and Deimos using stereophotoclinometry and united images from Viking Orbiter, Phobos 2, Mars Global Surveyor, Mars Express, and Mars Reconnaissance Orbiter into a single coregistered collection. The best-fit ellipsoid to the Phobos model has radii of (12.95 ± 0.04) km × (11.30 ± 0.04) km × (9.16 ± 0.03) km, with an average radius of (11.08 ± 0.04) km. The best-fit ellipsoid to the Deimos model has radii of (8.04 ± 0.08) km × (5.89 ± 0.06) km × (5.11 ± 0.05) km with an average radius of (6.27 ± 0.07) km. The new shape models offer substantial improvements in resolution over existing shape models, while remaining globally consistent with them. The Phobos model resolves grooves, craters, and other surface features ~ 100 m in size across the entire surface. The Deimos model is the first to resolve geological surface features. These models, associated data products, and a searchable, coregistered collection of images across six spacecraft are publicly available in the Small Body Mapping Tool, and will be archived with the NASA Planetary Data System. These products enable an array of future studies to advance the understanding of Phobos and Deimos, facilitate coregistration of other past and future datasets, and set the stage for planning and operating future missions to the moons, including the upcoming Martian Moons eXploration (MMX) mission.

**Graphical Abstract:**

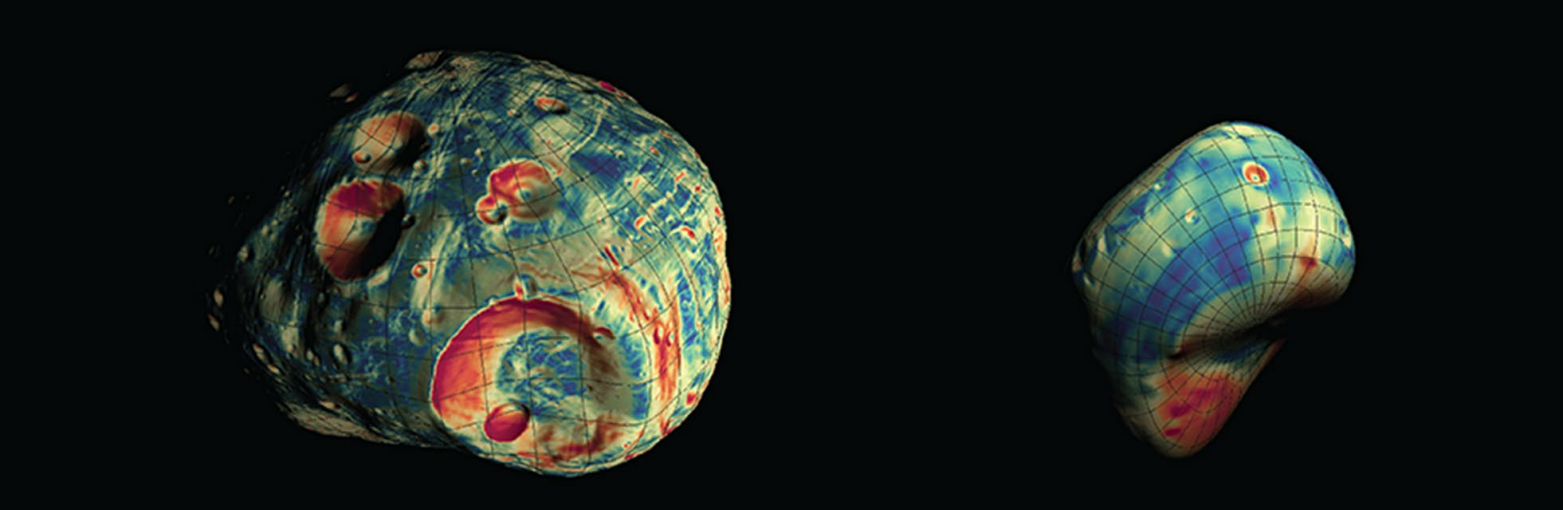

**Supplementary Information:**

The online version contains supplementary material available at 10.1186/s40623-023-01814-7.

## Main text

### Introduction

The flotilla of Mars-bound spacecraft sent by NASA, ESA, and the Soviet space program over the past ~ 45 years has provided many opportunities for disk-resolved observations of Phobos and Deimos, placing the martian moons among the best-observed small bodies in the solar system. Despite this status, the origin, evolution, composition, and structure of the moons are not well understood. Among the many scientific questions that remain unanswered are: How have geologic processes altered their surfaces? Do the moons’ surfaces show any evidence of their internal composition and structure? How did the formation of Stickney crater affect the current state of Phobos’ surface? How do Phobos and Deimos relate to one another?

This lack of understanding is in part due to major challenges inherent in analyzing the available datasets. The data obtained during a number of brief encounters by various spacecraft are difficult to synthesize and compare, due to complexities of coordinating spacecraft positioning, instrument pointing, data calibration, and data archives. Yet this synthesis is critical for comprehensive and up-to-date analyses of Phobos and Deimos. The combined datasets may harbor insights unobtainable by using each flyby or mission dataset in isolation.

The irregular shapes of the bodies also pose considerable obstacles that can only be overcome with improved shape models of Phobos and Deimos. Visualizing and mapping features on irregularly shaped bodies becomes a difficult task, and two-dimensional map projections can severely distort spatial relationships and size. Downslope direction is often not apparent, yet is crucial for interpreting surface geology, material mobilization, and landing site safety. Color and photometric studies can be tricky; in order to coregister and photometrically correct the data, a shape model is necessary to derive the proper image location and incidence, phase, and emission angles. Addressing these obstacles requires global and local knowledge of the shape, topography, slopes, and surface features of Phobos and Deimos.

We created high-resolution shape models of Phobos and Deimos using stereophotoclinometry (SPC) (Gaskell et al. 2008b; 2023) and united, for the first time, images from six separate spacecraft into a single coregistered collection. Here, we present these products, including details of the images and shape model properties, evaluations of model quality, comparisons to previous models, and derived geophysical maps (slope, gravitational acceleration, etc.). These foundational products enable future studies that will advance the frontiers of understanding Phobos and Deimos, facilitate coregistration of other past and future datasets, and set the stage for planning and operating future missions to the moons, including the upcoming Martian Moons eXploration (MMX) mission, led by the Japan Aerospace Exploration Agency (JAXA).

### Stereophotoclinometry method

We constructed shape models using stereophotoclinometry (SPC) methods, which are detailed in Gaskell et al. (2008b; 2023). The construction of shape and topography models using SPC began in the late 1980s (Gaskell 1988) in order to accurately locate landmarks on the surfaces of celestial bodies for the purpose of optical navigation. Since that time, SPC has been used to construct shape models for many irregular bodies, including asteroids (e.g., Gaskell et al. 2008a; Gaskell 2008, 2013a; Sierks et al. 2011; Jorda et al. 2012; Barnouin et al. 2019; Watanabe et al. 2019), comets (e.g., Jorda et al. 2016; Ernst et al. 2019), satellites (e.g., Gaskell et al. 2011; Gaskell 2011; Gaskell 2013b, Gaskell 2013c, 2013d, and 2013e; Daly et al. 2018; Weirich et al. 2019), dwarf planets (e.g., Park et al. 2019), and planets (e.g., Perry et al. 2015).

Here, we summarize how we used SPC to coregister images and create these new shape models. Existing archived shape models [Phobos: (Gaskell 2011); Deimos: (Thomas 1993; Thomas et al. 2000)] served as starting reference shapes for the new models. For Phobos, using a different starting reference shape (e.g., Willner et al. 2014) would not have meaningfully changed the solution; these bulk shapes are in general agreement with one another, we did not include the starting shape as a weight in the topographic solution, and there is global stereo coverage within the images available. For Deimos, the Thomas (1993) model was used as a starting reference shape because it was the best available model.

**Fig. 1:**
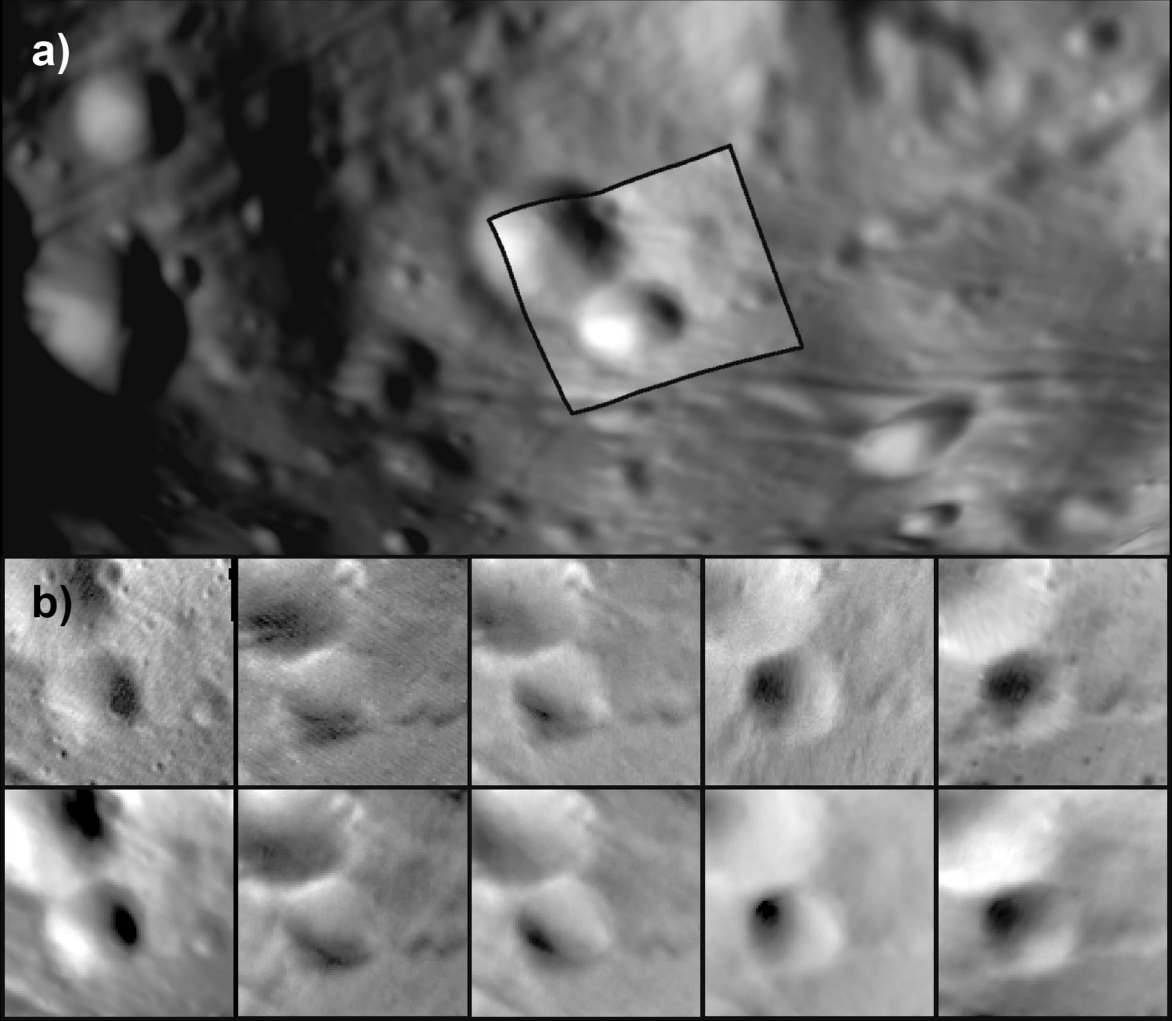
**a** A modeled maplet superposed on the global shape of Phobos. **b** Image (top row) and rendered model (bottom row) of the maplet shown in images from multiple spacecraft under different viewing geometries and illuminations

First, images were registered to these reference shapes. Second, the reference shape models were tiled with an initial set of maplets. (Maplets are local digital terrain models (DTMs), typically 99 × 99 pixels, centered around landmarks on the surface.) Maplet ground sample distance (GSD) is defined by the user. Third, the topography and albedo of each maplet are modeled using a combination of geometric stereo (to define the location of a maplet center) and photoclinometry (to determine surface tilts and estimate relative albedo) to match images obtained under a range of resolutions, incidence angles, and emission angles (Fig. 1). Gaskell et al. (2008b; 2023), Barnouin et al. (2020), and Palmer et al. (2022) elaborate on these processes. Fourth, additional sets of maplets are added to the model with increasingly fine GSD. The SPC method excels at uniting data of varying resolutions. New images are continually added to the model as they become available, and the DTMs are updated to incorporate the new information. As higher-resolution images become available, the model is tiled (either regionally or globally) with higher-resolution maplets as needed. The image geometry and resolution ranges allowed in a maplet depend on the variety and quality of images available. Ideally, images are excluded from maplets if they have emission angles > 60º, only cover an edge of the maplet, or are more than 4 times lower pixel scale than the GSD of the maplet (Palmer et al. 2022). (Some exceptions to these ideal constraints were made when constructing the Phobos and Deimos shape models, as detailed in later sections.)

Once many maplets are located in many images across a body, a linear minimization of the sum-squared residuals between the observed (portion of an image that covers the maplet) and modeled maplet yields a solution in a body-fixed frame for the position of each maplet center (center of the central pixel), the spacecraft attitude, and the location and rotation of the body (see Palmer et al. 2022 for more details on this process). Additional information is employed to improve this solution, such as the position of maplets on the limbs of some images, correlations of overlapping maplets due to common topography, and nominal spacecraft trajectory information (Gaskell et al. 2008b; 2023; Barnouin et al. 2020; Palmer et al. 2022).

**Fig. 2:**
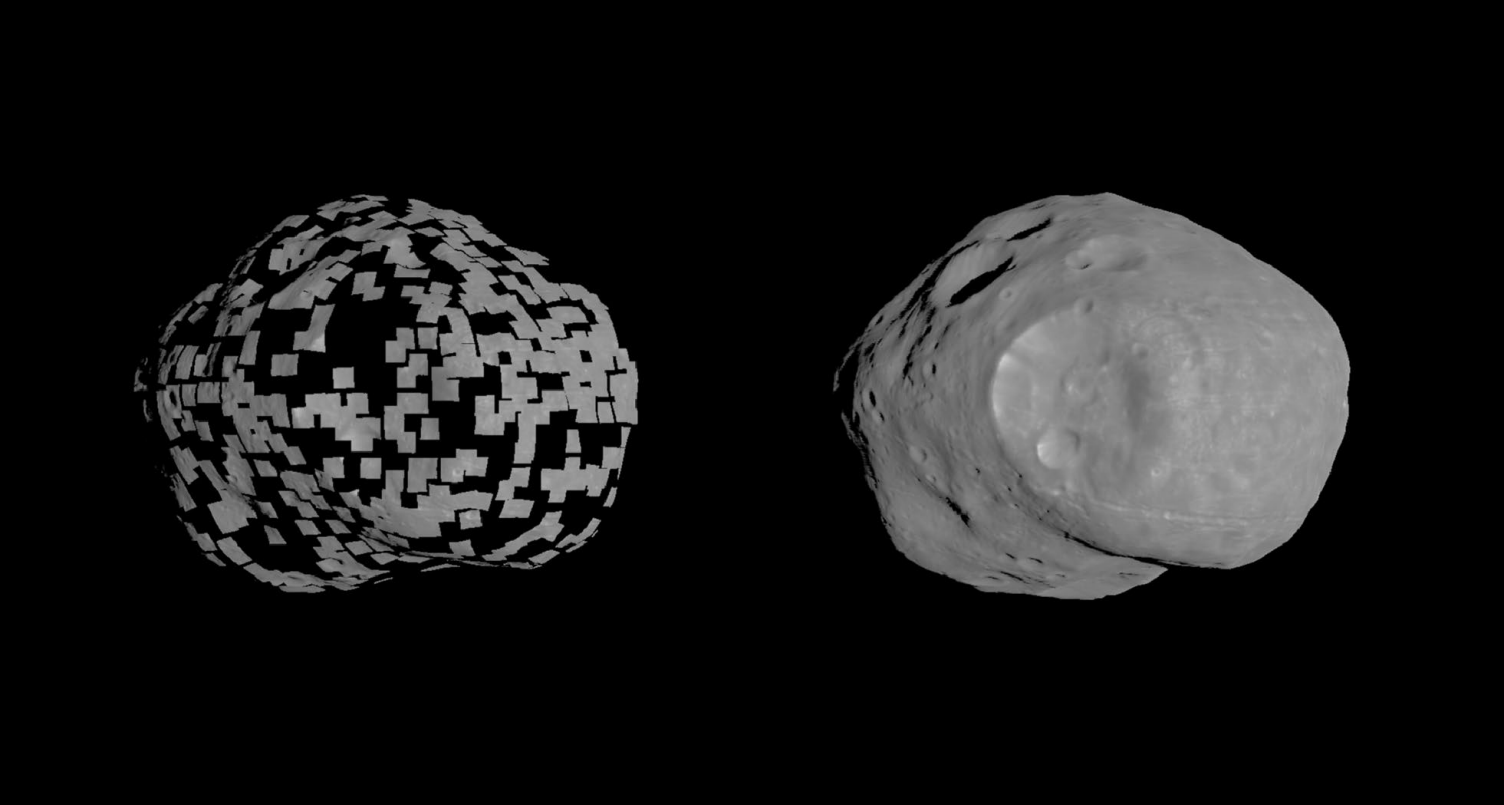
A subset of individual maplets (left) and shape model constructed from many overlapping maplets (right) on Phobos. Maplets of various scales can be combined into a global shape model. In some cases, individual maplets can have a finer ground sample distance than the global model

The SPC maplets are then combined to construct a global shape model (Fig. 2). This process typically starts by interpolating between the points of a lower-resolution starting model to produce a reference surface with a 4 × denser mesh. Vectors from the model center ($\overrightarrow{{V}_{0}}$) and normal to the surface ($\overrightarrow{{N}_{0}}$) are defined at each new vertex. The maplet ensemble is then used to construct the topography of the denser (higher-resolution) model: (1) $\overrightarrow{{N}_{0}}$ is extended a user-defined distance outward from the surface; (2) maplets pierced by $\overrightarrow{{N}_{0}}$ and the distance between the surface and the intersection point along that normal are recorded; (3) a new vector at each vertex is determined as the average of those distances. Interpolated vectors are not modified in cases where no maplets are pierced (e.g., areas of the body not covered by maplets). Generally, this process is repeated several times until the desired resolution is achieved (often limited by the pixel scale and coverage of available images) to create a global shape model. In addition, a global set of high-resolution, regional DTMs can be constructed from the maplet ensemble to produce a product with GSD equal to the input maplets (e.g., Barnouin et al. 2020). In some cases, these regional DTMs provide higher-resolution topography (smaller GSD) than the global shape model.

Ultimately, the SPC shape modeling process culminated in global shape models of Phobos and Deimos, the relative albedo across each surface, higher-resolution regional digital terrain models, and a coregistered collection of images from six spacecraft that covers a wide range of pixel scales, incidence, emission, and phase angles.

### Image data

Our shape modeling effort incorporated imaging data from cameras on six spacecraft (five missions) (Table 1): the Viking Orbiter Visible Imaging Subsystem (VIS) (four cameras, two on each spacecraft, Viking Orbiter 1 and 2), the Phobos 2 VideoSpectrometric Camera (VSK), the Mars Global Surveyor (MGS) Mars Orbiter Camera (MOC) narrow-angle camera, the Mars Express (MEX) High Resolution Stereo Camera high-resolution stereo head (hereafter, HRSC) and the super resolution channel (hereafter, SRC), and the Mars Reconnaissance Orbiter (MRO) High Resolution Imaging Science Experiment (HiRISE). All of the data are publicly available, through either the NASA Planetary Data System or the European Space Agency Planetary Science Archive. We did not incorporate Mariner 9 images as these data are comparable to and lower resolution than the Viking data and the additional effort required to incorporate them was deemed not worth the yield at this time.

**Table 1:** Phobos and Deimos data available for this effort

Years	Mission	Agency	Instrument	Sensor	Type	Phobos coverage	Deimos coverage	Archive location^b^	Dataset reference	Instrument reference
1975–1980	Viking Orbiter 1&2	NASA	VIS	Vidicon	Framing	Global	Hemispherical (sub-Mars)	NASA PDS	Duxbury 1978; Veverka 1978	Wellman et al. 1976
1989	Phobos 2	Soviet space program	VSK NAC	CCD	Framing	Hemispherical (anti-Mars)	n/a	NASA PDS	Murchie and Erard 1996	Murchie and Erard 1996
1998	MGS	NASA	MOC NAC	CCD line array	Pushbroom	Hemispherical (sub-Mars)	Hemispherical (sub-Mars)	NASA PDS	Murchie 1999	Malin et al. 2010
2004–2016^a^	MEX	ESA	SRC	CCD array sensor	Framing	Global	Hemispherical (sub-Mars)	ESA PSA	Wählisch et al. 2010; Witasse et al. 2014	Jaumann et al. 2007
			HRSC	9 CCD line sensors	Pushbroom	Global	n/a			
2008–2009	MRO	NASA	HiRISE	CCD array sensor	Pushbroom	Hemispherical (sub-Mars)	Hemispherical (sub-Mars)	NASA PDS	Thomas et al. 2011	McEwen et al. 2007

^a^HRSC and SRC images of Phobos and Deimos continue to be acquired. This effort incorporated images taken through 7 August 2016

^b^See “Availability of data and materials” for links to these archives

We examined and catalogued ~ 3400 and ~ 950 images of Phobos and Deimos, respectively, in which the moons appear at least 10 pixels across to determine the subset of images to be used to construct the models. Both panchromatic and color filter/channel images were considered. Additional files 1 and 2 provide lists for Phobos and Deimos, respectively, of the images used to construct the model, images registered to but not used to construct the model, and images considered but not used. Images that were not used were typically of poor quality (e.g., saturated, contained artifacts), had only a small fraction of the moon in the scene, or were too low resolution to improve the modeled topography. Tables 2 and 3 summarize the number and pixel scales of images used to construct the new shape models. The Deimos models incorporate images from only four missions. Phobos 2 did not image Deimos. HRSC images of Deimos have relatively low pixel scales and do not contribute a different view from SRC. A single MOC Deimos image was included. SRC suffers from an astigmatism that can cause blurring and ghosting effects (Oberst et al. 2008), effectively decreasing the resolution of the images. Despite this issue, the spatial and viewing conditions coverage afforded by the SRC images make this dataset critical for high-resolution modeling of Phobos’ topography. While we typically allowed images with pixel scales up to 4 × the maplet GSD to contribute to the solution, SRC image pixel scales were often limited to 2 × the maplet GSD.

**Table 2:** Phobos images used to construct SPC model

	Viking	Phobos2	MOC	SRC	HRSC	HiRISE
# of images used to build model	216	7	4	1611	520	24
Coarsest image pixel scale (m)	194.3	196.8	35.9	167.5	198.8	6.7
Finest image pixel scale (m)	2.8	44.1	1.5	2.7	4.1	5.8
Median image pixel scale (m)	16.7	67.1	4.7	46.4	58.1	6.2
Mean image pixel scale (m)	49.7	81.9	11.7	60.3	71.4	6.2
Oldest image used (UTC)	1976 SEP 18 08:39:42.748	1989 FEB 21 13:01:23.447	1998 AUG 07 14:11:33.990	2004 MAY 18 08:34:17.052	2004 MAY 18 08:30:58.549	2008 MAR 23 20:55:24.480
Most recent image used (UTC)	1978 OCT 19 09:06:32.425	1989 MAR 25 10:40:16.378	2003 JUN 01 17:30:11.170	2016 AUG 07 15:52:22.060	2016 JUL 24 22:55:20.853	2008 MAR 23 21:05:27.035
Min # of maplets per image	4	7	12	4	4	6
Max # of maplets per image	949	30	92	816	1103	494
Median # of maplets per image	41	13	50	39	51	243
Mean # of maplets per image	120	15	51	87	134	232

**Table 3:** Deimos images used to construct SPC model

	Viking	MOC	SRC	HiRISE
# of images used to build model	95	1	227	9
Coarsest image pixel scale (m)	171.0	85.4	162.3	22.6
Finest image pixel scale (m)	14.6	85.4	87.3	19.7
Median image pixel scale (m)	58.5	85.4	105.9	22.6
Mean image pixel scale (m)	68.0	85.4	108.9	21.6
Oldest image used (UTC)	1976 AUG 16 07:15:50.463	2006 JUL 10 02:04:55.000	2004 OCT 22 08:06:14.355	2009 FEB 21 13:54:57.399
Most recent image used (UTC)	1978 JUN 02 15:46:03.015	2006 JUL 10 02:04:55.000	2016 JUN 23 11:07:33.631	2009 FEB 21 19:29:59.458
Min # of maplets per image	3	42	5	39
Max # of maplets per image	115	42	40	85
Median # of maplets per image	59	42	13	77
Mean # of maplets per image	60	42	15	67

**Fig. 3:**
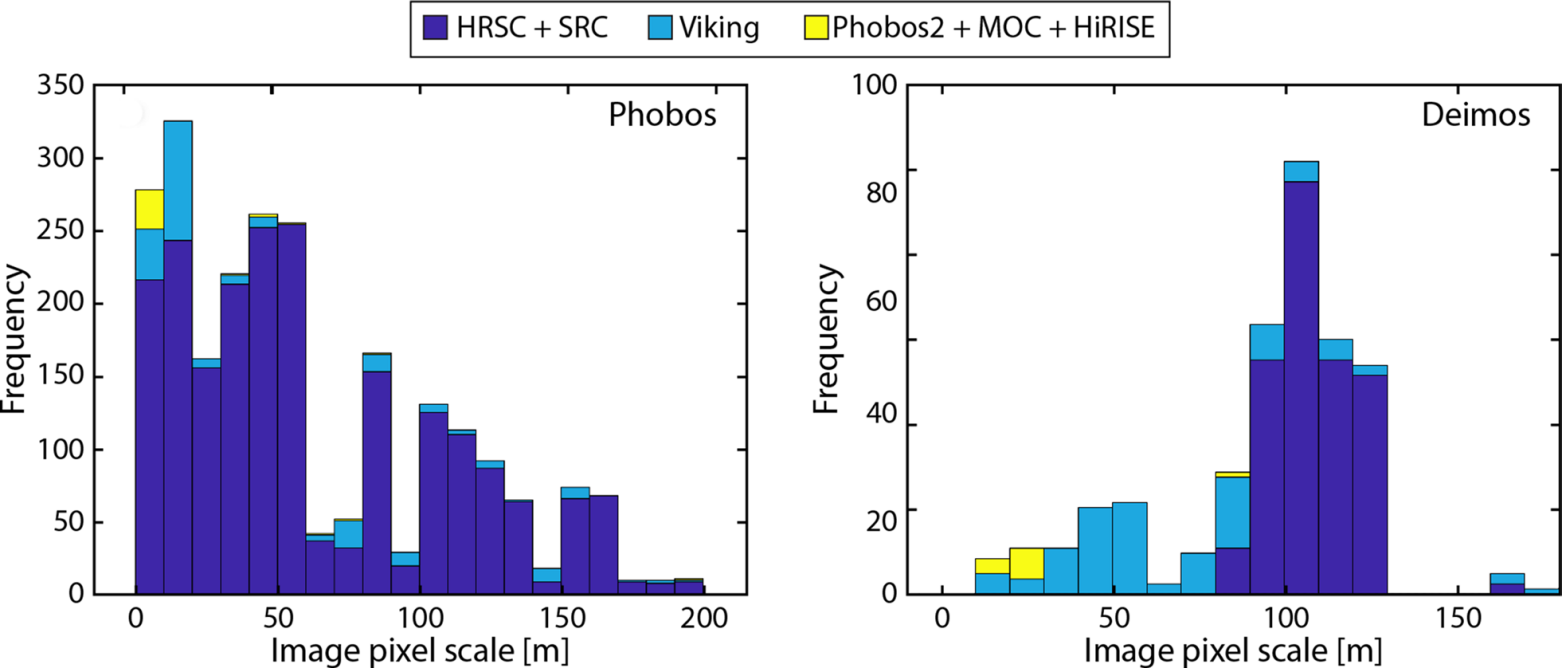
Histogram of the image pixel scales used to construct the Phobos (left) and Deimos (right) SPC shape models. The Phobos model incorporates 2382 images and the Deimos model incorporates 332 images

**Fig. 4:**
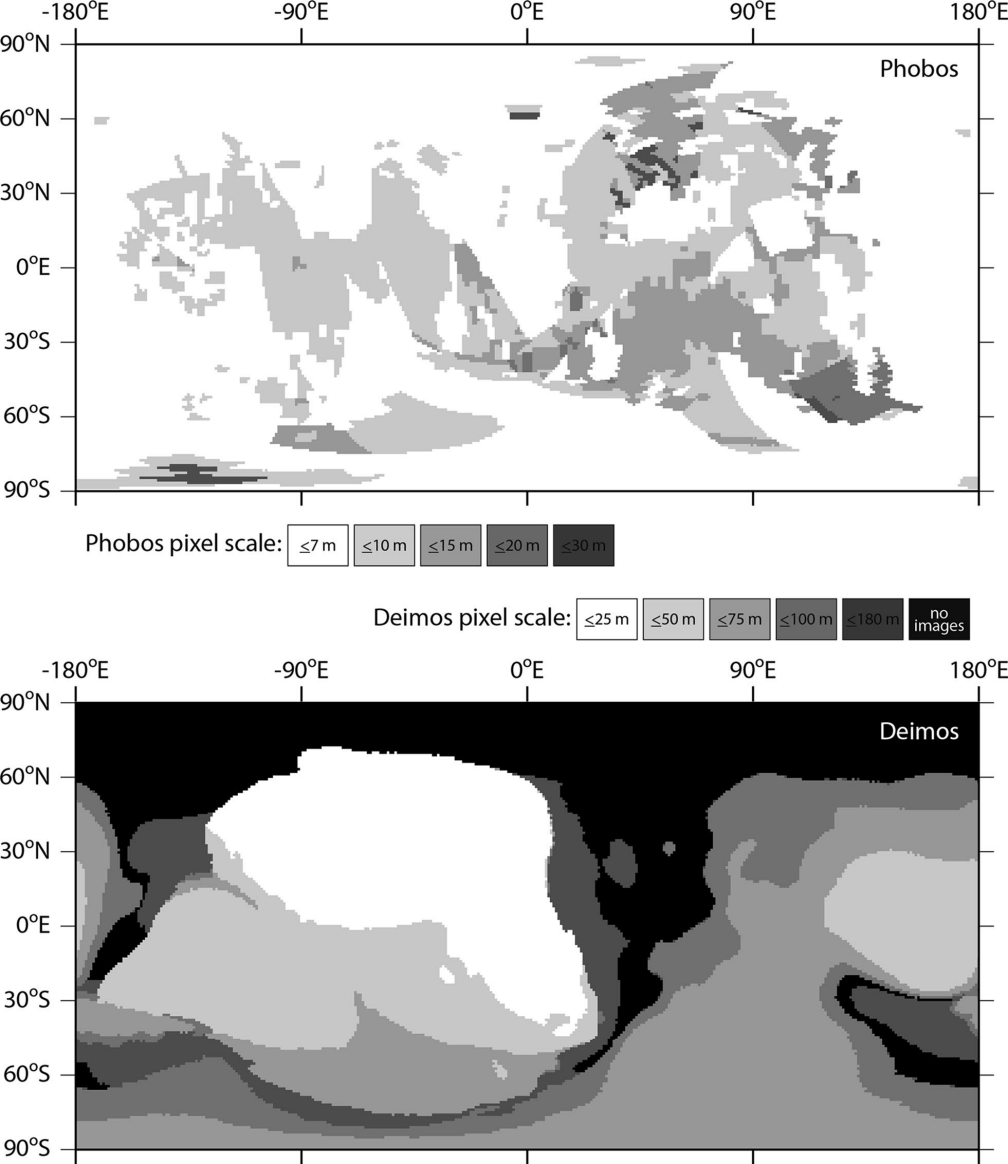
Coverage maps for Phobos (top) and Deimos (bottom) for the images ≤ 80º incidence angle, and ≤ 70º emission angle used to make the SPC models. Black indicates areas not covered by images used to make the Deimos model (Phobos has global image coverage). Most of Phobos is covered by images ≤ 10 m pixel scale. Much of one hemisphere of Deimos (from approximately −170ºE to 30ºE) is covered by images ≤ 50 m pixel scale. The region between ~ 50ºE and 190ºE is covered primarily by a single image (f507a01). Its limb was used to constrain the model and it was incorporated into a few maplets at higher southern latitudes (> 30º), but it could not support maplets on its own elsewhere

The new Phobos and Deimos shape models were constructed from 2382 and 332 images, respectively. Figure 3 illustrates the distribution of image pixel scales used to make the models. Phobos is globally covered by images at ~ 10-m pixel scale, and Deimos is hemispherically covered at ~ 50-m pixel scale (Fig. 4). We adopt these values as the representative best pixel scales for the two datasets. The pixel scale of an image depends on the camera optics and the distance between the moon and the spacecraft. The spatial coverage obtained by a mission depends on the number of imaging opportunities and the spacecraft’s orbit about Mars, and is further constrained because both moons are tidally locked to Mars. Viking Orbiter was and MEX is in an elliptical orbit enabling numerous flybys of Phobos. Global coverage of Phobos was obtained by VIS, HRSC, and SRC, and these datasets are the most comprehensive. The short-lived Phobos 2 mission imaged only the anti-Mars hemisphere of Phobos at moderate resolution. MGS and MRO were limited to imaging Phobos’ sub-Mars hemisphere a small number of times, but MOC and HiRISE yielded some of the highest-resolution images. The highest-resolution image of Phobos is a MOC image with a pixel scale of 1.5 m.

Deimos orbits farther from Mars than Phobos, and therefore farther for most spacecraft observing opportunities. The images of Deimos suitable for shape modeling cover one hemisphere. There is one Viking image with a pixel scale ~ 34 m that offers a well-resolved view of the trailing hemisphere (90ºE). As it was a single image, we did not use it to generate topography of that hemisphere. It was linked to the model by a few maplets poleward of 30ºS, and its limb was used to constrain the model. As with Phobos, the VIS and SRC datasets are the most comprehensive, with the VIS images having generally smaller pixel scales than SRC. HiRISE provides a handful of high-resolution images (~ 20 m pixel scale) of the sub-Mars side. The three highest-resolution images of Deimos were taken by Viking Orbiter 2 during a close flyby and have pixel scales from 1.1 to 1.4 m. These images are so much higher resolution than the rest of the set that they were not used to derive topography for our model; however, we registered them to the rest of the dataset, providing precise knowledge of their location on the surface.

### Quality of image sets relative to ideal SPC imaging criteria

**Fig. 5:**
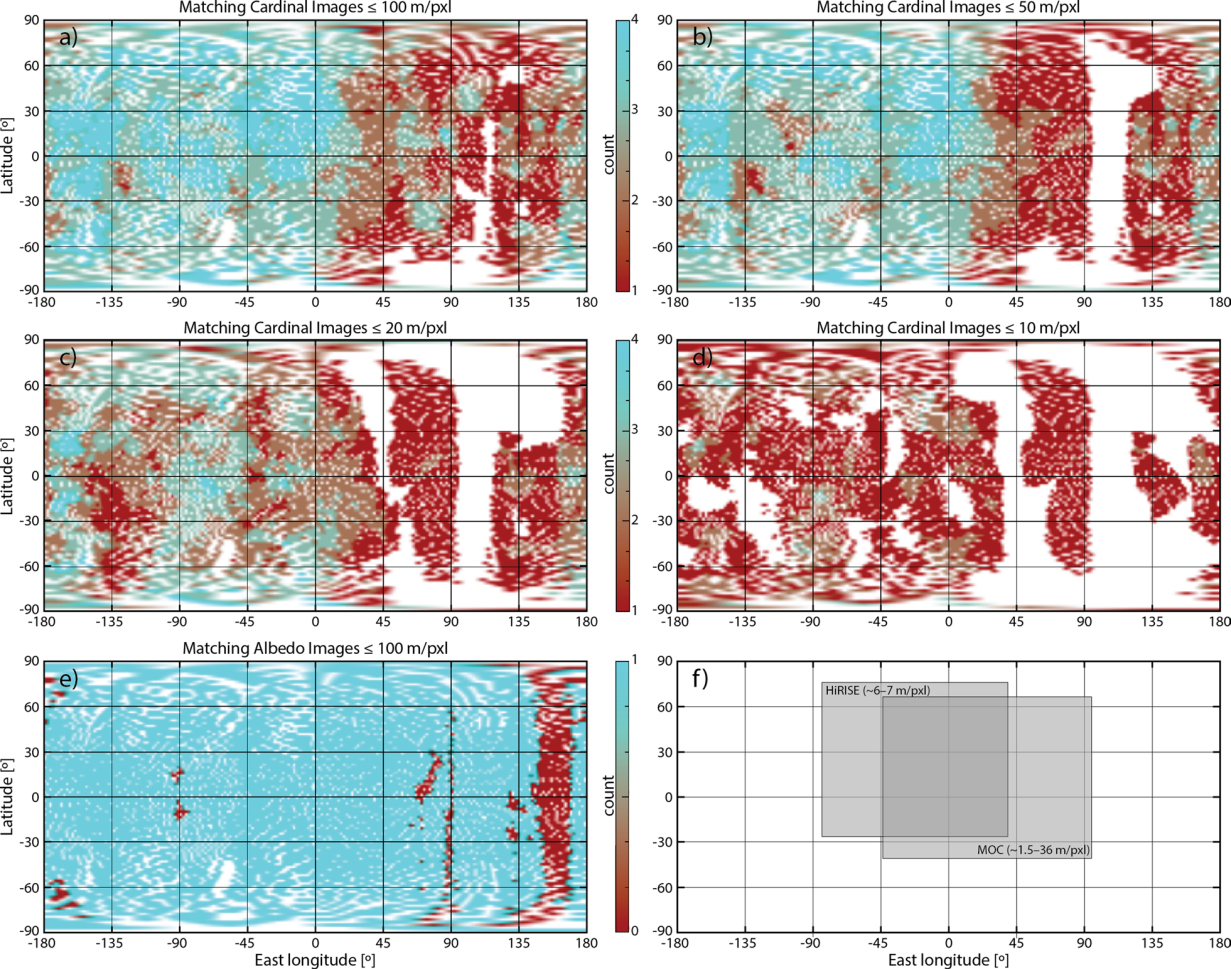
An analysis of the quality of the Phobos input image set relative to ideal SPC imaging criteria (Al Asad et al. 2021). **a**–**d** Number of framing images that match the cardinal directions required by SPC at different pixel scales. **e** Number of framing images with pixel scales ≤ 100 m that match the albedo image requirements of SPC. **f** Approximate coverage of HiRISE and MOC pushbroom images

**Fig. 6:**
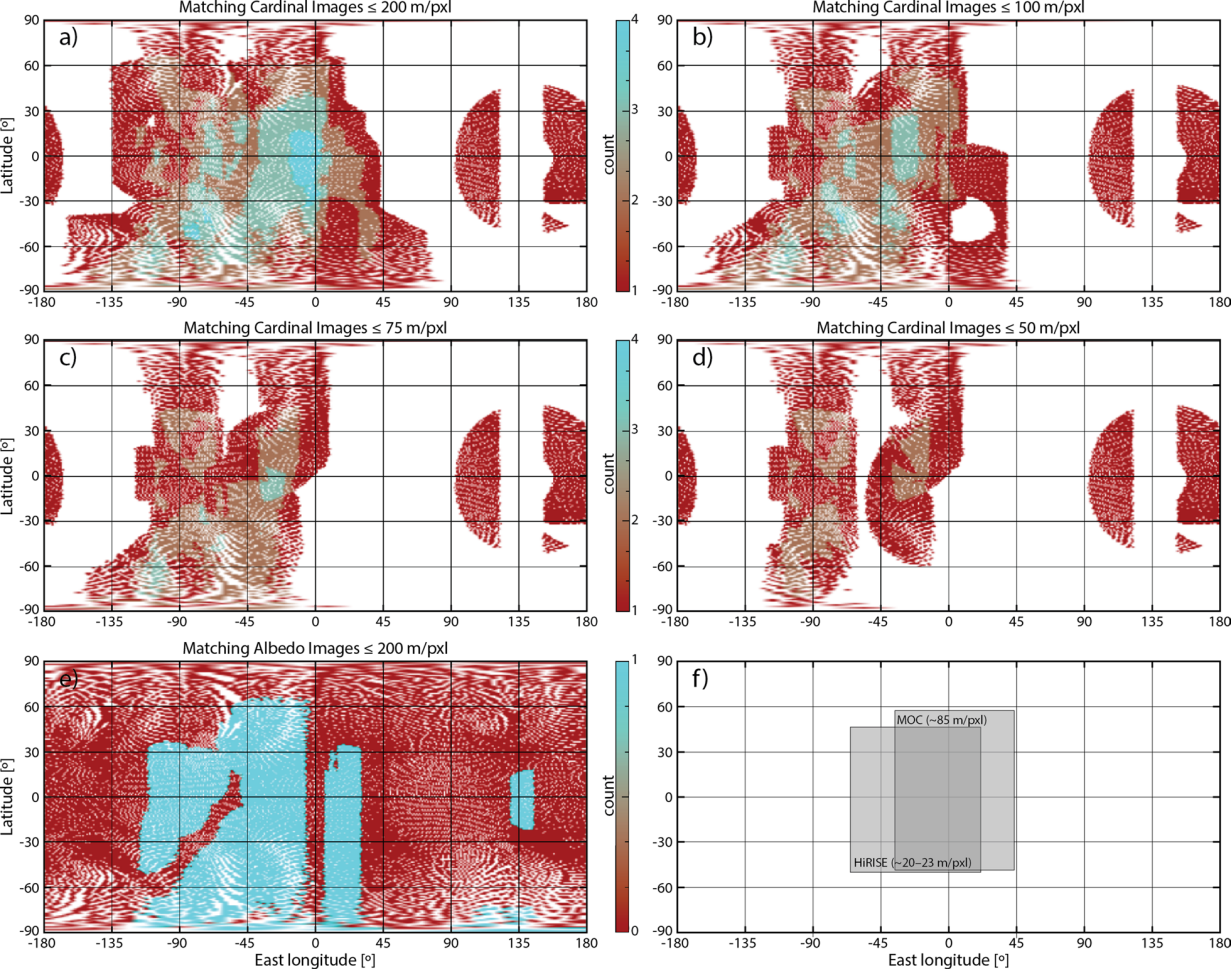
An analysis of the quality of the Deimos input image set relative to ideal SPC imaging criteria (Al Asad et al. 2021). **a**–**d** Number of framing images that match the cardinal directions required by SPC at different pixel scales. **e** Number of framing images with pixel scales ≤ 200 m that match the albedo image requirements of SPC. **f** Approximate coverage of HiRISE and MOC pushbroom images

The quality of SPC models is inextricably linked to the input images, in particular the viewing conditions. Based on empirical tests, the OSIRIS-REx mission determined that, for optimal results, each piece of terrain (maplet) in an SPC shape model must contain an “ideal” imaging dataset: (1) at least four images with different observer elevations and azimuths (measured as being in separate quadrants or cardinal directions); (2) varying incidence angles among these four images; (3) a fifth “albedo” image (emission ~ 0º, incidence ~ 10º), which can be lower in resolution, to aid the relative albedo solution; (4) comparable pixel scales across the five-image set, with 1–2 images at or below the maplet GSD and no image > 5 × the maplet GSD (Al Asad et al. 2021; Palmer et al. 2022). A “sufficient” imaging dataset is one that meets many, but not all, of the ideal criteria (e.g., Weirich et al. 2022). As the Phobos and Deimos datasets were acquired during opportunistic flybys, the images were not tuned to meet these ideal criteria. However, given the large number of flybys during which data were acquired, coverage approaching these criteria was obtained globally for Phobos (Fig. 5) and hemispherically for Deimos (Fig. 6).

We assessed how well the input images satisfied the ideal SPC criteria using an OSIRIS-REx tool (e.g., Al Asad et al. 2021). The OSIRIS-REx spacecraft has only framing imagers; as such, the tool is not set up currently to handle pushbroom datasets. As a consequence, our assessments were performed on the available framing images. We estimated the approximate coverage of HiRISE and MOC images for both bodies to compare against the framing datasets. These pushbroom images add additional viewpoints to the existing framing datasets that increase the regions that satisfy the ideal SPC criteria. We did not assess the coverage contribution of the Phobos HRSC pushbroom images as they were taken concurrently with SRC images, so the viewing conditions were captured by the framing image analysis. HRSC images improve the qualifying coverage when they were taken during sequences where Phobos fills the SRC field of view.

Figure 5 illustrates how well the input images satisfy the ideal SPC criteria for Phobos. An ideal SPC image dataset (image from all 4 cardinal directions plus albedo) is achieved for most of the globe for pixel scales ≤ 100 m. A sufficient SPC image dataset (images from ≥ 2 cardinal directions plus albedo) is achieved for most of the globe for pixel scales ≤ 50 m. The trailing hemisphere (centered on 90ºE) has the worst qualifying coverage. The addition of HiRISE and MOC images to the assessment would increase the qualifying coverage significantly on the sub-Mars hemisphere (Fig. 5f), improving the ideal SPC image dataset pixel scale to ~ 50 m and the sufficient SPC image dataset pixel scale to ~ 20 m.

Figure 6 illustrates how well the input images satisfy the ideal SPC criteria for Deimos. An ideal SPC image dataset (image from all 4 cardinal directions plus albedo) is achieved in a region near the sub-Mars point for pixel scales ≤ 200 m. A sufficient SPC image dataset (images from ≥ 2 cardinal directions plus albedo) is achieved hemispherically for pixel scales ≤ 100 m. The addition of HiRISE and MOC images to the assessment would increase the qualifying coverage significantly on the sub-Mars hemisphere (Fig. 6f), broadening the coverage of the ideal SPC image dataset for pixel scales ≤ 200 m, and improving the sufficient SPC image dataset pixel scale to ~ 75 m hemispherically.

### Expected accuracy and precision of the SPC models

Accuracy is a measure of the absolute position uncertainty of the surface in three dimensions, and is important for assessing the global shape. Precision is a measure of local point-to-point uncertainties in the topography (i.e., the uncertainty associated with a profile across a crater). Historically, it has been difficult to determine the accuracy and precision of topographic models derived from many methods. Potential sources of uncertainty abound (e.g., spacecraft position, pointing, pole position, rotation rate, center of mass, etc.), and generally no “truth” exists to evaluate against. Recently, however, multiple studies have evaluated the strengths and limitations of the SPC method, and these studies provide a framework for characterizing the accuracy and precision of these new models.

### Assessments of accuracy and precision from previous SPC models of irregular bodies

A few studies have analyzed the accuracy and precision of SPC models, and Table 4 summarizes their findings. These studies rely on comparisons between SPC models and altimetric data (Roberts et al. 2014b; Craft et al. 2020; Al Asad et al. 2021), testing with simulated images (Barnouin et al. 2020; Craft et al. 2020; Al Asad et al. 2021; Palmer et al 2022; Weirich et al 2022), and relative error analysis (Park et al. 2019).

**Table 4:** Summary of SPC accuracy and precision assessments from previous efforts

Model	Notes	Accuracy^a^	Precision^b^	Reference
Eros	SPC vs shifted NLR tracks	n/a^c^	0.5 ×	Roberts et al. 2014b
Eros	SPC vs NLR ranges to surface	2 ×	n/a	Barnouin personal communication
Test wall	SPC vs altimetry	n/a	1.5 ×	Craft et al. 2020
Bennu	SPC vs OLA; SPC-derived metrics	1.3–4.5 ×	6 ×	Al Asad et al. 2021
Test Bennu	SPC vs truth model	1–2.5 ×	n/a	Barnouin et al. 2020; Weirich et al. 2022
Ceres	Relative error analysis; modeling from independent sets of images	n/a	0.6 ×	Park et al. 2019
Rule of Thumb	Assuming ideal SPC image criteria are met	1–5 ×	0.5–6 ×	Range from values above

^a^Values with respect to the representative mean pixel scale of the images used to make the SPC model that satisfy the sufficient SPC imaging criteria

^b^Values with respect to the representative best pixel scale of the images used to make the SPC model

^c^n/a indicates a value not discussed in the reference

We normalize accuracy and precision estimates of these previous studies by the pixel scale of the images used to build a given model to allow cross-model comparisons and derive rules of thumb for SPC model quality. Accuracy estimates are normalized by the representative mean pixel scale of the images that satisfy the sufficient SPC criteria (see “Quality of Image Sets Relative to Ideal SPC Imaging Criteria”), as the ensemble of images controls the stereo solution that determines absolute position. Precision estimates are normalized by the representative best pixel scale, as the highest-resolution images drive the photometric solution that determines the local topography.

### Comparisons between SPC and altimetric data

Four studies assessed the quality of SPC models by directly comparing the models to altimetric data:

1. SPC vs NLR topography (NEAR at Eros). The first study used data acquired by the NEAR mission, which visited the asteroid Eros. A global SPC model of Eros was constructed from Multi-Spectral Imager (MSI) data (Gaskell 2008). The NEAR Laser Altimeter (NLR) collected altimetric data at Eros. After shifting NLR tracks to align with the shape model, Roberts et al. (2014b) compared 32 profiles taken across smooth areas of the Eros SPC global shape model to the NLR tracks. The comparison yielded a median root mean square (RMS) difference precision value of 1.7 m, ~ 0.5 × the images’ representative best pixel scale (3.2 m, Roberts et al. 2014a; personal communication Olivier Barnouin).
2. SPC vs NLR spacecraft range (NEAR at Eros). The second study compared the range between the NEAR spacecraft and the surface as measured by NLR with the range estimated from the SPC solution (model, spacecraft position, and spacecraft pointing). The SPC model has a 21-m accuracy, which is ~ 2 × the images’ representative mean pixel scale (10.8 m, personal communication Olivier Barnouin).
3. SPC vs Truth Wall (OSIRIS-REx ground testing). The third study compared altimetric measurements of a physical wall made to simulate an asteroid surface to an SPC model of the wall constructed out of flight-like images. Those comparisons yielded topographic height precision ~ 1.5 × the images’ representative best pixel scale (Craft et al. 2020).
4. SPC vs OLA (OSIRIS-REx at Bennu). The fourth study compared SPC model and altimetric datasets collected by the OSIRIS-REx mission at asteroid Bennu. The OSIRIS-REx team compared the Bennu SPC shape model constructed from the OCAMS rendezvous images to data from the OSIRIS-REx Laser Altimeter (OLA). Most of Bennu’s surface was covered with 3–4 images with 15 cm average pixel scale (i.e., representative mean pixel scale), and 2–3 images with 5 cm average pixel scale (i.e., representative best pixel scale) with the desired range of viewing geometry, plus an additional albedo image (Al Asad et al. 2021). Additional uncertainties were folded into the resulting models, including the Bennu pole position and rotation rate (which accelerated over the course of the encounter). The comparison employed early OLA data where the OLA footprint ranged from 30–90 cm. The comparison between the SPC model and these OLA data found an accuracy in Bennu’s reported radius (+ 68/-20 cm, (Al Asad et al. 2021)) of 1.3–4.5 × the representative mean pixel scale. The RMS precision was 68 cm (Al Asad et al. 2021). Additional analysis shows that much of this uncertainty is due to SPC underrepresenting the edges of the rocks that cover Bennu’s surface. Restricting the RMS analysis to the 80% central values, to account for the effects of rocks, yields an RMS precision ~ 30 cm, which is ~ 6 × the images’ representative best pixel scale.

### Comparisons between SPC and truth using simulated image datasets

Prior to arrival at Bennu, OSIRIS-REx also used a controlled, flight-like dataset of simulated images to evaluate the expected accuracy of their flight shape model. Members of the OSIRIS-REx team constructed an SPC model of Bennu from synthetic OSIRIS-REx Camera Suite (OCAMS) test images designed to meet the imaging criteria (Craft et al. 2020; Al Asad et al. 2021) described in the previous section. In this experiment, the Bennu pole position, rotation rate, and center of mass were all known values. The resulting SPC model produced the locations of surface features in 3D with a 0.1-m RMS uncertainty when compared with the synthetic “truth” model (Barnouin et al. 2020), which is 2.5 × the images’ representative mean pixel scale. Additional studies using this dataset found that the accuracy of the SPC model was < 1 × and ~ 1 × the images’ representative mean pixel scale of an ideal or sufficient SPC image dataset, respectively (Weirich et al. 2022).

### Relative error analysis

Finally, an SPC model of Ceres with a 100-m GSD was derived from Dawn Framing Camera orbital images with global coverage at 35-m pixel scale. The average height error of the model was estimated to be 10.2 m, and nearly 90% of the surface had total height errors < 20 m (Park et al. 2019), which is a precision of ~ 0.6 × the pixel scale of the high-resolution images.

### Rules of thumb for SPC model accuracy and precision

Taking the above assessments into account, a rule of thumb for SPC model accuracy is 1–5 × the representative mean pixel scale of the images used to make the model that satisfy the sufficient SPC imaging criteria, and a rule of thumb for SPC model precision is 0.5–6 × the pixel scale of the representative best images used to make the model. The accuracy and precision will degrade if some of the imaging criteria are not satisfied. We apply these rules of thumb to the Phobos and Deimos imaging datasets (Tables 2, 3; Fig. 4) to get an idea of the best-case accuracy and precision for our SPC models. For Phobos, the sufficient imaging criteria are met globally at ~ 20 m pixel scale. For Deimos, the sufficient imaging criteria are met hemispherically at ~ 75 m pixel scale.

### SPC model accuracy and precision estimates from the input datasets

The mean pixel scale of the images used to construct the global, 10-m GSD Phobos maplets is ~ 20 m (maplets were limited to use images with pixel scales < 4 × the maplet GSD except for SRC, which was limited to pixel scales < 2 × the maplet GSD). Phobos is covered globally with images satisfying the sufficient SPC criteria at this pixel scale (Fig. 5). Thus, 20 m is taken as the representative mean pixel scale of the dataset with which to estimate the accuracy. The ~ 10-m pixel scale (Fig. 4) global coverage of Phobos is taken as the representative best pixel scale of the dataset with which to estimate the precision. Application of the accuracy rule of thumb (1–5 ×) to the representative mean pixel scale indicates that the Phobos image dataset should yield an accuracy of 20–100 m. Application of the precision rule of thumb (0.5–6 ×) to the representative best pixel scale indicates that the Phobos image dataset should yield a precision of 5–60 m.

Deimos has hemispherical 50-m pixel scale coverage (Fig. 4) but is covered hemispherically with images satisfying the sufficient SPC criteria at a 75-m pixel scale (Fig. 6). Thus, 75 m is taken as the representative mean pixel scale of the dataset. The ~ 50-m hemispherical coverage is taken as the representative best pixel scale of this dataset. Application of the accuracy rule of thumb to the representative mean pixel scale indicates that the Deimos image dataset should yield an accuracy of 75–375 m on the modeled hemisphere. Application of the precision rule of thumb to the representative best pixel scale indicates that the Deimos image dataset should yield a precision of 25–300 m on the modeled hemisphere.

### SPC-derived metrics

Several types of uncertainty are recorded as part of the SPC process that can be used to assess the quality of the resulting model. Al Asad et al. (2021) describe these metrics and how they were used by OSIRIS-REx to assess the quality of Bennu SPC models. We use three of these metrics here: maplet residuals, maplet formal uncertainty, and vertex sigma. A maplet’s stereo solution is an estimate of its absolute position in space based on all images in that maplet. For maplets containing more than two images, each image pair yields a different stereo solution; the RMS of the stereo solutions of each image pair is defined as the maplet’s stereo solution. The maplet residual is the RMS of the difference between a maplet’s stereo solution and its pixel position in the images. This metric gives a measure of the positional uncertainty between the shape and the images measured at each maplet center and is independent of the derived topography within the maplets.

The maplet formal uncertainty is a measurement of the internal consistency of the SPC model (Weirich et al. 2022). This value is derived from the covariance matrix used to solve for landmark position at each maplet center during the geometry estimation of spacecraft position (Gaskell 2006; Gaskell et al. 2023). Each image contained in a maplet can be used to predict the location of the maplet in space. The RMS of the difference between the predicted and actual location of a maplet is its formal uncertainty (Weirich et al. 2022). The covariance matrix is updated over many iterations to minimize the maplet residual; the maplet formal uncertainty is therefore correlated with the maplet residual. Testing has shown that a stable solution converges by 10 iterations, though convergence can often be achieved with fewer iterations (Palmer et al. 2022). Weirich et al. (2022) recently demonstrated with truth and reconstructed Bennu SPC test models that the mean formal uncertainty for the maplet ensemble is a good quantitative estimate of the model fidelity. The formal uncertainty always corresponded within a factor of two with the truth model, slightly overestimating the fidelity of the model when the image suite used in the reconstruction was sufficient for SPC, and providing an excellent estimate of the accuracy when the SPC imaging criteria were met. The mean maplet formal uncertainty can be taken as a lower limit of the overall model accuracy.

Vertex sigma is the standard deviation of the heights of overlapping maplets, providing a measure of radial uncertainty at each vertex. Systematic comparisons between the Bennu SPC shape model and OLA data found that the regional uncertainties are ~ 5 × the value of vertex sigma when the SPC imaging criteria are met. This “scaled vertex sigma” metric is dependent on the derived topography within the maplets, and thus gives a measure of the precision of the local topography when the SPC imaging criteria are met. In a situation where viewing and lighting conditions are limited, the scaled vertex sigma is a poor measure of precision because the topographic variability between overlapping maplets is artificially small.

### Comparison to images

**Fig. 7:**
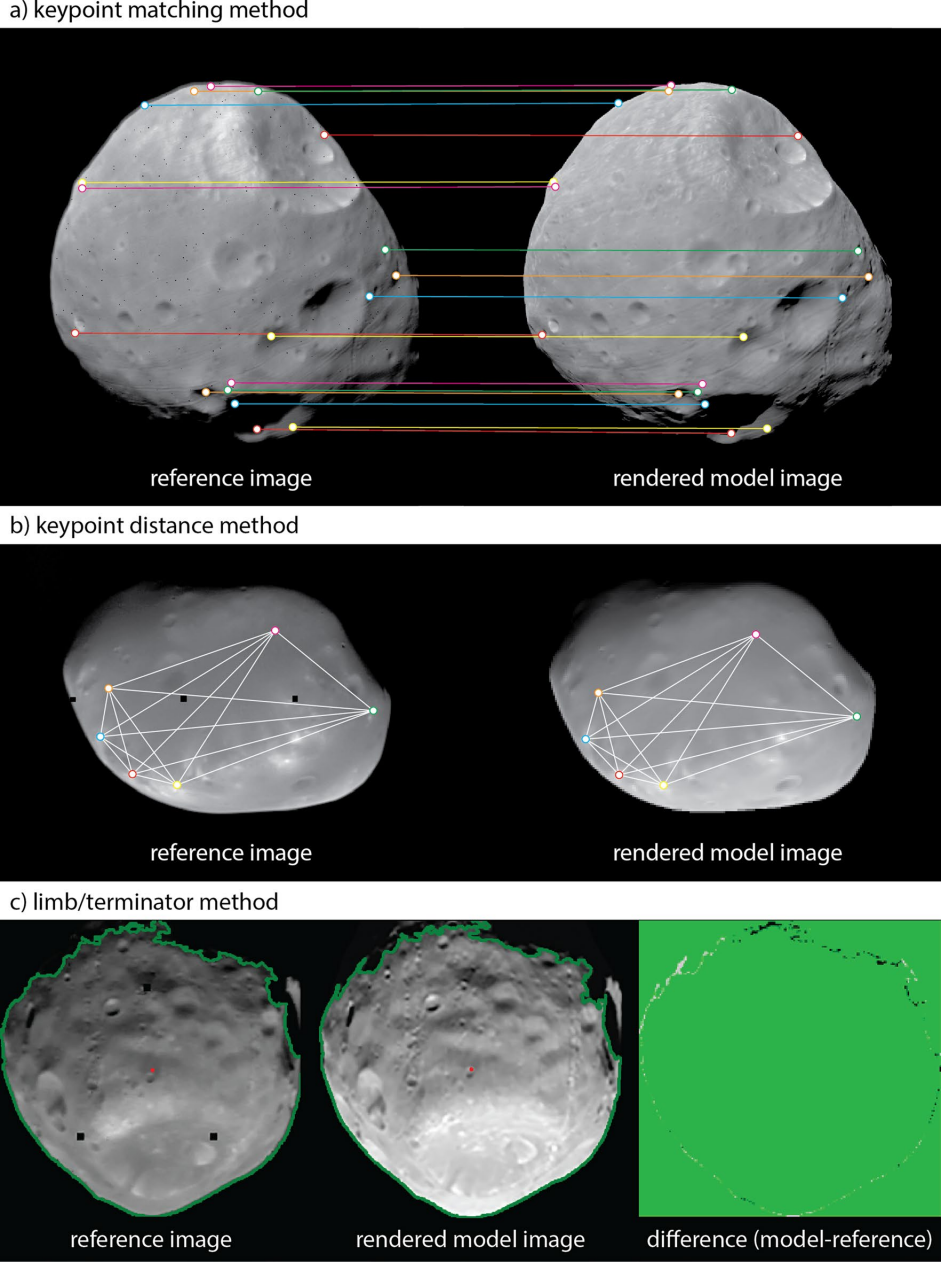
Examples of three comparisons between rendered images of the shape model with spacecraft images to assess model quality. **a** Illustration of the keypoint matching method using SRC image H3245_0006sr2 of Phobos. Matched keypoints on the reference image (left) and the rendered model (right) are connected by lines. **b** Illustration of the keypoint distance method using Viking Orbiter image f428b22 of Deimos. The collection of distances between all keypoints is shown on the reference image (left) and the rendered model (right). **c** Illustration of the limb/terminator method using Viking Orbiter image f123b03 of Phobos. Limb and terminator positions are outlined in green on the reference image (left) and on the rendered model (center). A difference image (right) shows mismatches in black or grey

The quality of the model can also be assessed by directly comparing rendered images of the shape model (which include the relative albedo from SPC) to reference images taken by spacecraft. We used three such comparisons to gain additional insight into shape model accuracy and potential scale uncertainty. Applying these methods to > 30 images with a variety of views will yield statistically meaningful results (e.g., Al Asad et al. 2021). The first and second comparisons rely on an automated matching of surface features, or keypoints, to identify scale uncertainty in the model (i.e., whether the model is too large or too small). In the second comparison, referred to as the “keypoint matching method”, the rendered image is rotated, translated, and scaled to minimize the differences in locations of the matched keypoints in the rendered image versus the reference image (Fig. 7a). The derived scale factor can be multiplied by the average body radius to calculate the scale uncertainty (e.g., the difference between the size of the model and the size of the body in the reference image). The standard deviation of the scale uncertainty gives a measure of model accuracy. In the third and final comparison, referred to as the “keypoint distance method”, the distances measured between all keypoints in a rendered image are compared to the distances measured between all keypoints in a reference image (Fig. 7b). A median residual value is recorded for each image. The median residual of these values from many images gives a measure of model scale uncertainty. The RMS of these recorded values gives a measure of the model accuracy. The third comparison, referred to as the “limb/terminator method”, analyzes the positions of the limb and terminator in a rendered image versus those in a reference image (Fig. 7c). The limb/terminator method provides a measure of overall shape model accuracy, as well as any potential scale uncertainty in the model. All three of these techniques were used by OSIRIS-REx to evaluate the model accuracy and absolute scale uncertainty for SPC models of Bennu (Al Asad et al. 2021).

### Input assumptions

We used the pole, rotation, and libration parameters for Phobos and Deimos (Archinal et al. 2011) that were contained in the latest planetary constants kernel available from the NASA Navigation and Ancillary Information Facility (NAIF) at the time of model construction (pck00010.tpc; Bachman 2011). These orientation models are out of date in the current epoch (Archinal et al. 2018), although Stark et al. (2017) note, “The current accuracy is sufficient for cartographic purposes but might need improvement for tasks like high-precision landing on Phobos". Newer solutions are now available (e.g., Stark et al. 2017; Burmeister et al. 2018; Jacobson et al. 2018) that have since been adopted by the IAU (Archinal et al. 2018; 2019) but only recently made their way into an updated planetary constants kernel at NAIF (pck00011_n0066.tpc; Bachman 2022). We estimate that the differences in the pole and prime meridian between the old and new solutions could account for up to 25 m of error in the position of features across Phobos and 2 m across Deimos; these values are within the error estimates for our models, which are discussed later in this work (see “Phobos model quality assessment”, “Deimos model quality assessment”). Incorrect pole positions tend to lead to models that are slightly too large. A future update to the Phobos and Deimos models presented here would benefit from use of the updated parameters.

### Phobos shape model

**Fig. 8:**
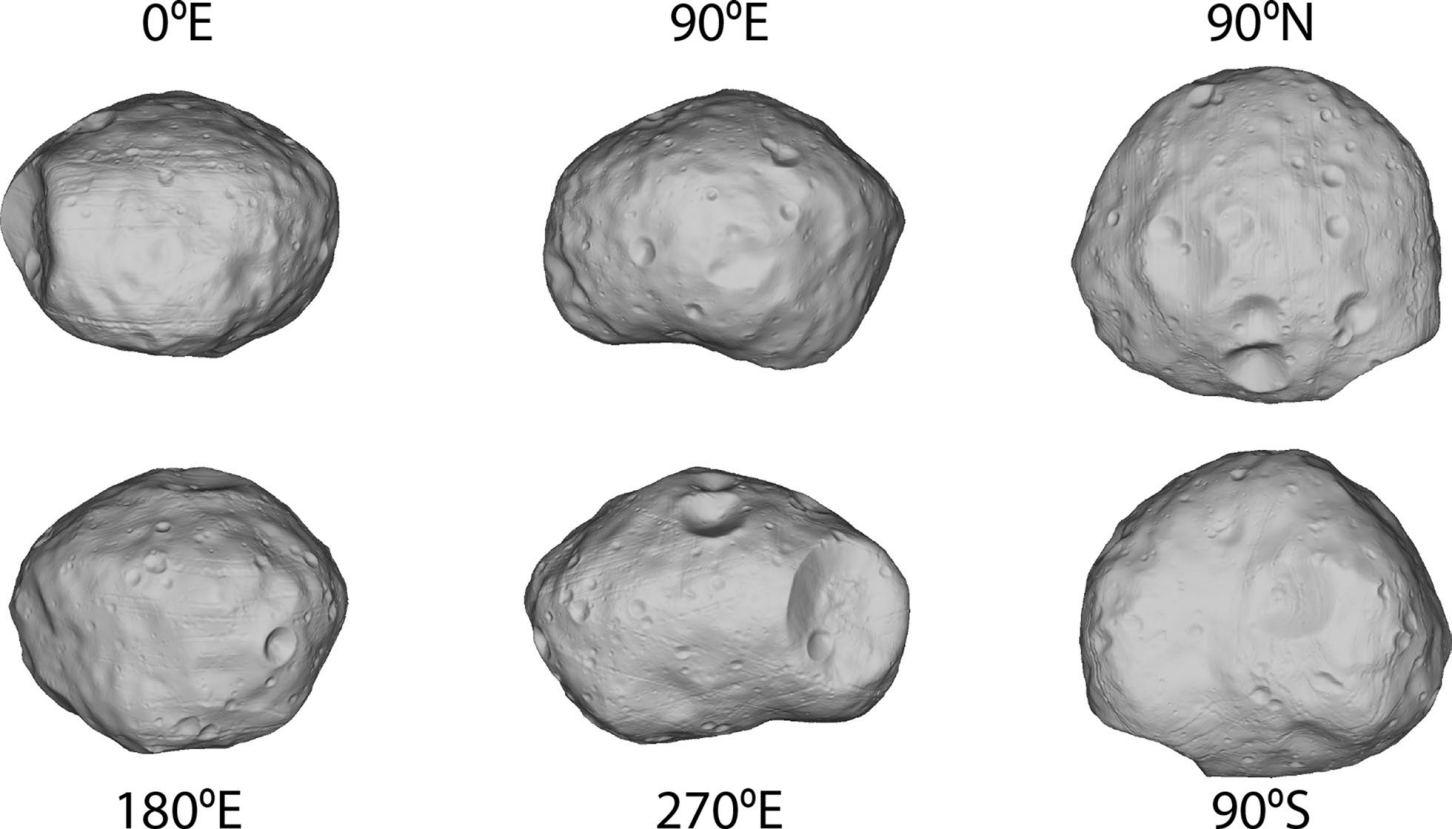
The global SPC shape model of Phobos, seen along the axes and rendered without albedo. The global model has an average resolution of 18 m per facet, over 12 million facets, and was constructed from 2382 images

Our global SPC shape model of Phobos (Fig. 8) has an average ground sample distance (GSD) of 18 m per facet and a total of over 12 million facets, and was constructed from 2382 images. Table 5 gives the physical parameters of the model. The radii of the best-fit ellipsoid are (12.95 ± 0.04) × (11.30 ± 0.04) × (9.16 ± 0.03) km. Its average radius is (11.08 ± 0.04) km, computed from an equivalent-volume sphere. The global shape model has a surface area of (1640 ± 8) km^2^ and a volume of (5695 ± 32) km^3^. Taking this volume and the value of GM = (0.7072 ± 0.0013) × 10^–3^ km^3^ s^−2^ from Pätzold et al. (2014), we calculate the bulk density to be (1861 ± 11) kg m^−3^.

**Table 5:** Comparison of Phobos global shape models

	Ernst et al. (this study)	Gaskell 2011	Willner et al. 2014^e^
Image sources	Viking Orbiter, Phobos 2, MOC, HRSC, SRC, HiRISE	Viking Orbiter, Phobos 2	Viking Orbiter, HRSC, SRC
Image number	2382	232	not available
Global model GSD (m)	18	36^a^	100
Maplet DTM GSD^b^ (m)	10	60	n/a
Average radius^c^ (km)	11.08 ± 0.04	11.12	11.12
Best-fit ellipsoid (semi-major axis)	12.95 ± 0.04 × 11.30 ± 0.04 × 9.16 ± 0.03	12.84 × 11.19 × 9.22	12.82 × 11.17 × 9.20
Volume (km^3^)	5695 ± 32	5760	5742 ± 35
Surface area (km^2^)	1640 ± 8	1650	1640
Bulk density^d^ (kg/m^3^)	1861 ± 11	1840	1846 ± 12
Comparison to MOLA	− 4.0 m median difference 20.1 m RMS	2.3 m median difference 29.4 m RMS	− 5.8 m median difference 21.7 m RMS

^a^Global model oversamples the maplet data

^b^Best maplet GSD used to construct global model

^c^Computed from an equivalent volume sphere

^d^Calculated assuming GM from Pätzold et al. (2014)

^e^Values for GSD and volume from Willner et al. 2014; others measured from the shape model or derived in the same manner as the Ernst et al (this study) and Gaskell models to enable direct comparison

We used the Gaskell (2011) Phobos model as our starting reference model. The Gaskell Phobos model was created using SPC from 224 Viking Orbiter and 8 Phobos 2 images and made from a global set of 60-m GSD maplets. This reference model was used to register a total of 216 Viking Orbiter, 7 Phobos 2, 4 MOC, 2131 HRSC + SRC, and 24 HiRISE images (Table 2). Additional images from these cameras were also registered, but did not contribute to the SPC solution due to artifacts, low resolution, etc. (see Additional file 1).

**Fig. 9:**
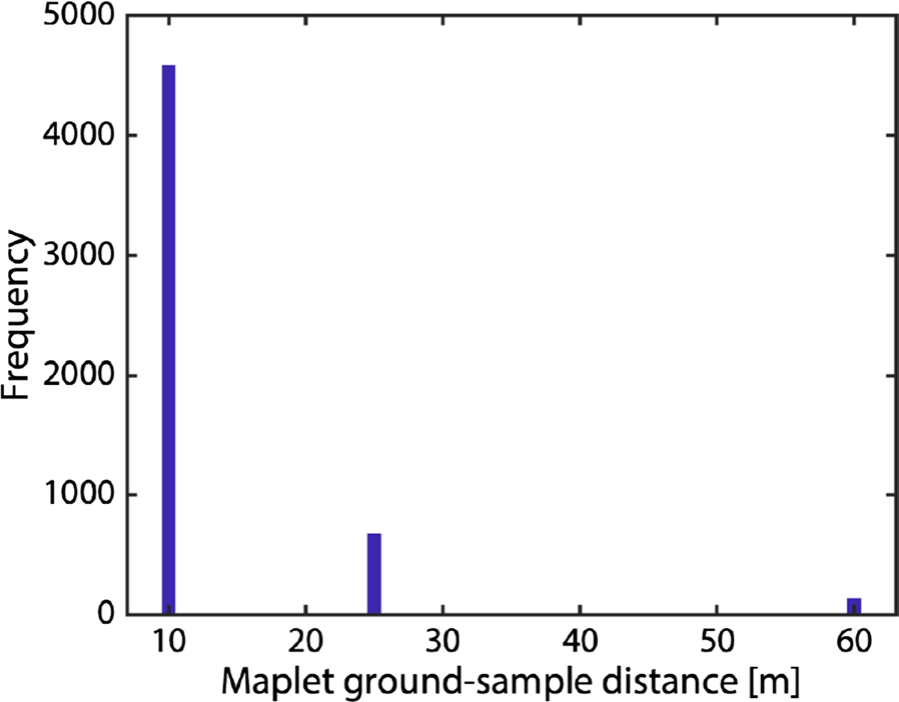
The ground sample distance of maplets making up the Phobos SPC suite. The model is globally covered by 10-, 25-, and 60-m maplets

The global, 60-m maplets were used to initiate the topography solution that incorporated the newly added images. The dataset supported global sets of maplets at successively smaller GSD: 25 m, and ultimately 10 m. Figure 9 shows a histogram of maplet ground sample distance used to derive Phobos topography; Table 2 lists the minimum, maximum, median, and mean numbers of maplets per image. On Phobos, the global imaging coverage was sufficient to allow a 60º emission angle cutoff for incorporated images.

**Fig. 10:**
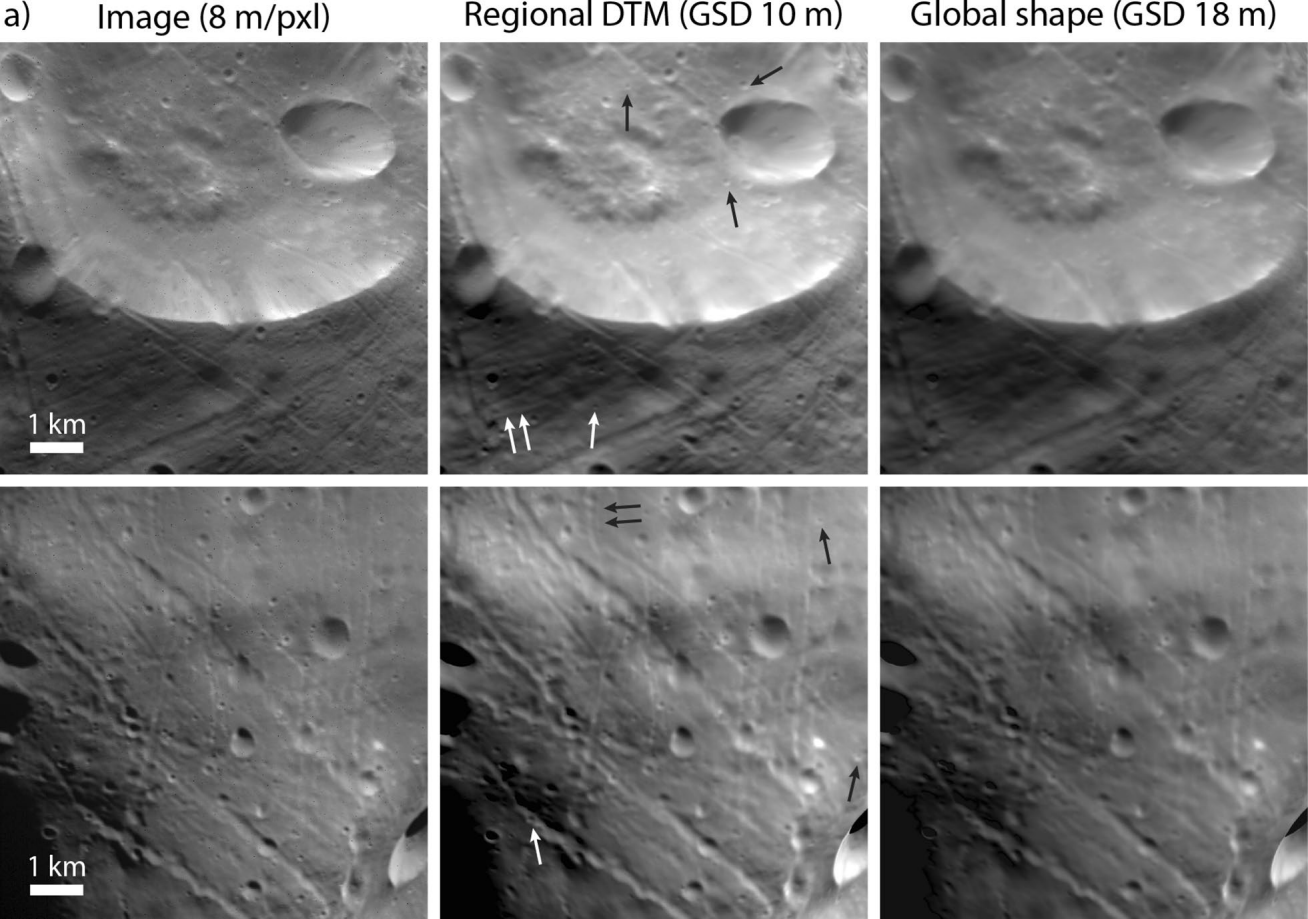
Comparison of SRC images (H9551_0005_SR2 top left; H3802_0004_SR2 bottom left) with images rendered from a regional DTM with GSD 10 m (center) and images rendered from the global shape model with GSD 18 m (right). The rendered images include the relative albedo solution derived from the SPC process. Images rendered from the higher-resolution, regional DTMs appear crisper and reveal small-scale features (~ 100 m; examples indicated by arrows) that are harder to make out in images rendered from the almost 2 × lower-resolution, global shape model

**Fig. 11:**
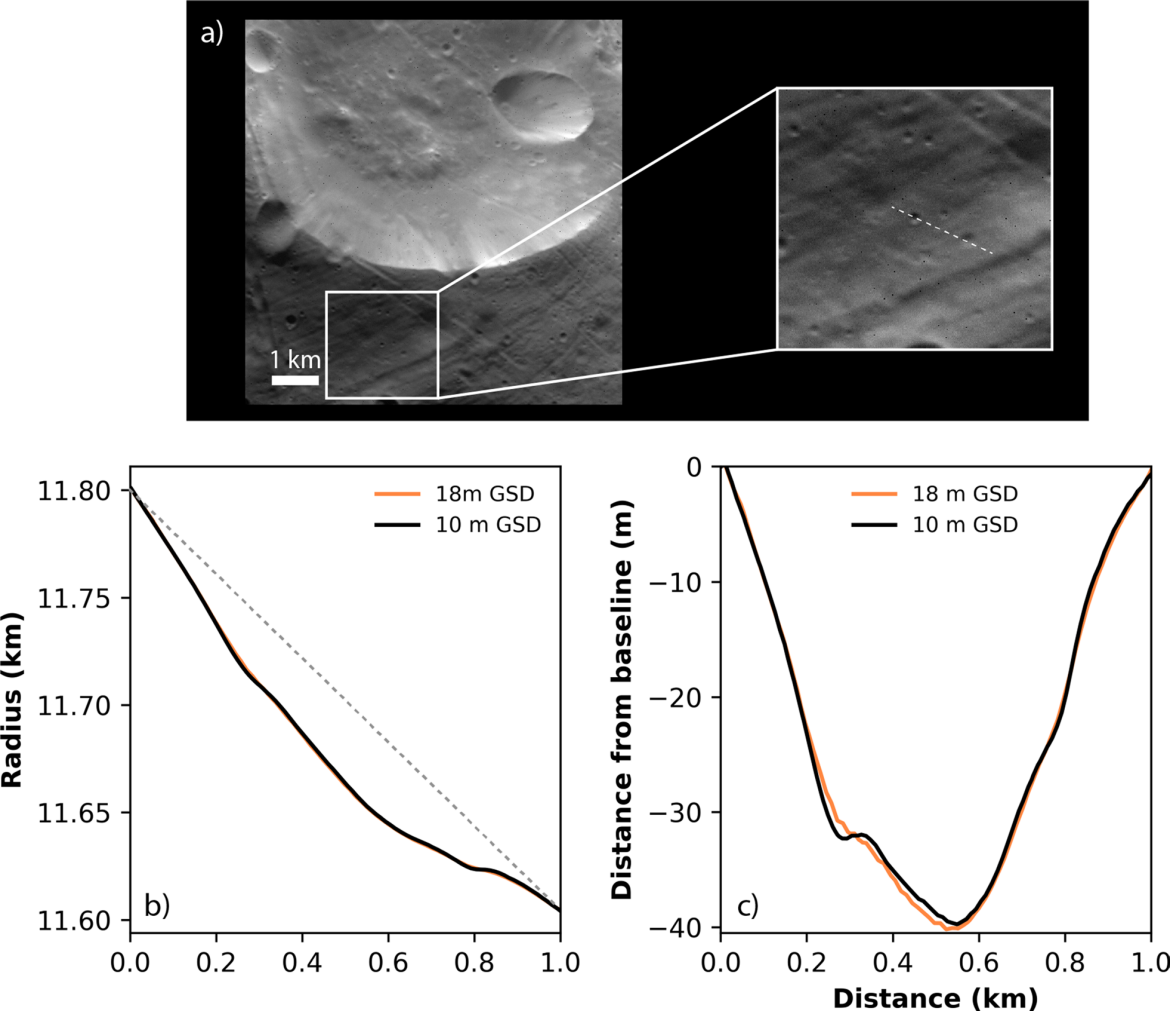
**a** Zoom-in of an SRC image (H9551_0005_SR2, see also Fig. 8) to illustrate the location of a profile taken across a small, ~ 120-m-diameter crater. **b** A comparison of topographic profiles taken across the regional DTM (10-m GSD) and the global shape model (18-m GSD). The grey dashed line shows a linear baseline between the two endpoints. **c** The same topographic profiles linearly detrended using the baseline to remove the effect of broader-scale slopes and accentuate small-scale features. The height differences between models are apparent, particularly across the small crater (located approximately 0.25 km along the profile)

The global shape model was built from a combination of the 25-m and 10-m maplets (leaving out the 60-m maplets). We limited the number of facets in the global shape model to ~ 12 million for file size practicality (~ 1 GB). The resulting 18-m GSD of the global model under-resolves the derived topography solution in the 10-m maplets by nearly a factor of two. Therefore, we also constructed a set of 54 regional DTMs with 10-m GSD (each covering ~ 100 km^2^) that globally cover Phobos’ surface to enable use of the highest-resolution derived topography while maintaining a reasonable file size. Figure 10 illustrates the added detail that can be seen when rendering an image from the global shape model versus a regional DTM with 10-m GSD. Figure 11 shows the improved height resolution of the regional DTM. The regional DTMs can be combined to cover larger areas providing a means to examine regions of interest at higher resolution (as has been done on other bodies, e.g., the artificial crater created on Ryugu (Arakawa et al. 2020); craters on Bennu (Daly et al. 2020); candidate sample site assessment on Ryugu (Tsuda et al. 2020) and Bennu (Lauretta et al. 2022)) while retaining context from the global model.

### Phobos model quality assessment

**Fig. 12:**
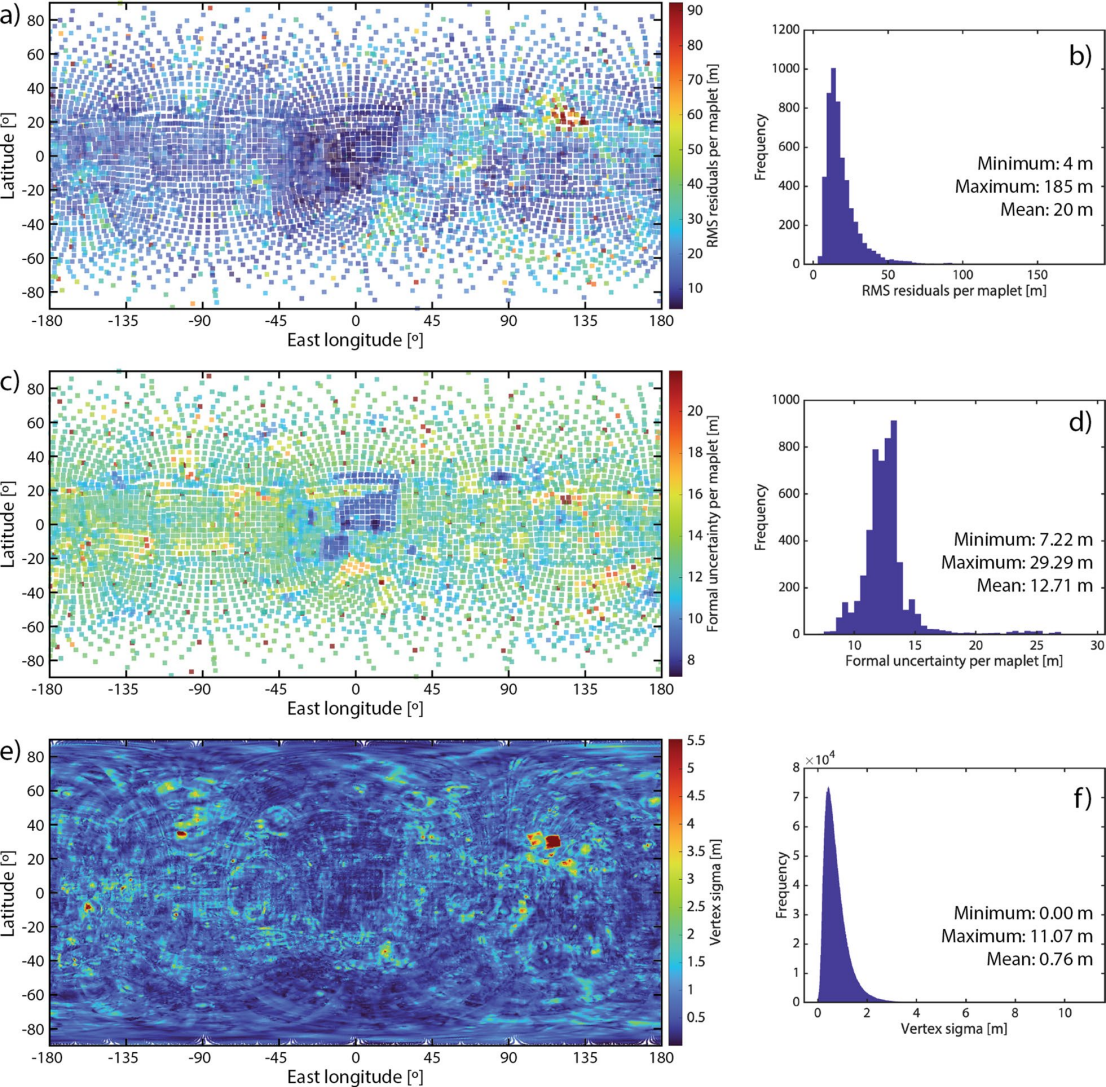
Maplet residuals (**a**, **b**), maplet formal uncertainty (**c**, **d**), and vertex sigmas (**e**, **f**) for our Phobos global SPC model. These metrics indicate a model accuracy of ± 13–20 m and a precision of ~ 4 m (5 × the mean vertex sigma). A few localized areas stand out in these metrics; these areas are associated with crater walls in areas where most images are heavily shadowed

We assessed the accuracy and precision of the Phobos shape model using the SPC-derived metrics and three comparisons to images described previously. Figure 12 illustrates the maplet residuals, maplet formal uncertainty, and vertex sigmas for our Phobos global SPC model. We find a mean maplet residual of ~ 20 m, a mean formal uncertainty of ~ 13 m, and a mean vertex sigma of < 1 m. The scaled mean vertex sigma (5 × the mean vertex sigma, an indication for regional precision uncertainties, as described in “SPC-derived metrics”) is ~ 4 m. These SPC-derived metrics indicate that the model is accurate to ± 13–20 m and has a precision of ~ 4 m. A few localized areas stand out in these metrics; these areas are located on crater walls. The most notable example near 30ºN, 118ºE is in an area with the worst SPC ideal image coverage (Fig. 5) and many of the available images contain extensive shadows. Each vertex of the global shape is produced by averaging the many maplets at that location, thereby reducing the influence of individual outlier maplets on the global shape.

**Fig. 13:**
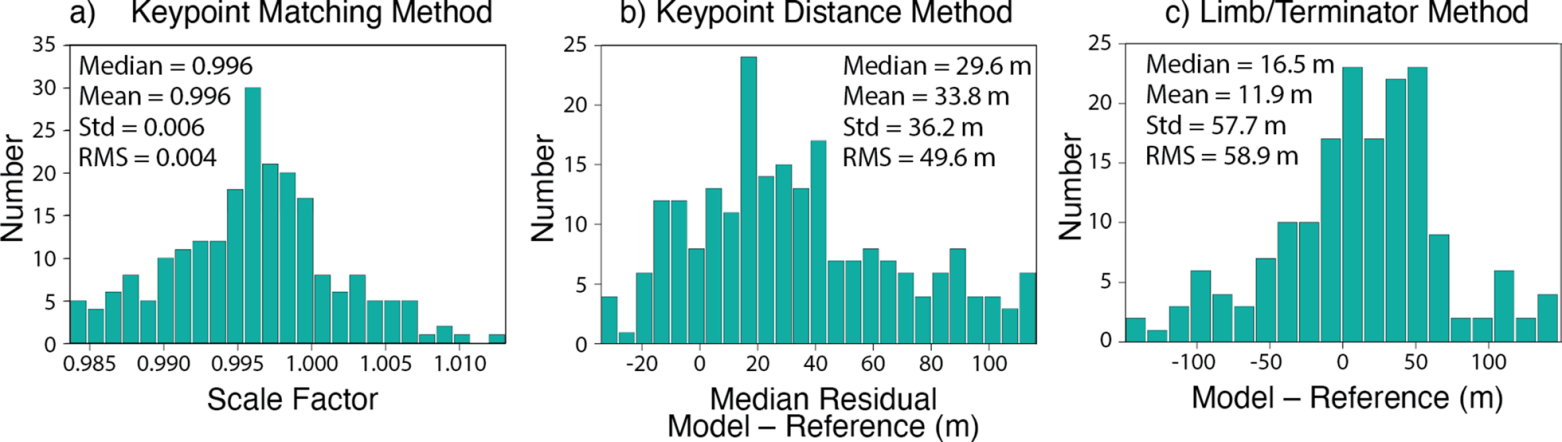
Results of the keypoint matching, keypoint distance, and limb/terminator methods applied to Phobos. **a** Histogram of the scale factor needed to match the model to the reference image based on the keypoint matching method for 222 images. Values < 1 indicate the model is larger than the images. The keypoint matching method indicates a 66-m accuracy and a + 44-m scale uncertainty for a scale factor of 0.996 (the model is slightly larger than the images). **b** Histogram of the median residual difference between the model and the reference image based on the keypoint distance method for 222 images. They keypoint distance method indicates a 50-m accuracy and a + 30-m scale uncertainty. **c** Histogram of the difference between the model (rendered image) and the reference image based on the limb/terminator method for 173 images. The limb/terminator comparison indicates a 59-m accuracy and a + 17-m scale uncertainty (again the model is larger than the images)

Figure 13 shows the results of the keypoint matching, keypoint distance, and limb/terminator methods applied to our Phobos model. The keypoint matching and keypoint distance methods were performed on 222 Viking Orbiter and SRC images. The keypoint matching analysis revealed a 66-m accuracy and a + 44-m scale uncertainty (the model is larger than the images) for a scale factor of 0.996. The keypoint distance analysis indicates a 50-m accuracy and a + 30-m scale uncertainty. The limb/terminator method was performed on 173 Viking Orbiter and SRC images where the field of view contained all of Phobos and (particularly for SRC) the limb was crisply resolved. The limb/terminator method indicated a 59-m accuracy and a + 17-m scale uncertainty (again the model is larger than the images).

Table 6 summarizes the various accuracy estimates for the Phobos model. The model accuracy estimate based solely on the input images ranged from 20–100 m. The model accuracy estimates of the 18-m GSD model from the SPC metrics, image comparisons, and MOLA comparison (see “Comparison with MOLA”) range from 13 to 66 m, with a median value of 36 m. We take this median value to represent the accuracy in the global Phobos model, and apply that as the error for the model average radius. The other errors reported are propagated from this value. The model accuracy is 1.8 × the 20-m representative mean pixel scale at which Phobos is globally covered with images satisfying the sufficient SPC criteria (Fig. 5). The model precision estimate based solely on the input images ranged from 5–60 m. We have only one estimate of model precision, the scaled mean vertex sigma of 4 m. This model precision is 0.4 × the 10-m representative best pixel scale. These accuracy and precision estimates are both consistent with the better values predicted by the rules of thumb, which is consistent with the fact that the images meet the sufficient SPC imaging criteria (Fig. 5). The keypoint and limb/terminator analyses indicate our Phobos shape model is 17–44 m too large, which amounts to between ~ 0.2 and 0.4% the average radius of Phobos.

**Table 6:** Accuracy and precision comparisons

	Phobos accuracy (m)	Deimos accuracy (m)
Prediction from input images		
Rule of thumb	20–100	75–375
SPC-derived metrics		
Mean maplet residual	20	8
Maplet formal uncertainty	13	22
Image comparisons		
Keypoint matching	66	188
Keypoint distance	50	131
Limb/terminator	59	65
Comparison to MOLA	21	n/a
Median of Accuracy Metrics	36	65

	Phobos precision (m)	Deimos precision (m)
Prediction from input images		
Rule of thumb	5–60	25–300
SPC-derived metrics		
Scaled vertex sigma	4	9

### Comparison with other Phobos shape models

Shape models of Phobos have been derived using a variety of methods since the first close-up images of the moon were acquired by Mariner 9. A summary of most of these models can be found in Willner et al. (2014). Here, we compare our new global Phobos shape model with two previously derived, high-resolution global models: (1) the Gaskell (2011) model derived from Viking Orbiter and Phobos 2 images (which served as our starting reference model); (2) the Willner et al. (2014) model derived from HRSC, SRC, and Viking Orbiter images. Table 5 compares the three Phobos shape models. Our values for average radius, best-fit ellipsoid, volume, surface area, and bulk density are all consistent with the Gaskell (2011) and Willner et al. (2014) models when derived in the same manner.

**Fig. 14:**
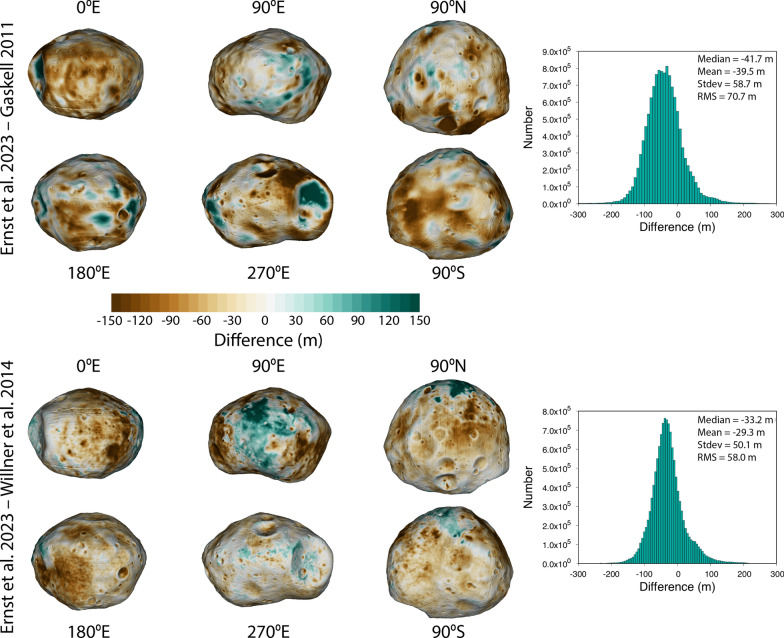
Difference between the Ernst et al. (this study) Phobos global shape model and those of Gaskell 2011 (top) and Willner et al. 2014 (bottom). All three models are in generally good agreement with one another. The new model is nearly the same as the previous models in some areas (white in the color scale), larger in some areas (green in the color scale), and smaller in some areas (brown in the color scale). The new model on average is slightly smaller (30–40 m, corresponding to ~ 0.3–0.4% of the mean body radius; ~ 1% smaller in volume) than the two previous models

We calculated the optimal rigid transformations (rotation and translation) to minimize the RMS difference between our model and the Gaskell (2011) and Willner et al. (2014) global shape models and applied these transformations to align all three models. Figure 14 illustrates the shape differences calculated across the body. All three models are in generally good agreement with one another. We found a median difference of ~ -42 m and RMS difference of ~ 71 m between the Ernst et al (this study) and Gaskell models. The most obvious differences spatially can be seen within and around Stickney crater (270ºE view). We find a median difference of ~ -33 m and an RMS difference of ~ 58 m between the Ernst et al (this study) and the Willner et al. models. The largest affected regions are around 90ºE and within most craters (which are deeper in the new model, likely because they are better resolved). The new model on average is slightly smaller (30–40 m, corresponding to ~ 0.3–0.4% of the mean body radius; ~ 1% smaller in volume) than the two previous models.

**Fig. 15:**
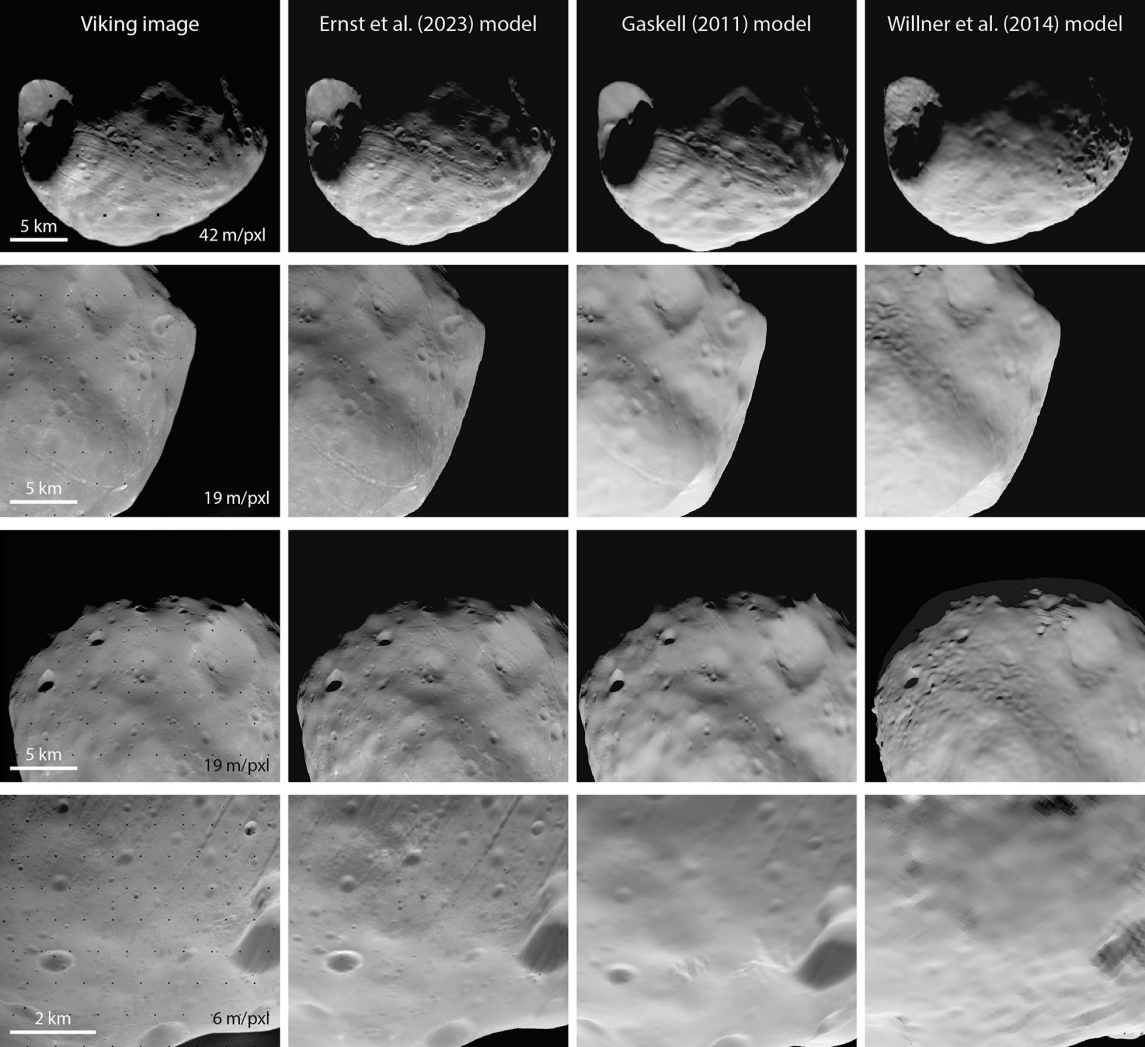
Comparison of Phobos Viking Orbiter VIS images (top to bottom: f357a64, f315a12, f315a11, f246a08) with images rendered from the global shape models of Ernst et al. (this study), Gaskell (2011), and Willner et al. (2014). The Ernst et al (this study) model rendered images incorporate the SPC-derived relative albedo solution. Image pixel scale is indicated in the left column. The bulk shape of Phobos is similar for all three shape models. Small-scale details can be resolved in the Ernst et al (this study) model that are not resolvable in the previous models. Such features are particularly noticeable in the bottom row

**Fig. 16:**
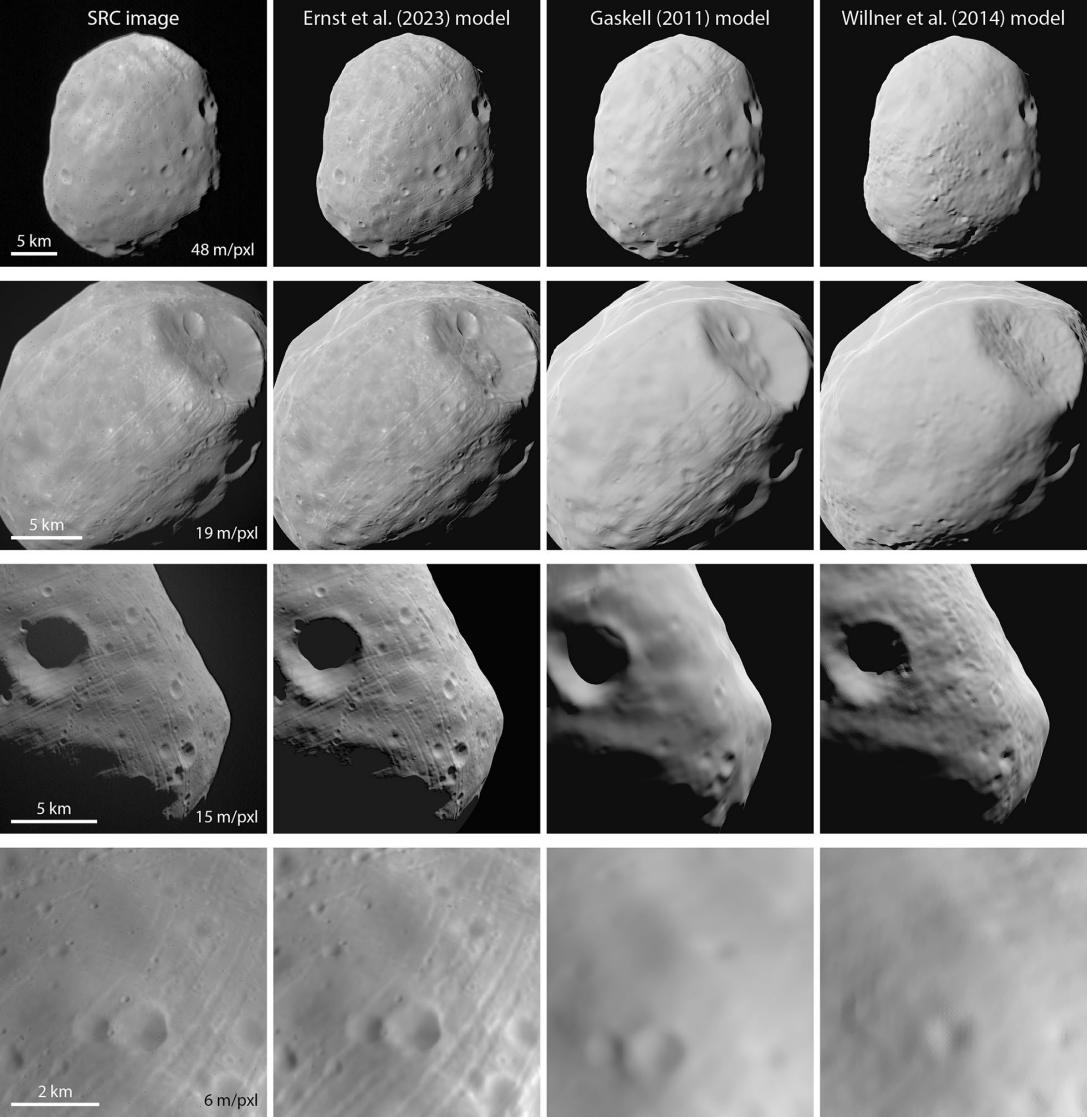
Comparison of Phobos Mars Express SRC images (top to bottom: H2601_0006_SR2, H4447_0005SR2, HD683_0004SR2, H4847_0005SR2) with images rendered from the global shape models of Ernst et al. (this study), Gaskell (2011), and Willner et al. (2014). The Ernst et al (this study) model rendered images incorporate the SPC-derived relative albedo solution. Image pixel scale is indicated in the left column. The bulk shape of Phobos is similar for all three shape models. Small-scale details can be resolved in the Ernst et al (this study) model that are not resolvable in the previous models. Such features are particularly noticeable in the bottom two rows

Figures 15 and 16 compare Viking orbiter images and SRC images, respectively, with images rendered from the Ernst et al. (this study), Gaskell (2011), and the Willner et al. (2014) global shape models. The bulk shapes are similar among the three models, as is expected from the quantitative comparisons shown in Fig. 14. The Ernst et al (this study) model resolves small-scale details (~ 100 m) that are visible in the images but not in the other models. The higher-resolution images in the bottom row of Figs. 15 and 16 highlight the additional detail in the Ernst et al (this study) model.

### Comparison with MOLA

**Fig. 17:**
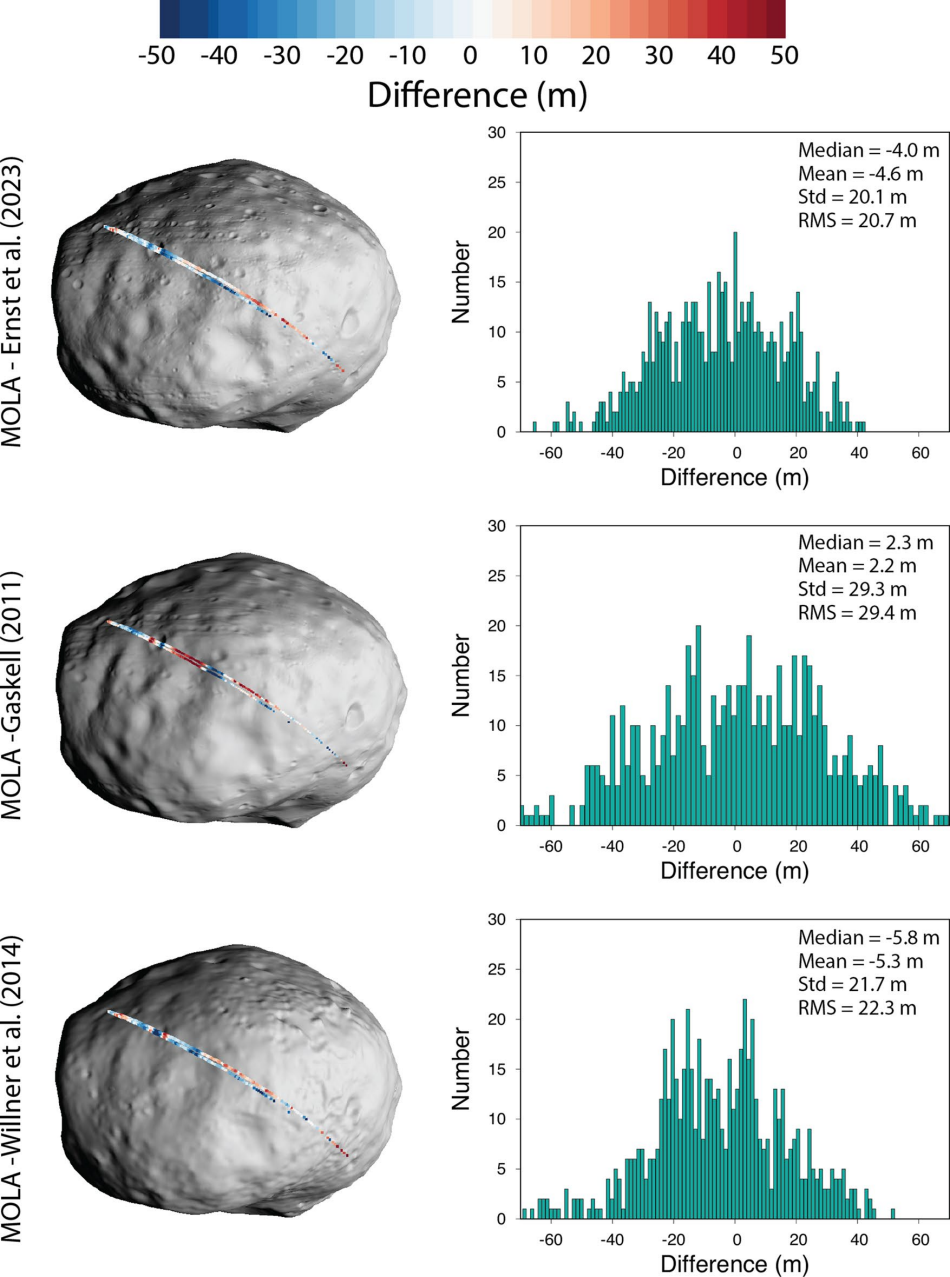
Comparison between MOLA tracks and Phobos global shape models. MOLA tracks were translated and rotated as a unit to best match each global shape model. The difference between the two models is shown along the MOLA track superposed onto the shape model at left (views centered on 0ºN, 20ºE). A histogram of the differences (MOLA-shape) is shown at right. The RMS difference gives an indication of the uncertainty in model hemispherical accuracy

Two altimetry tracks were taken during one Phobos flyby by the MGS Mars Orbital Laser Altimeter (MOLA). Major errors in the groundtrack location were corrected by adjusting the relative positions of the MGS spacecraft and low-resolution shape model of Phobos (Thomas 1993; Thomas et al. 2000) and recomputing the locations of the measurements (Banerdt and Neumann 1999), yielding a RMS difference of ~ 67 m. But the correction was limited by the quality of the images and shape model at that time, which had a GSD of ~ 370 m. We rotated and translated the MOLA tracks as a unit to our shape model to provide a best fit to the data, and calculated the difference between the MOLA tracks and the shape model (Fig. 17, Table 5). The RMS difference gives an indication of the uncertainty in model hemispherical accuracy, since the MOLA data span a large fraction of the body and have a relatively large footprint (130–200 m Banerdt and Neumann 1999). For the Ernst et al. (this study) model, we find an RMS difference of 20.1 m; for the Gaskell model, we find an RMS difference of 29.3 m; for the Willner et al. model, we find an RMS difference of 21.7 m. All three models are in good agreement with the MOLA data and thus capture the broad hemispherical shape of Phobos at the 100–200-m scale (~ MOLA footprint scale).

### Deimos shape model

**Fig. 18:**
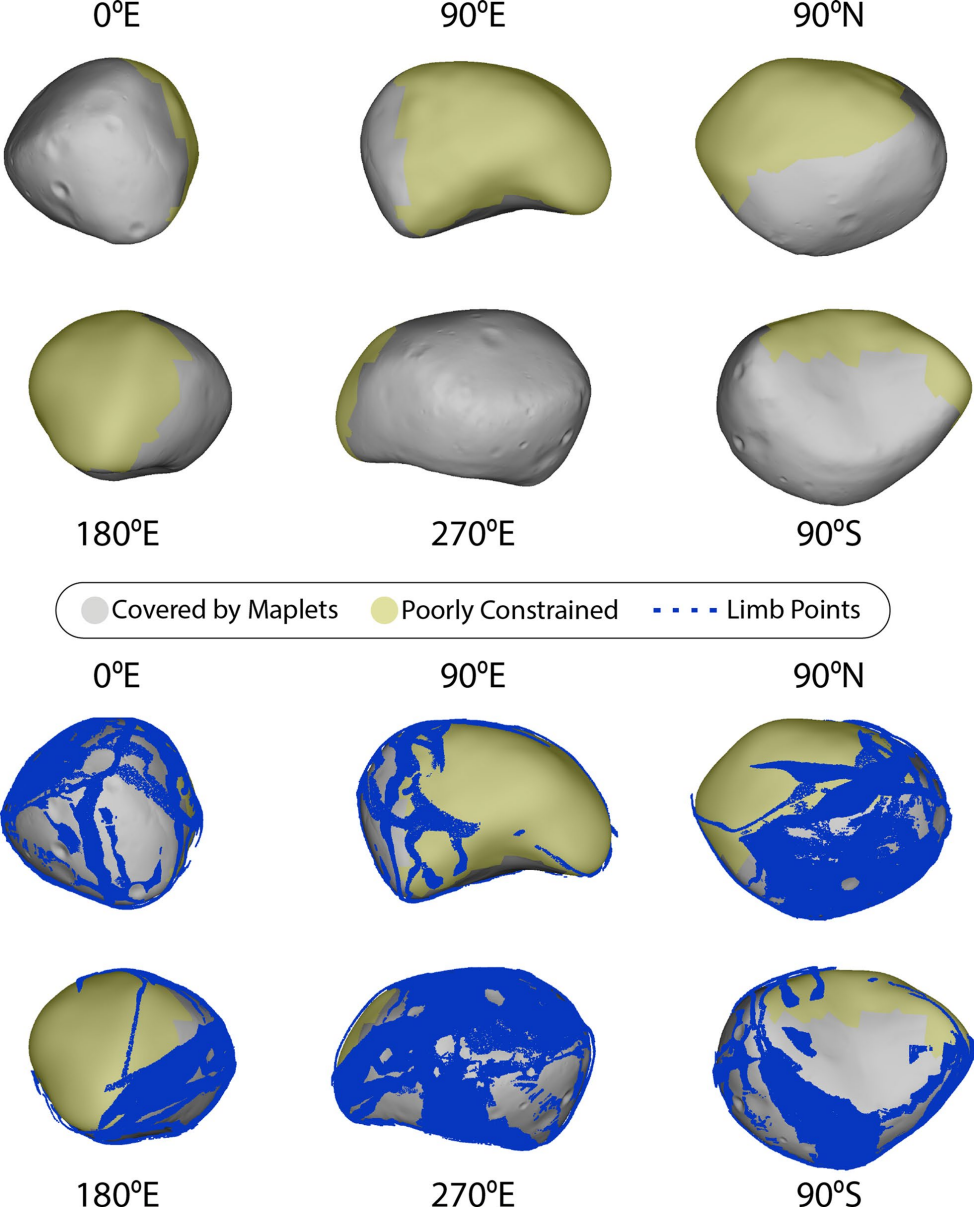
(Top) The global SPC shape model of Deimos, seen along the axes and rendered without albedo. The global model has an average resolution of 20 m per facet, a total of over 3 million facets, and was constructed from 332 images. Areas shaded yellow are constrained only by limbs. (bottom) The global SPC shape model of Deimos including the limb points used to constrain the model. The limb points have been radially offset slightly above the body to aid visibility

Our global shape model of Deimos (Fig. 18) is the first to have been created using SPC, and the first to resolve geologic features. The model has an average ground sample distance (GSD) of 20 m per facet and a total of over 3 million facets, and was constructed from 332 images. Table 7 gives the physical parameters of the model. The radii of the best-fit ellipsoid are (8.04 ± 0.08) × (5.89 ± 0.06) × (5.11 ± 0.05) km. Its average radius is (6.27 ± 0.07) km, computed from an equivalent-volume sphere. The global shape model has a surface area of (522 ± 8) km^2^ and a volume of (1033 ± 19) km^3^. Taking this volume and the value of GM = (0.101 ± 0.003) × 10^–3^ km^3^ s^−2^ from Jacobson (2010), we calculate the bulk density to be (1465 ± 51) kg m^−3^.

**Table 7:** Comparison of Deimos global shape models

	Ernst et al. (this study)	Thomas 1993
Image sources	Viking Orbiter, SRC, HiRISE	Viking Orbiter
Image number	332	12
Global model GSD^a^ (m)	20	600 (@ equator; 5º grid)
Maplet DTM GSD^b^ (m)	25	n/a
Average radius^c^ (km)	6.27 ± 0.07	6.24 ± 0.25
Best-fit ellipsoid (semi-major axis)	8.04 ± 0.08 × 5.89 ± 0.06 × 5.11 ± 0.05	7.8 × 6.0 × 5.1
Volume (km^3^)	1033 ± 19	1017 ± 130
Surface area (km^2^)	522 ± 8	519 ± 45
Bulk density^d^ (kg/m^3^)	1465 ± 51	1488 ± 195

^a^Global model oversamples the maplet data

^b^Best maplet GSD used to construct global model

^c^Computed from an equivalent volume sphere

^d^Calculated assuming GM from Jacobson (2010)

We used the Thomas (1993) Deimos model as our starting reference model. The Thomas (1993) Deimos model was created from limb- and control points from only 12 Viking Orbiter images and has facets with ~ 600 m ground sample distance at the equator (5º grid). This reference model was used to register 95 Viking Orbiter, 1 MOC, 227 SRC, and 9 HiRISE images (Table 3). Additional images were registered, although did not contribute to the SPC solution (see Additional File 2).

**Fig. 19:**
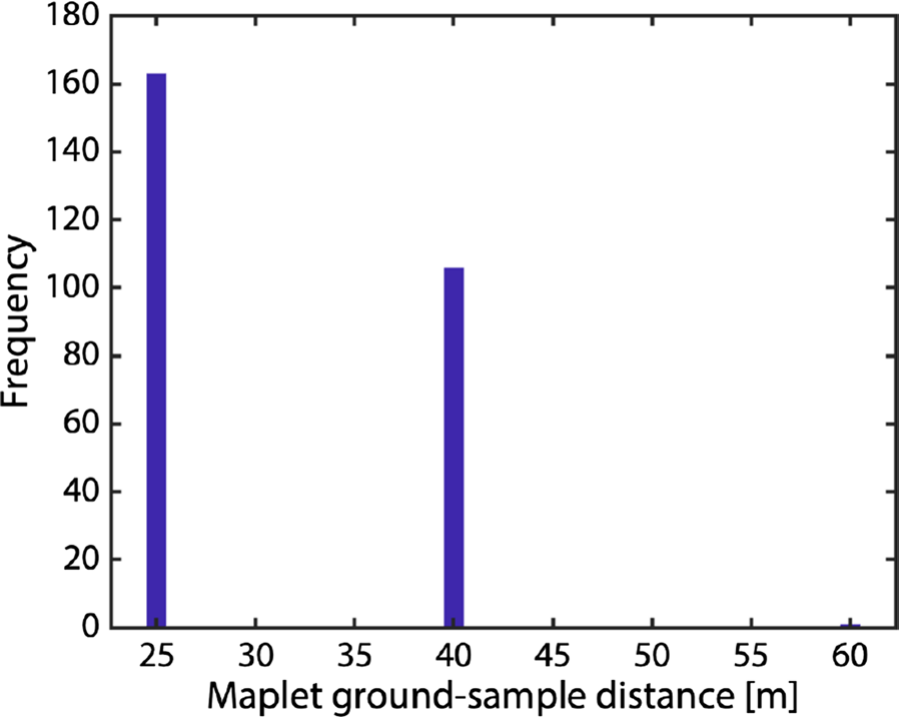
The ground sample distance of maplets making up the Deimos SPC suite. The sub-Mars hemisphere covered by 25- and 40-m maplets. One map at 60-m GSD was created to extend coverage near the south pole

The reference model was tiled with an initial set of maplets with 40-m GSD. Sufficient images were available to derive topography for maplets covering the sub-Mars hemisphere. The dataset supported a hemispherical set of maplets with 25-m GSD. One map at 60-m GSD was created to extend coverage near the south pole. Figure 19 shows a histogram of maplet ground sample distance used to derive Deimos topography; Table 3 lists the minimum, maximum, median, and mean numbers of maplets per image. On Deimos, the more limited, hemispherical imaging coverage meant that some maplets incorporated images with emission angles up to 70º.

A comparison between the Thomas (1993) model and the ~ 28 × larger number of images used in this effort revealed differences along some limbs. Therefore, we generated a limb-based model using the SPC program called limber using 131 images as inputs. Limber identifies limb points in individual images and produces a point cloud in body-fixed coordinates (for technical information about limber see Palmer et al. 2022). This point cloud (Fig. 18) was combined with a point cloud generated from the Thomas (1993) model to produce our limb-based model. This limb-based SPC model provided a starting point for the construction of the global shape model, which was derived from the entire maplet ensemble. Because one hemisphere lacks maplets, its shape is made up of an interpolated version of the reference limb-based SPC model (Fig. 18). We did not produce regional DTMs, as the global model GSD slightly oversamples the best resolution maplets.

### Deimos model quality assessment

**Fig. 20:**
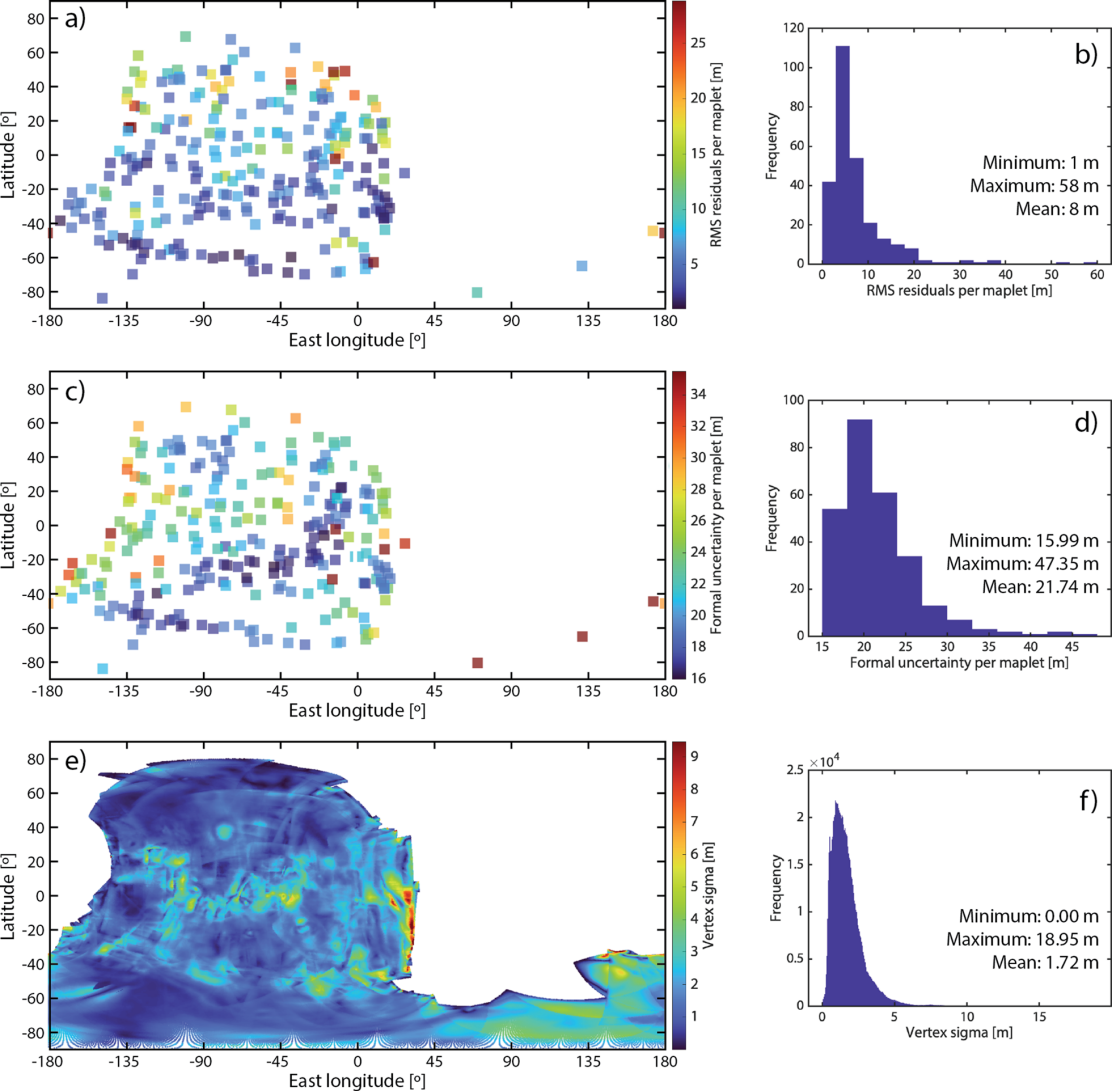
Maplet residuals (**a**, **b**), maplet formal uncertainty (**c**, **d**), and vertex sigmas (**e**, **f**) for our Deimos global SPC model. These metrics indicate a model accuracy of ± 8–22 m. The vertex sigma for Deimos is likely not a good indicator of precision, due to limited satisfaction of the SPC imaging criteria. High vertex sigma values are concentrated at the edges of maplet coverage, where the maps do not agree well with the poorly constrained shape

We assessed the accuracy and precision of the Deimos shape model using the SPC-derived metrics and three comparisons to images described previously. Figure 20 illustrates the maplet residuals, maplet formal uncertainty, and vertex sigmas for our Deimos global SPC model. We find a mean maplet residual of ~ 8 m, a mean formal uncertainty of ~ 22 m, and a mean vertex sigma of ~ 1.7 m. The scaled mean vertex sigma is ~ 9 m. High vertex sigma values are concentrated at the edges of maplet coverage, where the maps do not agree well with the poorly constrained shape. These SPC-derived metrics indicate that in areas where there are maplets, the model is accurate to ± 8–22 m and has a precision of ~ 9 m (see below, this is likely an unreliable precision estimate).

**Fig. 21:**
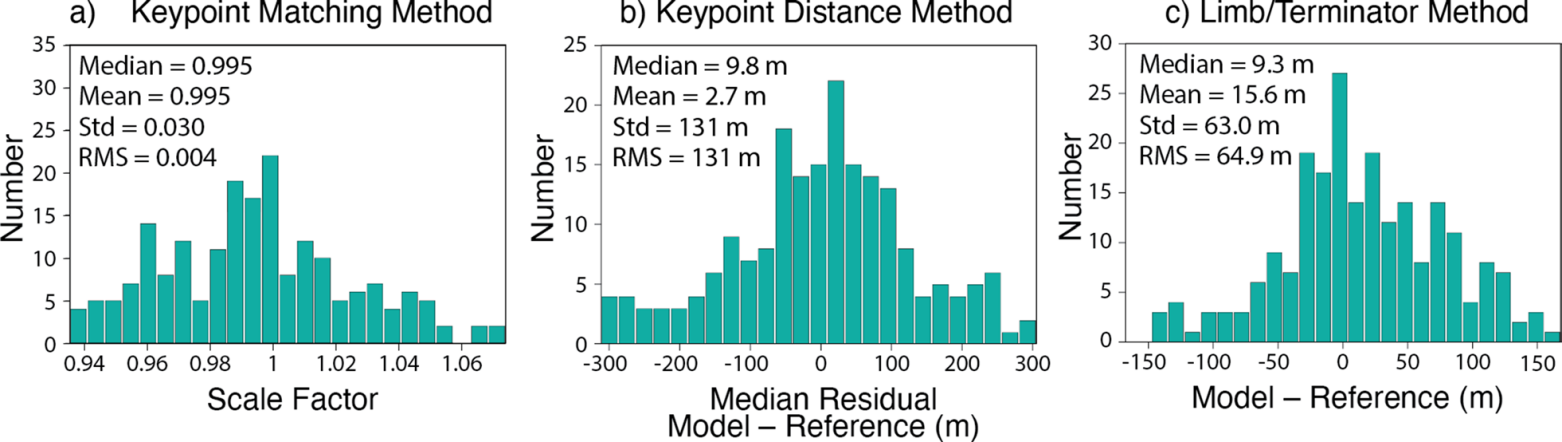
Results of the keypoint matching, keypoint distance, and limb/terminator methods applied to Deimos. **a** Histogram of the scale factor needed to match the model to the reference image based on the keypoint matching method for 125 images. Values < 1 indicate the model is larger than the images. The keypoint matching method indicates a 188-m accuracy and a + 31-m scale uncertainty for a scale factor of 0.995 (the model is slightly larger than the images). **b** Histogram of the median residual difference between the model and the reference image based on the keypoint distance method for 125 images. They keypoint distance method indicates a 131-m accuracy and a + 10-m scale uncertainty. **c** Histogram of the difference between the model (rendered image) and the reference image based on the limb/terminator method for 219 images. The limb/terminator comparison indicates a 65-m accuracy uncertainty and a + 9-m scale uncertainty (again the model is larger than the images)

Figure 21 shows the results of the keypoint matching, keypoint distance, and limb/terminator methods applied to our Deimos model. The keypoint matching and keypoint distance methods were performed on 125 Viking Orbiter and SRC images. The keypoint analyses were more difficult for Phobos than for Deimos due to the more limited dataset and fewer surface features to act as keypoints. The keypoint matching analysis revealed a 188-m accuracy and a + 31-m scale uncertainty (again the model is slightly larger than the images) for a scale factor of 0.995. The keypoint distance analysis indicates a 131-m accuracy and a + 10-m scale uncertainty. The limb/terminator comparison was performed on 219 Viking Orbiter and SRC images where the field of view contained all of Deimos and (particularly for SRC) the limb was well resolved. The limb/terminator comparison indicated a 65-m accuracy and a + 9-m scale uncertainty (the model is larger than the images).

Table 6 summarizes the various accuracy estimates for the Deimos model. The model accuracy estimate based solely on the input images ranged from 75 to 375 m. The model accuracy estimates of the 20-m GSD model from the SPC metrics and image comparisons range from 8 to 188 m, with a median value of 65 m. We take this median value to represent the accuracy in the SPC-derived hemisphere of the global Deimos model, and apply that as the error for the model average radius. The other errors reported are propagated from this value. The model accuracy is 0.9 × the 75-m pixel scale at which Deimos is hemispherically covered with images satisfying the sufficient SPC criteria (Fig. 6), which is consistent with the better values predicted by the rules of thumb. We have only one estimate of model precision, the scaled mean vertex sigma of 9 m. This model precision is ~ 0.2 × the 50-m representative best pixel scale, which is better than the values predicted by the rules of thumb. Given this overperformance compared to our rules of thumb, it is possible that integrating the pushbroom images (particularly HiRISE) into the ideal SPC imaging assessment would show that the representative best pixel scale is smaller (~ 20 m) than that estimated earlier (see “Quality of image sets relative to ideal SPC imaging criteria”). The keypoint and limb/terminator analyses indicate our Deimos shape model is 9–31 m too large, which amounts to between 0.1% and 0.5% the average radius of Deimos.

### Comparison with other Deimos shape models

Few global shape models of Deimos exist. Here, we compare our new global Deimos shape model with the Thomas (1993) model, which as described above was derived using limb- and control points from only a handful of Viking images. Table 7 provides a comparison between the two Deimos shape models. Our values for average radius, best-fit ellipsoid, volume, surface area, and bulk density are all consistent with the Thomas (1993) model when derived in the same manner.

**Fig. 22:**
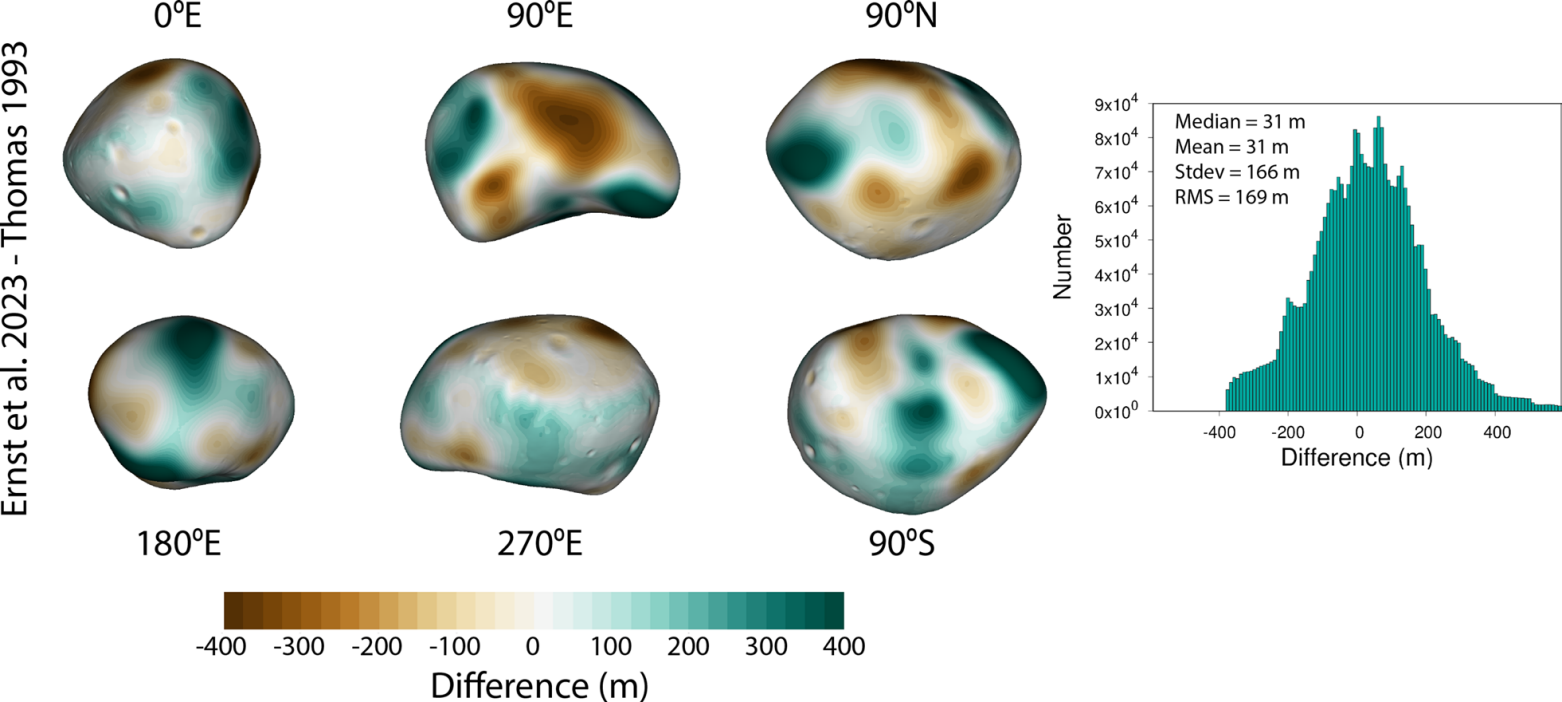
Difference between the Ernst et al. (this study) and Thomas 1993 Deimos global shape models. The two models are in generally good agreement with one another. The new model is nearly the same as the previous models in some areas (white in the color scale), larger in some areas (green in the color scale), and smaller in some areas (brown in the color scale). The new model on average is slightly larger (~ 30 m, corresponding to ~ 0.5% of the mean body radius; ~ 2% larger in volume) than the Thomas model. Much of the volume increase is concentrated in the area between ~ 15–30ºS and ~ 140–190ºE where additional limb vectors have built out the shape

We calculated the optimal rigid transformations (rotation and translation) to minimize the RMS difference between our model and the Thomas (1993) global shape model and applied these transformations to align the models. Figure 22 illustrates the shape differences calculated across the body. The models are in generally good agreement with one another. We found a median difference of ~ 31 m and RMS difference of 169 m between the two. The broadest areas of difference can be seen in the 90ºE and 180ºE views; these are regions where there are no SPC maplets and the shape is instead determined by limbs only. Much of the volume increase is concentrated in the area between ~ 15–30ºS and ~ 140–190ºE where additional limb vectors have built out the shape. The new model on average is slightly larger (~ 30 m, corresponding to ~ 0.5% of the mean body radius; ~ 2% larger in volume) than the Thomas model.

**Fig. 23:**
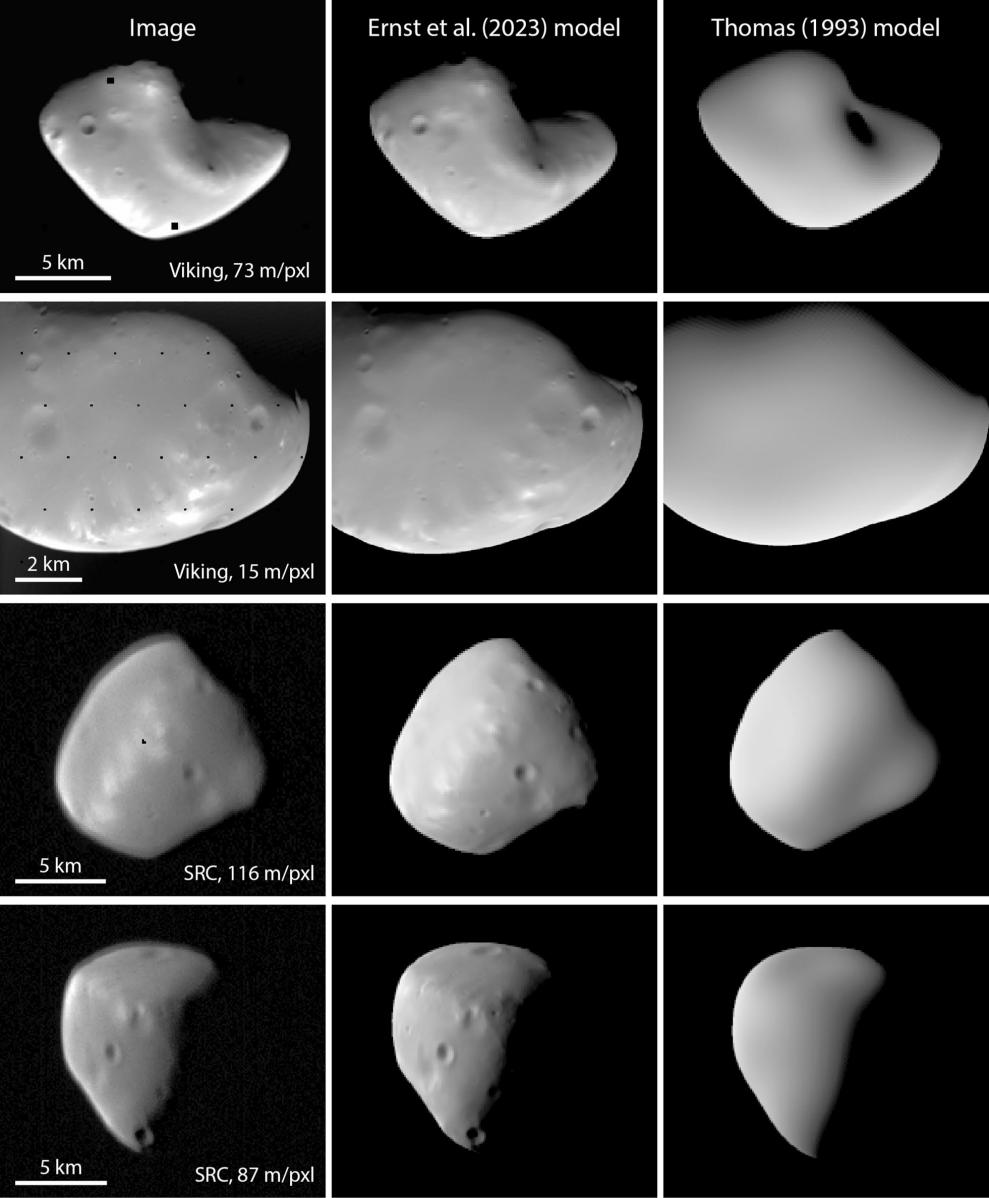
Comparison of Deimos Viking Orbiter VIS and Mars Express SRC images (top to bottom: f355b53, f428b60, H8263_0004SR2, H9253_0005SR2) with images rendered from the global shape models of Ernst et al. (this study) and Thomas (1993). The Ernst et al (this study) model rendered images incorporate the SPC-derived relative albedo solution. Image pixel scale is indicated in the left column. Although the general shapes of both models are similar, surface features can be resolved in the Ernst et al (this study) model. Note the effect of the astigmatism toward the top of the body in the SRC images

Figure 23 compares Viking Orbiter and SRC images with images rendered from the Ernst et al. (this study) and Thomas (1993) global shape models. The bulk shapes of the two models are similar, as is expected from the quantitative comparisons shown in Fig. 22. Notably, the Ernst et al. model resolves surface features, such as craters, that the Thomas model does not. The ghosting/blurring of SRC images is obvious in the lower two panels.

### Geophysical maps

We derived gravitational acceleration and slope maps (hereafter called geophysical maps) for the new global shape models of Phobos and Deimos. Slope is defined relative to gravitational acceleration. The algorithm of Werner and Scheeres (1997) was used to compute the gravitational acceleration using knowledge of the moons’ rotations, and assuming a uniform density (see Tables 5 and 6). The calculations account for the presence of Mars and assume a distance equal to the semi-major axis of each moon’s orbit. Ballouz et al. (2019) showed that slopes on Phobos can vary by up to 2º over the course of one orbit due to its eccentricity.

**Fig. 24:**
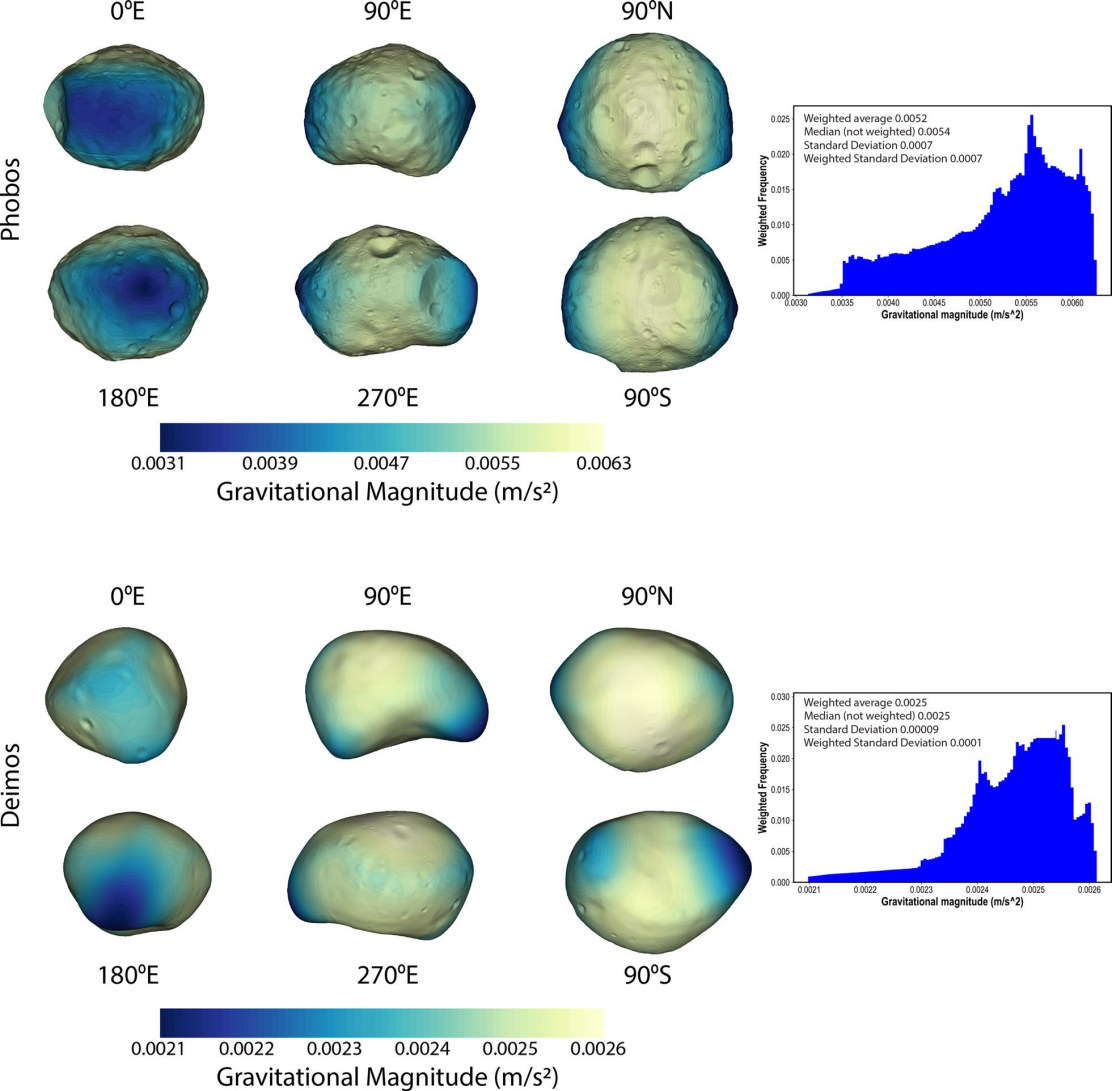
Gravitational magnitude across the surfaces of the new Phobos (top) and Deimos (bottom) SPC models. The calculations account for the presence of Mars at a distance equal to the mean semi-major axis of each moon’s orbit

Figure 24 shows the gravitational acceleration across the global shapes of Phobos and Deimos. The higher-resolution topography of our new models does not significantly affect the magnitude of gravitational acceleration versus previous calculations (e.g., Thomas 1993; Wang and Wu 2020).

**Fig. 25:**
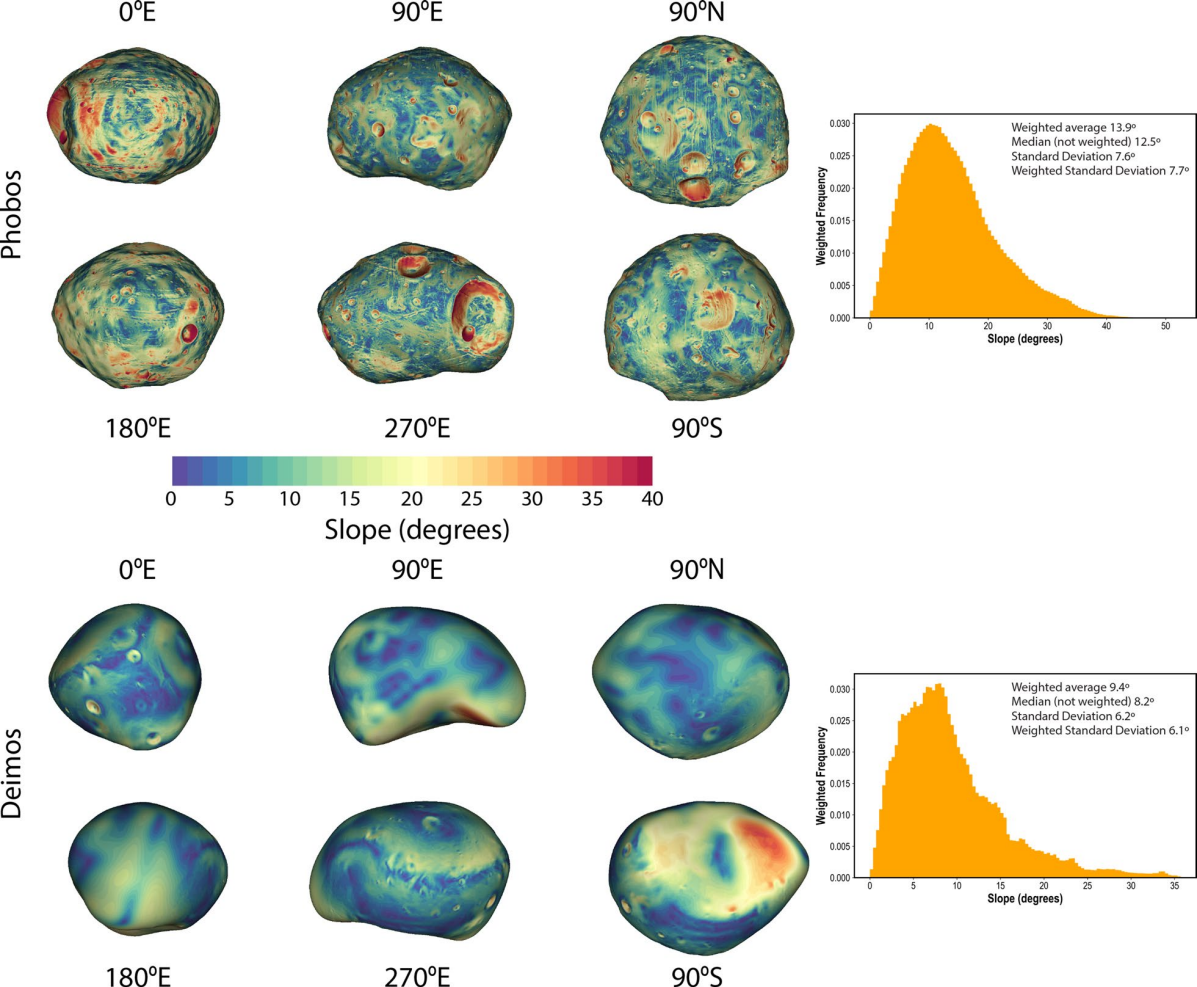
Distribution of surface slopes on the new Phobos (top) and Deimos (bottom) SPC models. The calculations account for the presence of Mars at a distance equal to the mean semi-major axis of each moon’s orbit. High slopes on Phobos are located primarily within the walls of craters. High slopes on Deimos are primarily located in the saddle (90ºS view), which accounts for the tail of histogram at slopes > 20º. The higher-resolution topography reveals more locations with steep slopes relative to previous studies

Figure 25 shows the distribution of slopes across Phobos and Deimos. High slopes on Phobos are located primarily on crater walls. High slopes on Deimos are primarily located in the saddle (90ºS view), which accounts for the tail of histogram at slopes > 20º. The higher-resolution topography reveals more locations with steep slopes relative to previous studies (e.g., Thomas 1993; Willner et al. 2014; Ballouz et al. 2019; Wang and Wu 2020) but otherwise look similar to these previous efforts.

### Data products and availability

Table 8 lists the data products produced through this effort. The main repository for these data products is the public version of the Small Body Mapping Tool (SBMT), which can be found at https://sbmt.jhuapl.edu. The SBMT is an interactive, 3D visualization and data search and analysis tool developed at the Johns Hopkins University Applied Physics Laboratory to search for, map, and analyze features on irregularly shaped solar system bodies (Ernst et al. 2018). The SBMT can be used not only to access, visualize, and download the data products listed in Table 8, but also to browse image preview galleries, generate image backplanes, map features, generate custom 10-m GSD DTMs from the set of high-resolution regional DTMs, and visualize the relative positions of a given spacecraft and moon through time, including simulations of the lighting conditions and the sub-Earth, sub-spacecraft, and sub-solar points. Lists of images used to construct the models (available in the SBMT), registered to the shape model but not used in its construction (available in the SBMT), and considered but not used or registered can be found in Additional File 1 (Phobos) and Additional File 2 (Deimos). Chabot et al. (2021) also summarizes the Phobos and Deimos images available in the SBMT, including those registered by our SPC process. The image data are searchable by mission, location, pixel scale, observation time, filter, and viewing geometry. The images can be projected on to the shape model, and the lighting can be set to simulate the conditions under which the data were acquired. The geophysical map and albedo ancillary files can be used to color the plate model.

**Table 8:** Data products and availability

Data product	SBMT	PDS SBN^a^
Global shape models of Phobos (five resolutions, up to 18 m GSD)	x	x
Global shape models of Deimos (four resolutions, up to 20 m GSD)	x	x
High-resolution regional DTMs (54) of Phobos (10 m GSD, global coverage of Phobos’ surface)	x	x
Geophysical map ancillary files for all resolutions of global shape models	x	x
Albedo map ancillary file for the highest-resolution global shape models	x	x
Coregistered and georeferenced image dataset including images from Viking Orbiter, Phobos 2, MOC, HRSC, SRC, and HiRISE	x^b^	
Smithed spk and ck SPICE kernels valid for discrete times of all images used to construct the models and all registered but unused images^b^		x^b^
Adjusted MOLA track	x	x
Image backplanes	x^c^	
Image preview galleries	x	
List of images used to make the models^c^		x^b^

^a^Data products will be submitted to the PDS upon acceptance of this manuscript. Timelines for review and liens will determine when and where these data can be found in this archive

^b^See Additional Files 1 and 2 for image lists

^c^Can be computed by the user on an image-to-image basis

Ultimately, the products listed in Table 8 will be archived in the NASA Planetary Data System (PDS) at the Small Bodies Node (SBN) for long-duration storage and accessibility. The availability (both time and location) of these files will depend on the review and lien process.

## Conclusions and implications

The new high-resolution Phobos and Deimos shape models are in general agreement with existing shape models and offer substantial improvements in the representation of surface details. Our Phobos global model and regional DTMs improve upon the resolution of the previous best-resolved model (Gaskell 2011) by factors of 3.3 and 6, respectively, and improve upon the resolution of the Willner et al. (2014) by factors of 5.6 and 10, respectively. Our Deimos global model represents a factor of 30 improvement in resolution over the Thomas (1993) model, and is the first Deimos shape model to resolve surface features, such as craters. The SPC models of Phobos and Deimos presented here were constructed from the most comprehensive image set to date, which incorporates data from six spacecraft. The estimated accuracy and precision of the global Phobos model are 36 m and 4 m, respectively. The estimated accuracy and precision of the SPC-modeled hemisphere of the Deimos model are 65 m and 9 m, respectively.

These products enable an array of future studies, including, but not limited to, high-resolution topographic measurements, geolocation of surface features, and photometric correction of images and spectra. The coregistered and georeferenced collection of images across six spacecraft provides a rich resource for searching and accessing Phobos and Deimos data. Researchers can query the complete dataset in the SBMT, rather than wading through multiple NASA and ESA data archives that contain mostly images of Mars, rather than the moons.

The new models will also be valuable for future missions that target the martian moons. The Martian Moons eXploration (MMX) mission, led by the Japan Aerospace Exploration Agency (JAXA), will perform a dedicated investigation of Phobos and Deimos in the mid-2020s, culminating in a landing on and sample return from Phobos (Kuramoto et al. 2022). Observations from Phobos orbit and from several Deimos flybys will provide comprehensive datasets of these moons from many instruments. The combination of the new shape models of these bodies and the coregistered image datasets provide a valuable resource for science, observation planning, navigation, landing site selection, and context for MMX measurements. MMX will acquire images of unprecedented pixel scale and coverage from the TENGOO (narrow-angle, panchromatic) and OROCHI (wide-angle, color) cameras (Kameda et al. 2019). These images can be easily registered to the global model and incorporated into the SPC solution to rapidly update the shape. Images of the trailing hemisphere of Phobos at pixel scales ≤ 50 m will be particularly helpful for improving that global model. Images of the anti-Mars and trailing hemispheres of Deimos, in particular, will be critical for improving the shape model and understanding the moon globally. The shape models, coregistered images, and the SBMT combine to make a user-friendly framework to provide context for other MMX instruments as well, including the NASA-funded MEGANE gamma-ray and neutron spectrometer (e.g., Chabot et al. 2021).

## Supplementary Information

**Additional file 1: **Phobos Image Lists.

**Additional file 2: **Deimos Image Lists.

## Data Availability

The data products we have produced as a part of this effort (see Table 8) are available via the Small Body Mapping Tool (https://sbmt.jhuapl.edu) and will be submitted to the NASA PDS SBN for archiving upon acceptance of this manuscript. The original spacecraft images are available at the NASA PDS and the ESA Planetary Science Archive (PSA): Viking Orbiter:VIS: https://pds-geosciences.wustl.edu/missions/viking/visedr.html, Phobos 2:VSK: https://pdssbn.astro.umd.edu/holdings/phb2-m-vsk-2-edr-v1.0/dataset.shtml, MGS:MOC: https://pds-geosciences.wustl.edu/missions/mgs/moc.html, MEX:HRSC (including SRC): https://www.cosmos.esa.int/web/psa/mars-express, MRO:HiRISE: https://pds-imaging.jpl.nasa.gov/portal/mro_mission.html. The NASA PDS Image Atlas is an interactive tool that facilitates searches for Phobos and Deimos images from Viking Orbiter, MOC, and HiRISE: https://pds-imaging.jpl.nasa.gov/search/. The ESA PSA has a web search interface that facilities searches for Phobos and Deimos images from HRSC: https://archives.esac.esa.int/psa/.

## Abbreviations

DTM: Digital Terrain Model
ESA: European Space Agency
GSD: Ground Sample Distance
HiRISE: High Resolution Imaging Science Experiment
HRSC: High Resolution Stereo Camera
JAXA: Japan Aerospace Exploration Agency
MEGANE: Mars-moon Exploration with GAmma rays and NEutrons
MEX: Mars Express
MGS: Mars Global Surveyor
MMX: Martian Moons eXploration
MOC: Mars Orbiter Camera
MOLA: Mars Orbiter Laser Altimeter
MRO: Mars Reconnaissance Orbiter
MSI: Multispectral Imager
NAIF: Navigation and Ancillary Information Facility
NASA: National Aeronautics and Space Administration
NEAR: Near Earth Asteroid Rendezvous
NLR: NEAR Laser Rangefinder
OCAMS: OSIRIS-REx Camera Suite
OLA: OSIRIS-REx Laser Altimeter
OROCHI: Optical RadiOmeter composed of CHromatic Imagers
OSIRIS-REx: Origins, Spectral Interpretation, Resource Identification, and Security-Regolith EXplorer
PDS: Planetary Data System
PSA: Planetary Science Archive
RMS: Root Mean Square
SBMT: Small Body Mapping Tool
SBN: Small Bodies Node
SPC: Stereophotoclinometry
SRC: Super Resolution Channel
TENGOO: TElescopic Nadir imager for GeOmOrphology
USSR: Union of Soviet Socialist Republics
VIS: Visible Imaging Subsystem
VSK: VideoSpectrometric Camera
