# Supplementary material for: High-resolution shape models of Phobos and Deimos from stereophotoclinometry
Source: Earth Planets Space. 2023 Jun 25;75(1):103. doi: 10.1186/s40623-023-01814-7 (PMC10290967; doi:10.1186/s40623-023-01814-7)
Supplement: Supplementary file 1 — Additional file 1: Phobos Image Lists. [file 40623_2023_1814_MOESM1_ESM.docx]

**Additional File 1: Phobos Image Lists**

This file contains three lists of images:

1. Images used to construct the Phobos shape model

These images were fully incorporated into the model and were used to construct topography. These images will be available in the coregistered SBMT dataset, and smithed spacecraft position and attitude for each image will be available at the PDS (see Table 7).

1. Images registered to the Phobos shape model but not used in its construction

These images had one of the following limitations: the image resolution was such that the body was too small to support more than three SPC landmarks; only part of the body was in the scene, and the extent or orientation of the body was such that the image could not support more than three landmarks; minor artifacts were visible that might affect topography but would not interfere with image interpretation; the image was high-phase such that the body appeared as a crescent; poor SNR. These images will be available in the coregistered SBMT dataset, and smithed spacecraft position and attitude for each image will be available at the PDS (see Table 7).

1. Images considered but not used or registered

These images had one of the following limitations: too small to support more than three SPC landmarks; insufficient SPICE information; artifacts across image; only portion of the body is in the image; only a small portion of the image is lit; very blurry; low SNR; distorted (for linescans); striping/speckles/cross-hatching (for linescans); missing data; partial to complete saturation. This list is supplied as a resource for someone wishing to use Phobos images in the future. These images will not be available in the SBMT, nor will smithed spacecraft position and altitude be available at the PDS.

**Images used to construct the Phobos shape model**

**#Viking**

f039b82.imq

f039b83.imq

f039b84.imq

f047b03.imq

f055a32.imq

f057a51.imq

f057a53.imq

f071b03.imq

f073b03.imq

f075b43.imq

f077b03.imq

f079b41.imq

f081b33.imq

f087a52.imq

f101a77.imq

f103a43.imq

f110a01.imq

f110a03.imq

f115a01.imq

f115a03.imq

f115a05.imq

f115a07.imq

f119b03.imq

f123b03.imq

f124a85.imq

f138b03.imq

f143b61.imq

f143b63.imq

f143b65.imq

f143b67.imq

f149b12.imq

f149b14.imq

f149b16.imq

f149b18.imq

f149b20.imq

f149b22.imq

f149b24.imq

f149b26.imq

f203a15.imq

f203a17.imq

f242a04.imq

f242a06.imq

f242a17.imq

f242a18.imq

f242a19.imq

f242a20.imq

f242a21.imq

f242a22.imq

f243a68.imq

f243a69.imq

f243a70.imq

f243a71.imq

f243a72.imq

f244a03.imq

f244a04.imq

f244a05.imq

f244a06.imq

f244a07.imq

f244a69.imq

f244a70.imq

f244a71.imq

f244a72.imq

f246a03.imq

f246a04.imq

f246a05.imq

f246a06.imq

f246a07.imq

f246a08.imq

f246a09.imq

f246a54.imq

f246a55.imq

f246a56.imq

f246a57.imq

f246a58.imq

f246a59.imq

f246a60.imq

f246a61.imq

f246a62.imq

f246a63.imq

f246a64.imq

f246a65.imq

f246a66.imq

f246a67.imq

f246a68.imq

f246a70.imq

f246a72.imq

f248a01.imq

f248a02.imq

f248a03.imq

f248a04.imq

f248a05.imq

f248a06.imq

f249a01.imq

f249a02.imq

f249a03.imq

f249a04.imq

f249a05.imq

f249a06.imq

f250a09.imq

f250a10.imq

f250a11.imq

f250a12.imq

f250a13.imq

f250a14.imq

f250a15.imq

f250a16.imq

f250a58.imq

f250a59.imq

f250a60.imq

f250a61.imq

f250a62.imq

f250a63.imq

f250a64.imq

f250a65.imq

f250a66.imq

f250a67.imq

f250a68.imq

f250a69.imq

f250a70.imq

f250a71.imq

f250a72.imq

f252a13.imq

f252a15.imq

f252a16.imq

f252a58.imq

f252a59.imq

f252a60.imq

f252a61.imq

f252a62.imq

f252a63.imq

f252a64.imq

f252a65.imq

f292a01.imq

f292a02.imq

f292a03.imq

f292a04.imq

f292a05.imq

f292a06.imq

f292a07.imq

f292a08.imq

f292a09.imq

f292a10.imq

f315a01.imq

f315a11.imq

f315a12.imq

f315a13.imq

f329a81.imq

f329a83.imq

f329a85.imq

f334b04.imq

f334b06.imq

f343a08.imq

f343a09.imq

f343a10.imq

f343a11.imq

f343a12.imq

f343a13.imq

f343a14.imq

f343a15.imq

f343a17.imq

f343a25.imq

f343a27.imq

f343a29.imq

f343a30.imq

f343a31.imq

f343a32.imq

f343a33.imq

f357a34.imq

f357a35.imq

f357a64.imq

f357a66.imq

f371a10.imq

f371a11.imq

f371a12.imq

f371a40.imq

f371a41.imq

f372a01.imq

f372a03.imq

f382a02.imq

f382a03.imq

f390a06.imq

f390a07.imq

f390a08.imq

f390a13.imq

f390a14.imq

f390a16.imq

f405a04.imq

f405a05.imq

f413a21.imq

f413a23.imq

f413a25.imq

f413a27.imq

f413a29.imq

f413a31.imq

f413a33.imq

f420a01.imq

f420a03.imq

f420a05.imq

f420a07.imq

f420a09.imq

f436a41.imq

f436a43.imq

f458a01.imq

f458a03.imq

f458a05.imq

f458a07.imq

f458a09.imq

f458a11.imq

f458a13.imq

f794a55.imq

f854a61.imq

f854a62.imq

f854a63.imq

f854a81.imq

f854a82.imq

f854a83.imq

**#Phobos2**

c2230052.img

c2300012.img

c2300042.img

c2300072.img

c2550012.img

c2550042.img

c2550132.img

**#MOC**

sp247603.imq

sp250103.imq

sp252603.imq

sp252604.imq

**#SRC (HRSC framing)**

H0413_0002_SR2.IMG

H0413_0003_SR2.IMG

H0413_0004_SR2.IMG

H0413_0005_SR2.IMG

H0649_0002_SR2.IMG

H0649_0003_SR2.IMG

H0715_0002_SR2.IMG

H0715_0003_SR2.IMG

H0715_0004_SR2.IMG

H0715_0005_SR2.IMG

H0715_0006_SR2.IMG

H0748_0003_SR2.IMG

H0748_0004_SR2.IMG

H0748_0005_SR2.IMG

H1064_0004_SR2.IMG

H1064_0005_SR2.IMG

H1064_0006_SR2.IMG

H1163_0002_SR2.IMG

H1163_0003_SR2.IMG

H1163_0004_SR2.IMG

H1558_0005_SR2.IMG

H1574_0005_SR2.IMG

H1607_0005_SR2.IMG

H1607_0006_SR2.IMG

H1901_0011_SR2.IMG

H1901_0012_SR2.IMG

H1901_0013_SR2.IMG

H1901_0014_SR2.IMG

H2151_0004_SR2.IMG

H2151_0005_SR2.IMG

H2233_0004_SR2.IMG

H2233_0005_SR2.IMG

H2381_0005_SR2.IMG

H2381_0006_SR2.IMG

H2397_0002_SR2.IMG

H2397_0003_SR2.IMG

H2446_0004_SR2.IMG

H2446_0005_SR2.IMG

H2463_0004_SR2.IMG

H2463_0005_SR2.IMG

H2479_0004_SR2.IMG

H2479_0005_SR2.IMG

H2479_0006_SR2.IMG

H2479_0007_SR2.IMG

H2487_0003_SR2.IMG

H2487_0004_SR2.IMG

H2487_0005_SR2.IMG

H2487_0006_SR2.IMG

H2501_0004_SR2.IMG

H2501_0005_SR2.IMG

H2501_0006_SR2.IMG

H2601_0005_SR2.IMG

H2601_0006_SR2.IMG

H2601_0007_SR2.IMG

H2643_0004_SR2.IMG

H2643_0005_SR2.IMG

H2643_0006_SR2.IMG

H2673_0004_SR2.IMG

H2673_0005_SR2.IMG

H2673_0006_SR2.IMG

H2682_0003_SR2.IMG

H2682_0004_SR2.IMG

H2682_0005_SR2.IMG

H2682_0006_SR2.IMG

H2706_0004_SR2.IMG

H2706_0005_SR2.IMG

H2739_0004_SR2.IMG

H2739_0005_SR2.IMG

H2739_0006_SR2.IMG

H2756_0003_SR2.IMG

H2756_0004_SR2.IMG

H2756_0005_SR2.IMG

H2756_0006_SR2.IMG

H2780_0002_SR2.IMG

H2780_0003_SR2.IMG

H2780_0004_SR2.IMG

H2780_0005_SR2.IMG

H2780_0006_SR2.IMG

H2805_0022_SR2.IMG

H2805_0023_SR2.IMG

H2805_0024_SR2.IMG

H2805_0025_SR2.IMG

H2813_0004_SR2.IMG

H2813_0005_SR2.IMG

H2813_0006_SR2.IMG

H2813_0007_SR2.IMG

H2846_0006_SR2.IMG

H2846_0007_SR2.IMG

H2854_0004_SR2.IMG

H2854_0005_SR2.IMG

H2912_0004_SR2.IMG

H2912_0005_SR2.IMG

H2912_0006_SR2.IMG

H2979_0006_SR2.IMG

H2979_0007_SR2.IMG

H2979_0008_SR2.IMG

H3005_0005_SR2.IMG

H3005_0006_SR2.IMG

H3005_0007_SR2.IMG

H3245_0004_SR2.IMG

H3245_0005_SR2.IMG

H3245_0006_SR2.IMG

H3245_0007_SR2.IMG

H3310_0002_SR2.IMG

H3310_0003_SR2.IMG

H3310_0004_SR2.IMG

H3310_0005_SR2.IMG

H3310_0006_SR2.IMG

H3761_0004_SR2.IMG

H3761_0005_SR2.IMG

H3769_0003_SR2.IMG

H3769_0004_SR2.IMG

H3769_0005_SR2.IMG

H3802_0003_SR2.IMG

H3802_0004_SR2.IMG

H3802_0005_SR2.IMG

H3835_0005_SR2.IMG

H3835_0006_SR2.IMG

H3835_0007_SR2.IMG

H3843_0003_SR2.IMG

H3843_0004_SR2.IMG

H3843_0005_SR2.IMG

H3843_0006_SR2.IMG

H3868_0003_SR2.IMG

H3868_0004_SR2.IMG

H3868_0005_SR2.IMG

H3876_0003_SR2.IMG

H3876_0004_SR2.IMG

H3876_0005_SR2.IMG

H3909_0004_SR2.IMG

H3909_0005_SR2.IMG

H3942_0004_SR2.IMG

H3942_0005_SR2.IMG

H3999_0003_SR2.IMG

H3999_0004_SR2.IMG

H3999_0005_SR2.IMG

H4030_0003_SR2.IMG

H4030_0004_SR2.IMG

H4030_0005_SR2.IMG

H4030_0006_SR2.IMG

H4233_0004_SR2.IMG

H4233_0005_SR2.IMG

H4233_0006_SR2.IMG

H4274_0003_SR2.IMG

H4274_0004_SR2.IMG

H4274_0005_SR2.IMG

H4307_0003_SR2.IMG

H4307_0004_SR2.IMG

H4307_0005_SR2.IMG

H4307_0006_SR2.IMG

H4307_0007_SR2.IMG

H4332_0004_SR2.IMG

H4332_0005_SR2.IMG

H4340_0002_SR2.IMG

H4340_0003_SR2.IMG

H4340_0004_SR2.IMG

H4340_0005_SR2.IMG

H4373_0003_SR2.IMG

H4373_0004_SR2.IMG

H4373_0005_SR2.IMG

H4381_0004_SR2.IMG

H4381_0005_SR2.IMG

H4381_0006_SR2.IMG

H4414_0003_SR2.IMG

H4414_0004_SR2.IMG

H4414_0005_SR2.IMG

H4414_0006_SR2.IMG

H4447_0004_SR2.IMG

H4447_0005_SR2.IMG

H4447_0006_SR2.IMG

H4554_0004_SR2.IMG

H4554_0005_SR2.IMG

H4603_0005_SR2.IMG

H4603_0006_SR2.IMG

H4603_0007_SR2.IMG

H4636_0004_SR2.IMG

H4636_0005_SR2.IMG

H4683_0005_SR2.IMG

H4683_0006_SR2.IMG

H4765_0005_SR2.IMG

H4765_0006_SR2.IMG

H4773_0003_SR2.IMG

H4773_0004_SR2.IMG

H4773_0005_SR2.IMG

H4806_0005_SR2.IMG

H4806_0006_SR2.IMG

H4806_0007_SR2.IMG

H4847_0004_SR2.IMG

H4847_0005_SR2.IMG

H4847_0006_SR2.IMG

H4855_0004_SR2.IMG

H4855_0005_SR2.IMG

H4880_0004_SR2.IMG

H4880_0005_SR2.IMG

H4888_0004_SR2.IMG

H4888_0005_SR2.IMG

H4888_0006_SR2.IMG

H4913_0004_SR2.IMG

H4913_0005_SR2.IMG

H4913_0006_SR2.IMG

H4946_0004_SR2.IMG

H4946_0005_SR2.IMG

H4946_0006_SR2.IMG

H5163_0005_SR2.IMG

H5163_0006_SR2.IMG

H5163_0007_SR2.IMG

H5277_0005_SR2.IMG

H5277_0006_SR2.IMG

H5277_0007_SR2.IMG

H5305_0003_SR2.IMG

H5305_0004_SR2.IMG

H5343_0002_SR2.IMG

H5343_0003_SR2.IMG

H5343_0004_SR2.IMG

H5343_0005_SR2.IMG

H5362_0002_SR2.IMG

H5362_0003_SR2.IMG

H5362_0004_SR2.IMG

H5362_0005_SR2.IMG

H5381_0003_SR2.IMG

H5381_0004_SR2.IMG

H5381_0005_SR2.IMG

H5428_0004_SR2.IMG

H5428_0005_SR2.IMG

H5428_0006_SR2.IMG

H5447_0004_SR2.IMG

H5447_0005_SR2.IMG

H5447_0006_SR2.IMG

H5504_0003_SR2.IMG

H5504_0004_SR2.IMG

H5504_0005_SR2.IMG

H5552_0004_SR2.IMG

H5552_0005_SR2.IMG

H5552_0006_SR2.IMG

H5552_0007_SR2.IMG

H5699_0006_SR2.IMG

H5699_0007_SR2.IMG

H5766_0004_SR2.IMG

H5766_0005_SR2.IMG

H5766_0006_SR2.IMG

H5850_0004_SR2.IMG

H5850_0005_SR2.IMG

H5850_0006_SR2.IMG

H5861_0005_SR2.IMG

H5870_0002_SR2.IMG

H5870_0003_SR2.IMG

H5870_0004_SR2.IMG

H5870_0005_SR2.IMG

H5870_0006_SR2.IMG

H5889_0002_SR2.IMG

H5889_0003_SR2.IMG

H5889_0004_SR2.IMG

H5889_0005_SR2.IMG

H5908_0004_SR2.IMG

H5908_0005_SR2.IMG

H5984_0004_SR2.IMG

H5984_0005_SR2.IMG

H6042_0004_SR2.IMG

H6042_0005_SR2.IMG

H6042_0006_SR2.IMG

H6128_0002_SR2.IMG

H6128_0003_SR2.IMG

H6128_0004_SR2.IMG

H6128_0005_SR2.IMG

H6128_0006_SR2.IMG

H6128_0007_SR2.IMG

H6217_0003_SR2.IMG

H6217_0004_SR2.IMG

H6217_0005_SR2.IMG

H6217_0006_SR2.IMG

H6217_0007_SR2.IMG

H6551_0004_SR2.IMG

H6551_0005_SR2.IMG

H6551_0006_SR2.IMG

H6637_0004_SR2.IMG

H6637_0005_SR2.IMG

H6745_0004_SR2.IMG

H6745_0005_SR2.IMG

H6748_0003_SR2.IMG

H6748_0004_SR2.IMG

H6748_0005_SR2.IMG

H6748_0006_SR2.IMG

H6757_0005_SR2.IMG

H6757_0006_SR2.IMG

H6757_0007_SR2.IMG

H6896_0027_SR2.IMG

H6896_0028_SR2.IMG

H6896_0029_SR2.IMG

H6896_0030_SR2.IMG

H6896_0031_SR2.IMG

H6896_0032_SR2.IMG

H6896_0033_SR2.IMG

H6896_0034_SR2.IMG

H6896_0035_SR2.IMG

H6896_0036_SR2.IMG

H6896_0037_SR2.IMG

H6896_0038_SR2.IMG

H6896_0039_SR2.IMG

H6896_0040_SR2.IMG

H6896_0041_SR2.IMG

H6896_0042_SR2.IMG

H6896_0043_SR2.IMG

H6896_0044_SR2.IMG

H6896_0045_SR2.IMG

H6896_0046_SR2.IMG

H6896_0047_SR2.IMG

H6896_0048_SR2.IMG

H6896_0049_SR2.IMG

H6896_0050_SR2.IMG

H6906_0005_SR2.IMG

H6906_0006_SR2.IMG

H6916_0003_SR2.IMG

H6916_0004_SR2.IMG

H6916_0005_SR2.IMG

H6916_0006_SR2.IMG

H6926_0005_SR2.IMG

H6926_0006_SR2.IMG

H6987_0005_SR2.IMG

H6987_0006_SR2.IMG

H7017_0005_SR2.IMG

H7017_0006_SR2.IMG

H7048_0005_SR2.IMG

H7048_0006_SR2.IMG

H7088_0005_SR2.IMG

H7088_0006_SR2.IMG

H7088_0007_SR2.IMG

H7109_0006_SR2.IMG

H7109_0007_SR2.IMG

H7109_0008_SR2.IMG

H7225_0005_SR2.IMG

H7225_0006_SR2.IMG

H7407_0006_SR2.IMG

H7407_0007_SR2.IMG

H7488_0006_SR2.IMG

H7488_0007_SR2.IMG

H7492_0042_SR2.IMG

H7492_0043_SR2.IMG

H7492_0044_SR2.IMG

H7492_0045_SR2.IMG

H7492_0046_SR2.IMG

H7492_0047_SR2.IMG

H7492_0048_SR2.IMG

H7492_0049_SR2.IMG

H7492_0050_SR2.IMG

H7492_0051_SR2.IMG

H7492_0052_SR2.IMG

H7492_0053_SR2.IMG

H7492_0054_SR2.IMG

H7492_0055_SR2.IMG

H7492_0056_SR2.IMG

H7492_0057_SR2.IMG

H7492_0058_SR2.IMG

H7492_0059_SR2.IMG

H7492_0060_SR2.IMG

H7492_0061_SR2.IMG

H7492_0062_SR2.IMG

H7492_0063_SR2.IMG

H7492_0064_SR2.IMG

H7492_0065_SR2.IMG

H7492_0066_SR2.IMG

H7492_0067_SR2.IMG

H7492_0068_SR2.IMG

H7492_0069_SR2.IMG

H7492_0070_SR2.IMG

H7492_0071_SR2.IMG

H7492_0072_SR2.IMG

H7492_0073_SR2.IMG

H7492_0074_SR2.IMG

H7492_0075_SR2.IMG

H7492_0076_SR2.IMG

H7492_0077_SR2.IMG

H7492_0078_SR2.IMG

H7719_0005_SR2.IMG

H7719_0006_SR2.IMG

H7719_0007_SR2.IMG

H7742_0004_SR2.IMG

H7742_0005_SR2.IMG

H7742_0006_SR2.IMG

H7800_0003_SR2.IMG

H7800_0004_SR2.IMG

H7800_0005_SR2.IMG

H7800_0006_SR2.IMG

H7813_0004_SR2.IMG

H7813_0005_SR2.IMG

H7813_0006_SR2.IMG

H7813_0007_SR2.IMG

H7872_0003_SR2.IMG

H7872_0004_SR2.IMG

H7872_0005_SR2.IMG

H7926_0011_SR2.IMG

H7937_0002_SR2.IMG

H7937_0003_SR2.IMG

H7937_0004_SR2.IMG

H7937_0005_SR2.IMG

H7937_0006_SR2.IMG

H7948_0003_SR2.IMG

H7948_0004_SR2.IMG

H7948_0005_SR2.IMG

H7948_0006_SR2.IMG

H7959_0004_SR2.IMG

H7959_0005_SR2.IMG

H7959_0006_SR2.IMG

H7982_0005_SR2.IMG

H7982_0006_SR2.IMG

H8017_0004_SR2.IMG

H8052_0004_SR2.IMG

H8052_0005_SR2.IMG

H8088_0005_SR2.IMG

H8146_0003_SR2.IMG

H8146_0004_SR2.IMG

H8146_0005_SR2.IMG

H8146_0006_SR2.IMG

H8193_0004_SR2.IMG

H8193_0005_SR2.IMG

H8193_0006_SR2.IMG

H8217_0004_SR2.IMG

H8217_0005_SR2.IMG

H8217_0006_SR2.IMG

H8276_0004_SR2.IMG

H8276_0005_SR2.IMG

H8276_0006_SR2.IMG

H8326_0004_SR2.IMG

H8326_0005_SR2.IMG

H8326_0006_SR2.IMG

H8396_0004_SR2.IMG

H8396_0005_SR2.IMG

H8396_0006_SR2.IMG

H8477_0002_SR2.IMG

H8477_0003_SR2.IMG

H8477_0004_SR2.IMG

H8477_0005_SR2.IMG

H8512_0003_SR2.IMG

H8512_0004_SR2.IMG

H8512_0005_SR2.IMG

H8512_0006_SR2.IMG

H8535_0004_SR2.IMG

H8535_0005_SR2.IMG

H8535_0006_SR2.IMG

H8535_0007_SR2.IMG

H8570_0004_SR2.IMG

H8570_0005_SR2.IMG

H8570_0006_SR2.IMG

H8663_0004_SR2.IMG

H8663_0005_SR2.IMG

H8663_0006_SR2.IMG

H8746_0004_SR2.IMG

H8746_0005_SR2.IMG

H8746_0006_SR2.IMG

H8870_0004_SR2.IMG

H8870_0005_SR2.IMG

H8870_0006_SR2.IMG

H8870_0007_SR2.IMG

H8951_0004_SR2.IMG

H8951_0005_SR2.IMG

H8951_0006_SR2.IMG

H8963_0004_SR2.IMG

H8963_0005_SR2.IMG

H8963_0006_SR2.IMG

H8986_0003_SR2.IMG

H8986_0004_SR2.IMG

H8986_0005_SR2.IMG

H8998_0004_SR2.IMG

H8998_0005_SR2.IMG

H8998_0006_SR2.IMG

H9161_0004_SR2.IMG

H9161_0005_SR2.IMG

H9161_0006_SR2.IMG

H9208_0003_SR2.IMG

H9208_0004_SR2.IMG

H9208_0005_SR2.IMG

H9290_0004_SR2.IMG

H9290_0005_SR2.IMG

H9290_0006_SR2.IMG

H9365_0004_SR2.IMG

H9365_0005_SR2.IMG

H9365_0006_SR2.IMG

H9435_0004_SR2.IMG

H9435_0005_SR2.IMG

H9435_0006_SR2.IMG

H9463_0021_SR2.IMG

H9463_0022_SR2.IMG

H9463_0023_SR2.IMG

H9463_0024_SR2.IMG

H9463_0025_SR2.IMG

H9463_0026_SR2.IMG

H9463_0027_SR2.IMG

H9463_0029_SR2.IMG

H9463_0030_SR2.IMG

H9463_0031_SR2.IMG

H9463_0032_SR2.IMG

H9463_0033_SR2.IMG

H9463_0034_SR2.IMG

H9463_0035_SR2.IMG

H9463_0036_SR2.IMG

H9463_0037_SR2.IMG

H9463_0038_SR2.IMG

H9463_0039_SR2.IMG

H9463_0040_SR2.IMG

H9463_0041_SR2.IMG

H9463_0042_SR2.IMG

H9463_0043_SR2.IMG

H9463_0044_SR2.IMG

H9463_0045_SR2.IMG

H9463_0046_SR2.IMG

H9463_0047_SR2.IMG

H9463_0048_SR2.IMG

H9463_0049_SR2.IMG

H9463_0050_SR2.IMG

H9463_0051_SR2.IMG

H9463_0052_SR2.IMG

H9463_0053_SR2.IMG

H9463_0054_SR2.IMG

H9463_0055_SR2.IMG

H9463_0056_SR2.IMG

H9463_0057_SR2.IMG

H9463_0058_SR2.IMG

H9463_0059_SR2.IMG

H9463_0060_SR2.IMG

H9463_0061_SR2.IMG

H9463_0062_SR2.IMG

H9463_0063_SR2.IMG

H9463_0064_SR2.IMG

H9463_0065_SR2.IMG

H9463_0066_SR2.IMG

H9463_0067_SR2.IMG

H9463_0068_SR2.IMG

H9463_0069_SR2.IMG

H9463_0070_SR2.IMG

H9463_0071_SR2.IMG

H9463_0072_SR2.IMG

H9463_0073_SR2.IMG

H9463_0074_SR2.IMG

H9463_0075_SR2.IMG

H9463_0076_SR2.IMG

H9463_0077_SR2.IMG

H9463_0078_SR2.IMG

H9463_0079_SR2.IMG

H9463_0080_SR2.IMG

H9463_0081_SR2.IMG

H9463_0082_SR2.IMG

H9463_0083_SR2.IMG

H9463_0084_SR2.IMG

H9463_0085_SR2.IMG

H9463_0087_SR2.IMG

H9517_0004_SR2.IMG

H9517_0005_SR2.IMG

H9517_0006_SR2.IMG

H9551_0004_SR2.IMG

H9551_0005_SR2.IMG

H9551_0006_SR2.IMG

H9551_0007_SR2.IMG

H9574_0004_SR2.IMG

H9574_0005_SR2.IMG

H9574_0006_SR2.IMG

H9574_0007_SR2.IMG

H9574_0008_SR2.IMG

H9586_0004_SR2.IMG

H9586_0005_SR2.IMG

HB908_0004_SR2.IMG

HB908_0005_SR2.IMG

HB911_0003_SR2.IMG

HB911_0004_SR2.IMG

HB911_0005_SR2.IMG

HB911_0006_SR2.IMG

HB940_0004_SR2.IMG

HB940_0005_SR2.IMG

HB940_0006_SR2.IMG

HB963_0004_SR2.IMG

HB963_0005_SR2.IMG

HB963_0006_SR2.IMG

HB992_0004_SR2.IMG

HB992_0005_SR2.IMG

HB992_0006_SR2.IMG

HC046_0002_SR2.IMG

HC046_0003_SR2.IMG

HC046_0004_SR2.IMG

HC046_0005_SR2.IMG

HC046_0006_SR2.IMG

HC069_0003_SR2.IMG

HC069_0004_SR2.IMG

HC069_0005_SR2.IMG

HC069_0006_SR2.IMG

HC092_0006_SR2.IMG

HC092_0007_SR2.IMG

HC092_0008_SR2.IMG

HC092_0009_SR2.IMG

HC103_0005_SR2.IMG

HC103_0006_SR2.IMG

HC127_0002_SR2.IMG

HC127_0003_SR2.IMG

HC127_0004_SR2.IMG

HC127_0005_SR2.IMG

HC127_0006_SR2.IMG

HC146_0016_SR2.IMG

HC146_0017_SR2.IMG

HC146_0018_SR2.IMG

HC146_0019_SR2.IMG

HC146_0020_SR2.IMG

HC146_0021_SR2.IMG

HC146_0022_SR2.IMG

HC146_0023_SR2.IMG

HC146_0024_SR2.IMG

HC146_0025_SR2.IMG

HC146_0026_SR2.IMG

HC146_0027_SR2.IMG

HC146_0028_SR2.IMG

HC146_0029_SR2.IMG

HC146_0030_SR2.IMG

HC146_0031_SR2.IMG

HC146_0032_SR2.IMG

HC146_0033_SR2.IMG

HC146_0034_SR2.IMG

HC146_0035_SR2.IMG

HC146_0036_SR2.IMG

HC146_0037_SR2.IMG

HC146_0038_SR2.IMG

HC146_0039_SR2.IMG

HC146_0040_SR2.IMG

HC146_0041_SR2.IMG

HC146_0042_SR2.IMG

HC146_0043_SR2.IMG

HC146_0044_SR2.IMG

HC146_0045_SR2.IMG

HC146_0046_SR2.IMG

HC146_0047_SR2.IMG

HC146_0048_SR2.IMG

HC146_0049_SR2.IMG

HC146_0050_SR2.IMG

HC146_0051_SR2.IMG

HC146_0052_SR2.IMG

HC146_0053_SR2.IMG

HC146_0054_SR2.IMG

HC146_0055_SR2.IMG

HC146_0056_SR2.IMG

HC146_0057_SR2.IMG

HC146_0058_SR2.IMG

HC146_0059_SR2.IMG

HC146_0060_SR2.IMG

HC146_0061_SR2.IMG

HC146_0062_SR2.IMG

HC146_0063_SR2.IMG

HC150_0004_SR2.IMG

HC150_0005_SR2.IMG

HC150_0006_SR2.IMG

HC151_0022_SR2.IMG

HC151_0023_SR2.IMG

HC151_0024_SR2.IMG

HC151_0025_SR2.IMG

HC151_0026_SR2.IMG

HC151_0027_SR2.IMG

HC151_0028_SR2.IMG

HC151_0029_SR2.IMG

HC151_0030_SR2.IMG

HC151_0031_SR2.IMG

HC151_0033_SR2.IMG

HC151_0034_SR2.IMG

HC151_0035_SR2.IMG

HC151_0036_SR2.IMG

HC151_0037_SR2.IMG

HC151_0038_SR2.IMG

HC151_0039_SR2.IMG

HC151_0040_SR2.IMG

HC151_0041_SR2.IMG

HC151_0042_SR2.IMG

HC151_0043_SR2.IMG

HC151_0044_SR2.IMG

HC151_0045_SR2.IMG

HC151_0046_SR2.IMG

HC151_0047_SR2.IMG

HC151_0048_SR2.IMG

HC151_0049_SR2.IMG

HC151_0050_SR2.IMG

HC151_0051_SR2.IMG

HC151_0052_SR2.IMG

HC151_0053_SR2.IMG

HC151_0054_SR2.IMG

HC151_0055_SR2.IMG

HC151_0056_SR2.IMG

HC151_0057_SR2.IMG

HC151_0058_SR2.IMG

HC151_0059_SR2.IMG

HC151_0060_SR2.IMG

HC151_0061_SR2.IMG

HC151_0062_SR2.IMG

HC151_0063_SR2.IMG

HC151_0064_SR2.IMG

HC151_0065_SR2.IMG

HC151_0066_SR2.IMG

HC151_0067_SR2.IMG

HC151_0068_SR2.IMG

HC151_0069_SR2.IMG

HC151_0070_SR2.IMG

HC151_0071_SR2.IMG

HC151_0072_SR2.IMG

HC151_0073_SR2.IMG

HC151_0074_SR2.IMG

HC151_0075_SR2.IMG

HC151_0076_SR2.IMG

HC151_0077_SR2.IMG

HC151_0078_SR2.IMG

HC151_0079_SR2.IMG

HC151_0080_SR2.IMG

HC151_0081_SR2.IMG

HC151_0082_SR2.IMG

HC151_0083_SR2.IMG

HC151_0084_SR2.IMG

HC151_0085_SR2.IMG

HC151_0086_SR2.IMG

HC151_0087_SR2.IMG

HC151_0088_SR2.IMG

HC151_0089_SR2.IMG

HC151_0090_SR2.IMG

HC151_0091_SR2.IMG

HC151_0092_SR2.IMG

HC151_0093_SR2.IMG

HC151_0094_SR2.IMG

HC151_0095_SR2.IMG

HC151_0096_SR2.IMG

HC151_0097_SR2.IMG

HC151_0098_SR2.IMG

HC151_0099_SR2.IMG

HC151_0100_SR2.IMG

HC151_0101_SR2.IMG

HC151_0102_SR2.IMG

HC151_0103_SR2.IMG

HC151_0104_SR2.IMG

HC151_0105_SR2.IMG

HC151_0106_SR2.IMG

HC151_0107_SR2.IMG

HC151_0108_SR2.IMG

HC151_0109_SR2.IMG

HC151_0110_SR2.IMG

HC151_0111_SR2.IMG

HC151_0112_SR2.IMG

HC151_0113_SR2.IMG

HC151_0114_SR2.IMG

HC151_0115_SR2.IMG

HC151_0116_SR2.IMG

HC151_0117_SR2.IMG

HC151_0118_SR2.IMG

HC151_0119_SR2.IMG

HC173_0004_SR2.IMG

HC173_0005_SR2.IMG

HC173_0006_SR2.IMG

HC196_0004_SR2.IMG

HC196_0005_SR2.IMG

HC208_0004_SR2.IMG

HC208_0005_SR2.IMG

HC208_0006_SR2.IMG

HC266_0003_SR2.IMG

HC266_0004_SR2.IMG

HC266_0005_SR2.IMG

HC266_0006_SR2.IMG

HC279_0022_SR2.IMG

HC279_0023_SR2.IMG

HC279_0024_SR2.IMG

HC279_0025_SR2.IMG

HC279_0026_SR2.IMG

HC279_0027_SR2.IMG

HC279_0028_SR2.IMG

HC279_0029_SR2.IMG

HC279_0030_SR2.IMG

HC279_0031_SR2.IMG

HC279_0033_SR2.IMG

HC279_0034_SR2.IMG

HC279_0035_SR2.IMG

HC279_0036_SR2.IMG

HC279_0037_SR2.IMG

HC279_0038_SR2.IMG

HC279_0039_SR2.IMG

HC279_0040_SR2.IMG

HC279_0041_SR2.IMG

HC279_0042_SR2.IMG

HC279_0043_SR2.IMG

HC279_0044_SR2.IMG

HC279_0045_SR2.IMG

HC279_0046_SR2.IMG

HC279_0047_SR2.IMG

HC279_0048_SR2.IMG

HC279_0049_SR2.IMG

HC279_0050_SR2.IMG

HC279_0051_SR2.IMG

HC279_0052_SR2.IMG

HC279_0053_SR2.IMG

HC279_0054_SR2.IMG

HC279_0055_SR2.IMG

HC279_0056_SR2.IMG

HC279_0057_SR2.IMG

HC279_0058_SR2.IMG

HC279_0059_SR2.IMG

HC279_0060_SR2.IMG

HC279_0061_SR2.IMG

HC279_0062_SR2.IMG

HC279_0063_SR2.IMG

HC279_0064_SR2.IMG

HC279_0065_SR2.IMG

HC279_0066_SR2.IMG

HC279_0067_SR2.IMG

HC279_0068_SR2.IMG

HC279_0069_SR2.IMG

HC279_0070_SR2.IMG

HC279_0071_SR2.IMG

HC279_0072_SR2.IMG

HC279_0073_SR2.IMG

HC279_0074_SR2.IMG

HC279_0075_SR2.IMG

HC279_0076_SR2.IMG

HC279_0077_SR2.IMG

HC279_0078_SR2.IMG

HC279_0079_SR2.IMG

HC279_0080_SR2.IMG

HC279_0081_SR2.IMG

HC279_0082_SR2.IMG

HC279_0083_SR2.IMG

HC279_0084_SR2.IMG

HC301_0003_SR2.IMG

HC301_0004_SR2.IMG

HC301_0005_SR2.IMG

HC301_0006_SR2.IMG

HC301_0007_SR2.IMG

HC348_0003_SR2.IMG

HC348_0004_SR2.IMG

HC348_0005_SR2.IMG

HC348_0006_SR2.IMG

HC383_0003_SR2.IMG

HC383_0004_SR2.IMG

HC383_0005_SR2.IMG

HC383_0006_SR2.IMG

HC491_0004_SR2.IMG

HC491_0005_SR2.IMG

HC491_0006_SR2.IMG

HC525_0004_SR2.IMG

HC525_0005_SR2.IMG

HC525_0006_SR2.IMG

HC583_0004_SR2.IMG

HC583_0005_SR2.IMG

HC606_0004_SR2.IMG

HC606_0005_SR2.IMG

HC629_0005_SR2.IMG

HC629_0006_SR2.IMG

HC629_0007_SR2.IMG

HC652_0004_SR2.IMG

HC652_0005_SR2.IMG

HC652_0006_SR2.IMG

HC664_0003_SR2.IMG

HC664_0004_SR2.IMG

HC664_0005_SR2.IMG

HC664_0006_SR2.IMG

HC675_0004_SR2.IMG

HC675_0005_SR2.IMG

HC675_0006_SR2.IMG

HC675_0007_SR2.IMG

HC687_0003_SR2.IMG

HC687_0004_SR2.IMG

HC687_0005_SR2.IMG

HC687_0006_SR2.IMG

HC710_0005_SR2.IMG

HC710_0006_SR2.IMG

HC710_0007_SR2.IMG

HC733_0006_SR2.IMG

HC733_0007_SR2.IMG

HC791_0003_SR2.IMG

HC791_0004_SR2.IMG

HC791_0005_SR2.IMG

HC808_0004_SR2.IMG

HC808_0005_SR2.IMG

HC808_0006_SR2.IMG

HC836_0003_SR2.IMG

HC836_0004_SR2.IMG

HC836_0005_SR2.IMG

HC837_0003_SR2.IMG

HC837_0004_SR2.IMG

HC837_0005_SR2.IMG

HC837_0006_SR2.IMG

HC871_0002_SR2.IMG

HC871_0003_SR2.IMG

HC871_0004_SR2.IMG

HC871_0005_SR2.IMG

HC871_0006_SR2.IMG

HC901_0004_SR2.IMG

HC901_0005_SR2.IMG

HC901_0006_SR2.IMG

HC917_0004_SR2.IMG

HC917_0005_SR2.IMG

HC917_0006_SR2.IMG

HC928_0003_SR2.IMG

HC928_0004_SR2.IMG

HC928_0005_SR2.IMG

HC928_0006_SR2.IMG

HC959_0004_SR2.IMG

HC959_0005_SR2.IMG

HC959_0006_SR2.IMG

HC963_0015_SR2.IMG

HC963_0016_SR2.IMG

HC963_0017_SR2.IMG

HC963_0018_SR2.IMG

HC963_0019_SR2.IMG

HC963_0020_SR2.IMG

HC963_0021_SR2.IMG

HC963_0022_SR2.IMG

HC963_0023_SR2.IMG

HC963_0024_SR2.IMG

HC963_0025_SR2.IMG

HC963_0026_SR2.IMG

HC963_0027_SR2.IMG

HC963_0028_SR2.IMG

HC963_0029_SR2.IMG

HC963_0030_SR2.IMG

HC963_0031_SR2.IMG

HC963_0032_SR2.IMG

HC963_0033_SR2.IMG

HC963_0034_SR2.IMG

HC963_0035_SR2.IMG

HC963_0036_SR2.IMG

HC963_0037_SR2.IMG

HC963_0038_SR2.IMG

HC963_0039_SR2.IMG

HC963_0040_SR2.IMG

HC963_0041_SR2.IMG

HC963_0042_SR2.IMG

HC963_0043_SR2.IMG

HC963_0044_SR2.IMG

HC963_0045_SR2.IMG

HC963_0046_SR2.IMG

HC963_0047_SR2.IMG

HC963_0048_SR2.IMG

HC963_0049_SR2.IMG

HC963_0050_SR2.IMG

HC963_0051_SR2.IMG

HC963_0052_SR2.IMG

HC963_0053_SR2.IMG

HC963_0054_SR2.IMG

HC963_0055_SR2.IMG

HC963_0056_SR2.IMG

HC963_0057_SR2.IMG

HC963_0058_SR2.IMG

HC963_0059_SR2.IMG

HC963_0060_SR2.IMG

HC963_0061_SR2.IMG

HC963_0062_SR2.IMG

HC963_0063_SR2.IMG

HC963_0064_SR2.IMG

HC963_0065_SR2.IMG

HC963_0066_SR2.IMG

HC963_0067_SR2.IMG

HC963_0068_SR2.IMG

HC963_0069_SR2.IMG

HC963_0070_SR2.IMG

HC963_0071_SR2.IMG

HC963_0072_SR2.IMG

HC963_0073_SR2.IMG

HC963_0074_SR2.IMG

HC963_0075_SR2.IMG

HC963_0076_SR2.IMG

HC963_0077_SR2.IMG

HC963_0078_SR2.IMG

HC963_0079_SR2.IMG

HC963_0080_SR2.IMG

HC963_0081_SR2.IMG

HC963_0082_SR2.IMG

HC963_0083_SR2.IMG

HC963_0084_SR2.IMG

HC963_0085_SR2.IMG

HC963_0086_SR2.IMG

HC963_0087_SR2.IMG

HC963_0088_SR2.IMG

HC963_0089_SR2.IMG

HC963_0090_SR2.IMG

HC963_0091_SR2.IMG

HC963_0092_SR2.IMG

HC963_0093_SR2.IMG

HC963_0094_SR2.IMG

HC963_0095_SR2.IMG

HC963_0096_SR2.IMG

HC963_0097_SR2.IMG

HC975_0003_SR2.IMG

HC975_0004_SR2.IMG

HC975_0005_SR2.IMG

HC975_0006_SR2.IMG

HC975_0007_SR2.IMG

HD010_0003_SR2.IMG

HD010_0004_SR2.IMG

HD010_0005_SR2.IMG

HD010_0006_SR2.IMG

HD021_0003_SR2.IMG

HD021_0004_SR2.IMG

HD021_0005_SR2.IMG

HD021_0006_SR2.IMG

HD021_0007_SR2.IMG

HD090_0020_SR2.IMG

HD090_0021_SR2.IMG

HD090_0022_SR2.IMG

HD090_0023_SR2.IMG

HD090_0024_SR2.IMG

HD090_0025_SR2.IMG

HD090_0026_SR2.IMG

HD090_0027_SR2.IMG

HD090_0028_SR2.IMG

HD090_0029_SR2.IMG

HD090_0030_SR2.IMG

HD090_0031_SR2.IMG

HD090_0032_SR2.IMG

HD090_0033_SR2.IMG

HD090_0034_SR2.IMG

HD090_0035_SR2.IMG

HD090_0036_SR2.IMG

HD090_0037_SR2.IMG

HD090_0038_SR2.IMG

HD090_0039_SR2.IMG

HD090_0040_SR2.IMG

HD090_0041_SR2.IMG

HD090_0042_SR2.IMG

HD090_0043_SR2.IMG

HD090_0044_SR2.IMG

HD090_0045_SR2.IMG

HD090_0046_SR2.IMG

HD090_0047_SR2.IMG

HD090_0048_SR2.IMG

HD090_0049_SR2.IMG

HD090_0050_SR2.IMG

HD090_0051_SR2.IMG

HD090_0052_SR2.IMG

HD090_0053_SR2.IMG

HD090_0054_SR2.IMG

HD090_0055_SR2.IMG

HD090_0056_SR2.IMG

HD090_0057_SR2.IMG

HD090_0058_SR2.IMG

HD090_0059_SR2.IMG

HD090_0060_SR2.IMG

HD090_0061_SR2.IMG

HD090_0062_SR2.IMG

HD090_0063_SR2.IMG

HD090_0064_SR2.IMG

HD090_0065_SR2.IMG

HD090_0066_SR2.IMG

HD090_0067_SR2.IMG

HD090_0068_SR2.IMG

HD090_0069_SR2.IMG

HD090_0070_SR2.IMG

HD090_0071_SR2.IMG

HD090_0072_SR2.IMG

HD098_0002_SR2.IMG

HD098_0003_SR2.IMG

HD098_0004_SR2.IMG

HD098_0005_SR2.IMG

HD121_0002_SR2.IMG

HD121_0003_SR2.IMG

HD121_0004_SR2.IMG

HD121_0005_SR2.IMG

HD121_0006_SR2.IMG

HD121_0007_SR2.IMG

HD144_0005_SR2.IMG

HD144_0006_SR2.IMG

HD144_0007_SR2.IMG

HD159_0004_SR2.IMG

HD159_0005_SR2.IMG

HD159_0006_SR2.IMG

HD159_0007_SR2.IMG

HD167_0004_SR2.IMG

HD167_0005_SR2.IMG

HD167_0006_SR2.IMG

HD167_0007_SR2.IMG

HD179_0003_SR2.IMG

HD179_0004_SR2.IMG

HD179_0005_SR2.IMG

HD179_0006_SR2.IMG

HD202_0002_SR2.IMG

HD202_0003_SR2.IMG

HD202_0004_SR2.IMG

HD202_0005_SR2.IMG

HD202_0006_SR2.IMG

HD202_0007_SR2.IMG

HD213_0005_SR2.IMG

HD213_0006_SR2.IMG

HD213_0007_SR2.IMG

HD225_0002_SR2.IMG

HD225_0003_SR2.IMG

HD225_0004_SR2.IMG

HD225_0005_SR2.IMG

HD225_0006_SR2.IMG

HD228_0001_SR2.IMG

HD228_0002_SR2.IMG

HD228_0003_SR2.IMG

HD228_0004_SR2.IMG

HD228_0005_SR2.IMG

HD228_0006_SR2.IMG

HD228_0007_SR2.IMG

HD228_0008_SR2.IMG

HD228_0009_SR2.IMG

HD228_0010_SR2.IMG

HD228_0011_SR2.IMG

HD228_0012_SR2.IMG

HD228_0013_SR2.IMG

HD228_0014_SR2.IMG

HD228_0015_SR2.IMG

HD228_0016_SR2.IMG

HD228_0017_SR2.IMG

HD228_0018_SR2.IMG

HD228_0019_SR2.IMG

HD228_0020_SR2.IMG

HD228_0021_SR2.IMG

HD228_0022_SR2.IMG

HD228_0023_SR2.IMG

HD228_0024_SR2.IMG

HD228_0025_SR2.IMG

HD228_0026_SR2.IMG

HD228_0027_SR2.IMG

HD228_0028_SR2.IMG

HD228_0029_SR2.IMG

HD228_0030_SR2.IMG

HD228_0031_SR2.IMG

HD228_0032_SR2.IMG

HD228_0033_SR2.IMG

HD228_0034_SR2.IMG

HD228_0035_SR2.IMG

HD228_0036_SR2.IMG

HD228_0037_SR2.IMG

HD228_0038_SR2.IMG

HD228_0039_SR2.IMG

HD228_0040_SR2.IMG

HD228_0041_SR2.IMG

HD228_0042_SR2.IMG

HD228_0043_SR2.IMG

HD228_0044_SR2.IMG

HD228_0045_SR2.IMG

HD228_0046_SR2.IMG

HD228_0047_SR2.IMG

HD228_0048_SR2.IMG

HD228_0049_SR2.IMG

HD228_0050_SR2.IMG

HD228_0051_SR2.IMG

HD228_0052_SR2.IMG

HD228_0053_SR2.IMG

HD228_0054_SR2.IMG

HD228_0055_SR2.IMG

HD228_0056_SR2.IMG

HD228_0057_SR2.IMG

HD228_0058_SR2.IMG

HD228_0059_SR2.IMG

HD228_0060_SR2.IMG

HD228_0061_SR2.IMG

HD228_0062_SR2.IMG

HD228_0063_SR2.IMG

HD228_0064_SR2.IMG

HD228_0065_SR2.IMG

HD228_0066_SR2.IMG

HD228_0067_SR2.IMG

HD228_0068_SR2.IMG

HD228_0069_SR2.IMG

HD228_0070_SR2.IMG

HD228_0071_SR2.IMG

HD228_0072_SR2.IMG

HD228_0073_SR2.IMG

HD228_0074_SR2.IMG

HD228_0075_SR2.IMG

HD228_0076_SR2.IMG

HD228_0077_SR2.IMG

HD228_0078_SR2.IMG

HD228_0079_SR2.IMG

HD228_0080_SR2.IMG

HD228_0081_SR2.IMG

HD228_0082_SR2.IMG

HD228_0083_SR2.IMG

HD228_0084_SR2.IMG

HD228_0085_SR2.IMG

HD228_0086_SR2.IMG

HD228_0087_SR2.IMG

HD228_0088_SR2.IMG

HD228_0089_SR2.IMG

HD228_0090_SR2.IMG

HD228_0091_SR2.IMG

HD228_0092_SR2.IMG

HD228_0093_SR2.IMG

HD228_0094_SR2.IMG

HD228_0095_SR2.IMG

HD228_0096_SR2.IMG

HD228_0097_SR2.IMG

HD228_0098_SR2.IMG

HD228_0099_SR2.IMG

HD271_0002_SR2.IMG

HD271_0003_SR2.IMG

HD271_0004_SR2.IMG

HD271_0005_SR2.IMG

HD271_0006_SR2.IMG

HD271_0007_SR2.IMG

HD306_0003_SR2.IMG

HD306_0004_SR2.IMG

HD306_0005_SR2.IMG

HD306_0006_SR2.IMG

HD320_0002_SR2.IMG

HD320_0003_SR2.IMG

HD320_0004_SR2.IMG

HD320_0005_SR2.IMG

HD320_0006_SR2.IMG

HD320_0007_SR2.IMG

HD320_0008_SR2.IMG

HD320_0009_SR2.IMG

HD320_0010_SR2.IMG

HD320_0011_SR2.IMG

HD320_0012_SR2.IMG

HD320_0013_SR2.IMG

HD320_0014_SR2.IMG

HD320_0015_SR2.IMG

HD320_0016_SR2.IMG

HD320_0017_SR2.IMG

HD320_0018_SR2.IMG

HD320_0019_SR2.IMG

HD320_0020_SR2.IMG

HD320_0021_SR2.IMG

HD320_0022_SR2.IMG

HD320_0023_SR2.IMG

HD320_0024_SR2.IMG

HD320_0025_SR2.IMG

HD320_0026_SR2.IMG

HD320_0027_SR2.IMG

HD320_0028_SR2.IMG

HD320_0029_SR2.IMG

HD320_0030_SR2.IMG

HD320_0031_SR2.IMG

HD320_0032_SR2.IMG

HD320_0033_SR2.IMG

HD320_0034_SR2.IMG

HD320_0035_SR2.IMG

HD320_0036_SR2.IMG

HD320_0037_SR2.IMG

HD320_0038_SR2.IMG

HD320_0039_SR2.IMG

HD320_0040_SR2.IMG

HD320_0041_SR2.IMG

HD320_0042_SR2.IMG

HD320_0043_SR2.IMG

HD320_0044_SR2.IMG

HD320_0045_SR2.IMG

HD320_0046_SR2.IMG

HD320_0047_SR2.IMG

HD320_0048_SR2.IMG

HD320_0049_SR2.IMG

HD320_0050_SR2.IMG

HD320_0051_SR2.IMG

HD320_0052_SR2.IMG

HD320_0053_SR2.IMG

HD320_0054_SR2.IMG

HD320_0055_SR2.IMG

HD320_0056_SR2.IMG

HD320_0057_SR2.IMG

HD320_0058_SR2.IMG

HD320_0059_SR2.IMG

HD320_0060_SR2.IMG

HD320_0061_SR2.IMG

HD320_0062_SR2.IMG

HD320_0063_SR2.IMG

HD320_0064_SR2.IMG

HD320_0065_SR2.IMG

HD320_0066_SR2.IMG

HD320_0067_SR2.IMG

HD320_0068_SR2.IMG

HD320_0069_SR2.IMG

HD320_0070_SR2.IMG

HD320_0071_SR2.IMG

HD320_0072_SR2.IMG

HD320_0073_SR2.IMG

HD320_0074_SR2.IMG

HD320_0075_SR2.IMG

HD320_0076_SR2.IMG

HD320_0077_SR2.IMG

HD320_0078_SR2.IMG

HD320_0079_SR2.IMG

HD320_0080_SR2.IMG

HD320_0081_SR2.IMG

HD320_0082_SR2.IMG

HD320_0083_SR2.IMG

HD320_0084_SR2.IMG

HD320_0085_SR2.IMG

HD320_0086_SR2.IMG

HD320_0087_SR2.IMG

HD320_0088_SR2.IMG

HD320_0089_SR2.IMG

HD320_0090_SR2.IMG

HD320_0091_SR2.IMG

HD320_0092_SR2.IMG

HD320_0093_SR2.IMG

HD320_0094_SR2.IMG

HD320_0095_SR2.IMG

HD330_0004_SR2.IMG

HD330_0005_SR2.IMG

HD330_0006_SR2.IMG

HD330_0007_SR2.IMG

HD412_0004_SR2.IMG

HD412_0005_SR2.IMG

HD412_0006_SR2.IMG

HD473_0004_SR2.IMG

HD473_0005_SR2.IMG

HD473_0006_SR2.IMG

HD496_0003_SR2.IMG

HD496_0004_SR2.IMG

HD496_0005_SR2.IMG

HD496_0006_SR2.IMG

HD567_0003_SR2.IMG

HD567_0004_SR2.IMG

HD567_0005_SR2.IMG

HD567_0006_SR2.IMG

HD648_0003_SR2.IMG

HD648_0004_SR2.IMG

HD648_0005_SR2.IMG

HD648_0006_SR2.IMG

HD683_0003_SR2.IMG

HD683_0004_SR2.IMG

HD683_0005_SR2.IMG

HD683_0006_SR2.IMG

HD683_0007_SR2.IMG

HD741_0003_SR2.IMG

HD741_0004_SR2.IMG

HD741_0005_SR2.IMG

HD741_0006_SR2.IMG

HD741_0007_SR2.IMG

HD764_0002_SR2.IMG

HD764_0003_SR2.IMG

HD764_0004_SR2.IMG

HD764_0005_SR2.IMG

HD764_0006_SR2.IMG

HD787_0003_SR2.IMG

HD787_0004_SR2.IMG

HD787_0005_SR2.IMG

HD799_0004_SR2.IMG

HD799_0005_SR2.IMG

HD799_0006_SR2.IMG

HD857_0004_SR2.IMG

HD857_0005_SR2.IMG

HD857_0006_SR2.IMG

HD864_0004_SR2.IMG

HD864_0005_SR2.IMG

HD864_0006_SR2.IMG

HD864_0007_SR2.IMG

HD915_0005_SR2.IMG

HD915_0006_SR2.IMG

HD950_0005_SR2.IMG

HD950_0006_SR2.IMG

HD950_0007_SR2.IMG

HD996_0003_SR2.IMG

HD996_0004_SR2.IMG

HD996_0005_SR2.IMG

HD996_0006_SR2.IMG

HE051_0003_SR2.IMG

HE051_0004_SR2.IMG

HE051_0005_SR2.IMG

HE051_0006_SR2.IMG

HE079_0019_SR2.IMG

HE079_0020_SR2.IMG

HE079_0021_SR2.IMG

HE079_0022_SR2.IMG

HE079_0023_SR2.IMG

HE079_0024_SR2.IMG

HE079_0025_SR2.IMG

HE079_0026_SR2.IMG

HE079_0027_SR2.IMG

HE079_0028_SR2.IMG

HE079_0029_SR2.IMG

HE079_0030_SR2.IMG

HE079_0031_SR2.IMG

HE079_0032_SR2.IMG

HE079_0033_SR2.IMG

HE079_0034_SR2.IMG

HE079_0035_SR2.IMG

HE079_0036_SR2.IMG

HE079_0037_SR2.IMG

HE079_0038_SR2.IMG

HE079_0039_SR2.IMG

HE079_0040_SR2.IMG

HE079_0041_SR2.IMG

HE079_0042_SR2.IMG

HE079_0043_SR2.IMG

HE079_0044_SR2.IMG

HE079_0045_SR2.IMG

HE079_0046_SR2.IMG

HE079_0047_SR2.IMG

HE079_0048_SR2.IMG

HE079_0049_SR2.IMG

HE079_0050_SR2.IMG

HE079_0051_SR2.IMG

HE079_0052_SR2.IMG

HE079_0053_SR2.IMG

HE079_0054_SR2.IMG

HE079_0055_SR2.IMG

HE079_0056_SR2.IMG

HE079_0057_SR2.IMG

HE079_0058_SR2.IMG

HE079_0059_SR2.IMG

HE079_0060_SR2.IMG

HE079_0061_SR2.IMG

HE079_0062_SR2.IMG

HE079_0063_SR2.IMG

HE079_0064_SR2.IMG

HE079_0065_SR2.IMG

HE079_0066_SR2.IMG

HE079_0067_SR2.IMG

HE079_0068_SR2.IMG

HE079_0069_SR2.IMG

HE079_0070_SR2.IMG

HE079_0071_SR2.IMG

HE079_0072_SR2.IMG

HE100_0004_SR2.IMG

HE100_0005_SR2.IMG

HE100_0006_SR2.IMG

HE100_0007_SR2.IMG

HE147_0003_SR2.IMG

HE147_0004_SR2.IMG

HE147_0005_SR2.IMG

HE147_0006_SR2.IMG

HE147_0007_SR2.IMG

HE155_0003_SR2.IMG

HE155_0004_SR2.IMG

HE155_0005_SR2.IMG

HE155_0006_SR2.IMG

HE155_0007_SR2.IMG

HE213_0002_SR2.IMG

HE213_0003_SR2.IMG

HE213_0004_SR2.IMG

HE213_0005_SR2.IMG

HE213_0006_SR2.IMG

HE213_0007_SR2.IMG

HE236_0004_SR2.IMG

HE236_0005_SR2.IMG

HE236_0006_SR2.IMG

HE236_0007_SR2.IMG

HE236_0008_SR2.IMG

HE274_0004_SR2.IMG

HE274_0005_SR2.IMG

HE274_0006_SR2.IMG

HE329_0004_SR2.IMG

HE329_0005_SR2.IMG

HE329_0006_SR2.IMG

HE329_0007_SR2.IMG

HE354_0017_SR2.IMG

HE354_0018_SR2.IMG

HE354_0019_SR2.IMG

HE354_0020_SR2.IMG

HE354_0021_SR2.IMG

HE354_0022_SR2.IMG

HE354_0023_SR2.IMG

HE354_0024_SR2.IMG

HE354_0025_SR2.IMG

HE354_0026_SR2.IMG

HE354_0027_SR2.IMG

HE354_0028_SR2.IMG

HE354_0029_SR2.IMG

HE354_0030_SR2.IMG

HE354_0031_SR2.IMG

HE354_0032_SR2.IMG

HE354_0033_SR2.IMG

HE354_0034_SR2.IMG

HE354_0035_SR2.IMG

HE354_0036_SR2.IMG

HE354_0037_SR2.IMG

HE354_0038_SR2.IMG

HE354_0039_SR2.IMG

HE354_0040_SR2.IMG

HE354_0041_SR2.IMG

HE354_0042_SR2.IMG

HE354_0043_SR2.IMG

HE354_0044_SR2.IMG

HE354_0045_SR2.IMG

HE354_0046_SR2.IMG

HE354_0047_SR2.IMG

HE354_0048_SR2.IMG

HE354_0049_SR2.IMG

HE354_0050_SR2.IMG

HE354_0051_SR2.IMG

HE354_0052_SR2.IMG

HE354_0053_SR2.IMG

HE354_0054_SR2.IMG

HE354_0055_SR2.IMG

HE354_0056_SR2.IMG

HE354_0057_SR2.IMG

HE354_0058_SR2.IMG

HE354_0059_SR2.IMG

HE354_0060_SR2.IMG

HE354_0061_SR2.IMG

HE388_0004_SR2.IMG

HE388_0005_SR2.IMG

HE388_0006_SR2.IMG

HE637_0004_SR2.IMG

HE637_0005_SR2.IMG

HE637_0006_SR2.IMG

HE637_0007_SR2.IMG

HE718_0004_SR2.IMG

HE718_0005_SR2.IMG

HE718_0006_SR2.IMG

HE718_0007_SR2.IMG

HE811_0006_SR2.IMG

HE811_0007_SR2.IMG

HF043_0004_SR2.IMG

HF043_0005_SR2.IMG

HF043_0006_SR2.IMG

HF043_0007_SR2.IMG

HF052_0003_SR2.IMG

HF052_0004_SR2.IMG

HF052_0005_SR2.IMG

HF052_0006_SR2.IMG

HF052_0007_SR2.IMG

HF102_0003_SR2.IMG

HF102_0004_SR2.IMG

HF102_0005_SR2.IMG

HF102_0006_SR2.IMG

HF102_0007_SR2.IMG

HF136_0003_SR2.IMG

HF136_0004_SR2.IMG

HF136_0005_SR2.IMG

HF136_0006_SR2.IMG

HF136_0007_SR2.IMG

HF179_0005_SR2.IMG

HF179_0006_SR2.IMG

HF179_0007_SR2.IMG

HF237_0004_SR2.IMG

HF237_0005_SR2.IMG

HF237_0006_SR2.IMG

HF237_0007_SR2.IMG

HF272_0005_SR2.IMG

HF272_0006_SR2.IMG

HF330_0003_SR2.IMG

HF330_0004_SR2.IMG

HF330_0005_SR2.IMG

HF330_0006_SR2.IMG

HF342_0004_SR2.IMG

HF342_0005_SR2.IMG

HF342_0006_SR2.IMG

HF353_0004_SR2.IMG

HF353_0005_SR2.IMG

HF353_0006_SR2.IMG

HF365_0003_SR2.IMG

HF365_0004_SR2.IMG

HF365_0005_SR2.IMG

HF412_0004_SR2.IMG

HF412_0005_SR2.IMG

HF424_0004_SR2.IMG

HF424_0005_SR2.IMG

HF425_0004_SR2.IMG

HF425_0005_SR2.IMG

HF425_0006_SR2.IMG

HF425_0007_SR2.IMG

HF473_0003_SR2.IMG

HF473_0004_SR2.IMG

HF473_0005_SR2.IMG

HF473_0006_SR2.IMG

HF473_0007_SR2.IMG

HF496_0003_SR2.IMG

HF496_0004_SR2.IMG

HF496_0005_SR2.IMG

HF496_0006_SR2.IMG

HF578_0004_SR2.IMG

HF578_0005_SR2.IMG

HF578_0006_SR2.IMG

HF605_0004_SR2.IMG

HF605_0005_SR2.IMG

HF605_0006_SR2.IMG

HF639_0004_SR2.IMG

HF639_0005_SR2.IMG

HF639_0006_SR2.IMG

HF675_0004_SR2.IMG

HF675_0005_SR2.IMG

HF675_0006_SR2.IMG

HF675_0007_SR2.IMG

HF733_0003_SR2.IMG

HF733_0004_SR2.IMG

HF733_0005_SR2.IMG

HF733_0006_SR2.IMG

HF768_0004_SR2.IMG

HF768_0005_SR2.IMG

HF780_0004_SR2.IMG

HF780_0005_SR2.IMG

HF780_0006_SR2.IMG

HF815_0003_SR2.IMG

HF815_0004_SR2.IMG

HF815_0005_SR2.IMG

HF815_0006_SR2.IMG

HF827_0004_SR2.IMG

HF827_0005_SR2.IMG

HF880_0009_SR2.IMG

HF880_0010_SR2.IMG

HF880_0011_SR2.IMG

HF880_0012_SR2.IMG

HF880_0014_SR2.IMG

HF880_0015_SR2.IMG

HF880_0016_SR2.IMG

HF880_0017_SR2.IMG

HF880_0018_SR2.IMG

HF880_0019_SR2.IMG

HF880_0020_SR2.IMG

HF880_0021_SR2.IMG

HF880_0022_SR2.IMG

HF880_0023_SR2.IMG

HF880_0024_SR2.IMG

HF880_0025_SR2.IMG

HF880_0026_SR2.IMG

HF880_0027_SR2.IMG

HF880_0028_SR2.IMG

HF880_0029_SR2.IMG

HF880_0030_SR2.IMG

HF880_0031_SR2.IMG

HF880_0032_SR2.IMG

HF880_0033_SR2.IMG

HF880_0034_SR2.IMG

HF880_0035_SR2.IMG

HF880_0036_SR2.IMG

HF897_0004_SR2.IMG

HF897_0005_SR2.IMG

HF908_0004_SR2.IMG

HF908_0005_SR2.IMG

HF920_0004_SR2.IMG

HF920_0005_SR2.IMG

HF967_0005_SR2.IMG

**# HRSC (HRSC linescan)**

H0413_0000_S22.IMG

H0413_0000_P22.IMG

H0413_0000_ND2.IMG

H0413_0000_P12.IMG

H0413_0000_S12.IMG

H0649_0000_ND2.IMG

H0649_0000_P12.IMG

H0682_0000_S22.IMG

H0682_0000_P22.IMG

H0682_0000_ND2.IMG

H0682_0000_P12.IMG

H0682_0000_S12.IMG

H0715_0000_S22.IMG

H0715_0000_RE2.IMG

H0715_0000_P22.IMG

H0715_0000_BL2.IMG

H0715_0000_ND2.IMG

H0715_0000_GR2.IMG

H0715_0000_P12.IMG

H0715_0000_IR2.IMG

H0715_0000_S12.IMG

H0748_0001_S22.IMG

H0748_0001_RE2.IMG

H0748_0001_P22.IMG

H0748_0001_BL2.IMG

H0748_0001_ND2.IMG

H0748_0001_GR2.IMG

H0748_0001_P12.IMG

H0748_0001_IR2.IMG

H0748_0001_S12.IMG

H0756_0000_S22.IMG

H0756_0000_RE2.IMG

H0756_0000_P22.IMG

H0756_0000_ND2.IMG

H0756_0000_GR2.IMG

H0756_0000_P12.IMG

H0756_0000_S12.IMG

H1212_0000_S12.IMG

H1212_0000_P12.IMG

H1212_0000_ND2.IMG

H1212_0000_P22.IMG

H1769_0000_RE2.IMG

H1769_0000_S22.IMG

H1901_0009_ND2.IMG

H2151_0000_ND2.IMG

H2233_0000_ND2.IMG

H2233_0000_P12.IMG

H2233_0000_S12.IMG

H2405_0000_P12.IMG

H2446_0000_RE2.IMG

H2446_0000_BL2.IMG

H2446_0000_GR2.IMG

H2446_0000_IR2.IMG

H2487_0000_ND2.IMG

H2643_0000_ND2.IMG

H2673_0000_ND2.IMG

H2673_0000_S12.IMG

H2682_0000_ND2.IMG

H2706_0000_RE2.IMG

H2706_0000_BL2.IMG

H2706_0000_GR2.IMG

H2706_0000_IR2.IMG

H2739_0000_RE2.IMG

H2739_0000_BL2.IMG

H2739_0000_GR2.IMG

H2739_0000_IR2.IMG

H2747_0000_S22.IMG

H2747_0000_P22.IMG

H2747_0000_ND2.IMG

H2747_0000_P12.IMG

H2747_0000_S12.IMG

H2756_0000_BL2.IMG

H2756_0000_GR2.IMG

H2756_0000_IR2.IMG

H2780_0000_S22.IMG

H2780_0000_P22.IMG

H2780_0000_ND2.IMG

H2780_0000_P12.IMG

H2780_0000_S12.IMG

H2805_0018_GR2.IMG

H2805_0018_RE2.IMG

H2813_0000_S12.IMG

H2813_0000_P12.IMG

H2813_0000_ND2.IMG

H2813_0000_P22.IMG

H2846_0001_IR2.IMG

H2846_0001_GR2.IMG

H2846_0001_ND2.IMG

H2846_0001_BL2.IMG

H2846_0001_RE2.IMG

H2854_0000_P12.IMG

H2854_0000_ND2.IMG

H2854_0000_P22.IMG

H2854_0000_S22.IMG

H3310_0000_S12.IMG

H3310_0000_P12.IMG

H3310_0000_ND2.IMG

H3310_0000_P22.IMG

H3310_0000_S22.IMG

H3761_0000_GR2.IMG

H3769_0000_ND2.IMG

H3769_0000_S22.IMG

H3802_0000_S12.IMG

H3802_0000_P12.IMG

H3802_0000_ND2.IMG

H3802_0000_P22.IMG

H3802_0000_S22.IMG

H3835_0000_GR2.IMG

H3843_0000_S22.IMG

H3843_0000_P22.IMG

H3843_0000_ND2.IMG

H3843_0000_P12.IMG

H3843_0000_S12.IMG

H3868_0000_S22.IMG

H3868_0000_P22.IMG

H3868_0000_ND2.IMG

H3868_0000_P12.IMG

H3868_0000_S12.IMG

H3876_0000_RE2.IMG

H3876_0000_BL2.IMG

H3876_0000_GR2.IMG

H3876_0000_IR2.IMG

H3909_0000_S22.IMG

H3909_0000_P22.IMG

H3942_0000_RE2.IMG

H3942_0000_BL2.IMG

H3942_0000_GR2.IMG

H3999_0000_ND2.IMG

H4000_0000_ND2.IMG

H4233_0000_RE2.IMG

H4233_0000_BL2.IMG

H4233_0000_GR2.IMG

H4274_0000_S22.IMG

H4274_0000_P22.IMG

H4274_0000_ND2.IMG

H4274_0000_P12.IMG

H4274_0000_S12.IMG

H4307_0000_S22.IMG

H4307_0000_P22.IMG

H4307_0000_ND2.IMG

H4307_0000_P12.IMG

H4307_0000_S12.IMG

H4332_0000_RE2.IMG

H4332_0000_BL2.IMG

H4332_0000_GR2.IMG

H4332_0000_IR2.IMG

H4340_0000_S22.IMG

H4340_0000_P22.IMG

H4340_0000_ND2.IMG

H4340_0000_P12.IMG

H4340_0000_S12.IMG

H4348_0000_S12.IMG

H4348_0000_P12.IMG

H4348_0000_ND2.IMG

H4373_0000_RE2.IMG

H4373_0000_BL2.IMG

H4373_0000_GR2.IMG

H4381_0000_S12.IMG

H4381_0000_P12.IMG

H4381_0000_ND2.IMG

H4381_0000_P22.IMG

H4381_0000_S22.IMG

H4414_0000_P12.IMG

H4447_0000_GR2.IMG

H4636_0000_ND2.IMG

H4683_0001_ND2.IMG

H4765_0001_BL2.IMG

H4765_0001_ND2.IMG

H4765_0001_GR2.IMG

H4773_0000_S22.IMG

H4773_0000_P22.IMG

H4773_0000_ND2.IMG

H4773_0000_P12.IMG

H4773_0000_S12.IMG

H4806_0001_RE2.IMG

H4806_0001_BL2.IMG

H4806_0001_GR2.IMG

H4806_0001_IR2.IMG

H4814_0000_P22.IMG

H4814_0000_P12.IMG

H4847_0000_S12.IMG

H4847_0000_P12.IMG

H4847_0000_ND2.IMG

H4847_0000_P22.IMG

H4847_0000_S22.IMG

H4855_0000_S12.IMG

H4855_0000_P12.IMG

H4855_0000_ND2.IMG

H4880_0000_GR2.IMG

H4888_0001_S12.IMG

H4888_0001_GR2.IMG

H4888_0001_ND2.IMG

H4888_0001_BL2.IMG

H4888_0001_RE2.IMG

H4888_0001_S22.IMG

H5305_0000_GR2.IMG

H5305_0000_ND2.IMG

H5305_0000_RE2.IMG

H5343_0000_S12.IMG

H5343_0000_P12.IMG

H5343_0000_ND2.IMG

H5343_0000_P22.IMG

H5343_0000_S22.IMG

H5362_0000_IR2.IMG

H5362_0000_GR2.IMG

H5362_0000_ND2.IMG

H5362_0000_BL2.IMG

H5362_0000_RE2.IMG

H5362_0000_S22.IMG

H5381_0000_GR2.IMG

H5381_0000_BL2.IMG

H5381_0000_RE2.IMG

H5409_0000_S22.IMG

H5409_0000_P22.IMG

H5409_0000_ND2.IMG

H5409_0000_P12.IMG

H5409_0000_S12.IMG

H5428_0000_S22.IMG

H5428_0000_RE2.IMG

H5428_0000_BL2.IMG

H5428_0000_ND2.IMG

H5428_0000_GR2.IMG

H5428_0000_IR2.IMG

H5428_0000_S12.IMG

H5447_0000_S22.IMG

H5447_0000_RE2.IMG

H5447_0000_P22.IMG

H5447_0000_BL2.IMG

H5447_0000_ND2.IMG

H5447_0000_GR2.IMG

H5447_0000_P12.IMG

H5447_0000_IR2.IMG

H5447_0000_S12.IMG

H5504_0000_GR2.IMG

H5504_0000_IR2.IMG

H5851_0000_S22.IMG

H5851_0000_P22.IMG

H5851_0000_ND2.IMG

H5851_0000_P12.IMG

H5851_0000_S12.IMG

H5870_0000_S22.IMG

H5870_0000_P22.IMG

H5870_0000_ND2.IMG

H5870_0000_P12.IMG

H5870_0000_S12.IMG

H5889_0000_S22.IMG

H5889_0000_RE2.IMG

H5889_0000_BL2.IMG

H5889_0000_ND2.IMG

H5889_0000_GR2.IMG

H5889_0000_IR2.IMG

H5889_0000_S12.IMG

H5908_0000_RE2.IMG

H5908_0000_BL2.IMG

H5908_0000_GR2.IMG

H5908_0000_IR2.IMG

H5984_0000_S22.IMG

H5984_0000_RE2.IMG

H5984_0000_BL2.IMG

H5984_0000_ND2.IMG

H5984_0000_GR2.IMG

H5984_0000_IR2.IMG

H5984_0000_S12.IMG

H6042_0000_ND2.IMG

H6128_0000_ND2.IMG

H6217_0000_ND2.IMG

H6745_0000_ND2.IMG

H6906_0000_S22.IMG

H6906_0000_P22.IMG

H6906_0000_ND2.IMG

H6906_0000_P12.IMG

H6906_0000_S12.IMG

H6916_0000_S12.IMG

H6916_0000_P12.IMG

H6916_0000_ND2.IMG

H6916_0000_P22.IMG

H6916_0000_S22.IMG

H6926_0000_GR2.IMG

H6926_0000_ND2.IMG

H6926_0000_S22.IMG

H6987_0000_GR2.IMG

H6987_0000_S22.IMG

H7407_0000_S12.IMG

H7407_0000_GR2.IMG

H7407_0000_ND2.IMG

H7407_0000_BL2.IMG

H7407_0000_RE2.IMG

H7407_0000_S22.IMG

H7478_0000_S22.IMG

H7478_0000_P22.IMG

H7478_0000_ND2.IMG

H7478_0000_P12.IMG

H7478_0000_S12.IMG

H7488_0000_S22.IMG

H7488_0000_P22.IMG

H7488_0000_ND2.IMG

H7488_0000_P12.IMG

H7488_0000_S12.IMG

H7915_0009_S22.IMG

H7915_0009_P22.IMG

H7915_0009_ND2.IMG

H7915_0009_P12.IMG

H7915_0009_S12.IMG

H7926_0009_S22.IMG

H7926_0009_P22.IMG

H7926_0009_ND2.IMG

H7926_0009_P12.IMG

H7926_0009_S12.IMG

H7937_0000_S22.IMG

H7937_0000_P22.IMG

H7937_0000_ND2.IMG

H7937_0000_P12.IMG

H7937_0000_S12.IMG

H7948_0000_GR2.IMG

H7959_0000_S22.IMG

H7959_0000_ND2.IMG

H7959_0000_S12.IMG

H7982_0000_S22.IMG

H7982_0000_RE2.IMG

H7982_0000_BL2.IMG

H7982_0000_ND2.IMG

H7982_0000_GR2.IMG

H7982_0000_IR2.IMG

H7982_0000_S12.IMG

H8017_0000_RE2.IMG

H8017_0000_BL2.IMG

H8017_0000_GR2.IMG

H8017_0000_IR2.IMG

H8052_0000_ND2.IMG

H8146_0000_ND2.IMG

H8217_0000_ND2.IMG

H8276_0000_ND2.IMG

H8326_0000_ND2.IMG

H8396_0000_ND2.IMG

H8477_0000_GR2.IMG

H8535_0000_GR2.IMG

H8570_0000_GR2.IMG

H8663_0000_ND2.IMG

H8870_0001_ND2.IMG

H8951_0000_RE2.IMG

H8951_0000_BL2.IMG

H8951_0000_GR2.IMG

H8963_0000_RE2.IMG

H8963_0000_BL2.IMG

H8963_0000_GR2.IMG

H8963_0000_IR2.IMG

H8974_0000_S12.IMG

H8974_0000_P12.IMG

H8974_0000_ND2.IMG

H8974_0000_P22.IMG

H8974_0000_S22.IMG

H8986_0000_RE2.IMG

H8986_0000_BL2.IMG

H8986_0000_GR2.IMG

H8986_0000_IR2.IMG

H8998_0000_ND2.IMG

H9574_0000_S22.IMG

H9574_0000_P22.IMG

H9574_0000_ND2.IMG

H9574_0000_P12.IMG

H9574_0000_S12.IMG

H9586_0000_S12.IMG

H9586_0000_P12.IMG

H9586_0000_GR2.IMG

H9586_0000_ND2.IMG

H9586_0000_BL2.IMG

H9586_0000_RE2.IMG

H9609_0000_GR2.IMG

HA166_0000_ND2.IMG

HA188_0000_ND2.IMG

HA259_0000_ND2.IMG

HA295_0000_ND2.IMG

HA393_0000_ND2.IMG

HA510_0000_ND2.IMG

HA660_0000_GR2.IMG

HA889_0000_ND2.IMG

HA981_0000_S22.IMG

HA981_0000_P22.IMG

HA981_0000_ND2.IMG

HA981_0000_P12.IMG

HA981_0000_S12.IMG

HB062_0000_RE2.IMG

HB062_0000_BL2.IMG

HB062_0000_GR2.IMG

HB062_0000_IR2.IMG

HB085_0000_RE2.IMG

HB085_0000_BL2.IMG

HB085_0000_GR2.IMG

HB085_0000_IR2.IMG

HB108_0000_S12.IMG

HB108_0000_P12.IMG

HB108_0000_ND2.IMG

HB108_0000_P22.IMG

HB108_0000_S22.IMG

HB131_0000_GR2.IMG

HB189_0000_GR2.IMG

HB646_0000_GR2.IMG

HB669_0012_GR2.IMG

HB692_0000_GR2.IMG

HC069_0000_S12.IMG

HC069_0000_GR2.IMG

HC069_0000_ND2.IMG

HC069_0000_S22.IMG

HC092_0000_S12.IMG

HC092_0000_P12.IMG

HC092_0000_P22.IMG

HC092_0000_S22.IMG

HC150_0000_RE2.IMG

HC150_0000_BL2.IMG

HC150_0000_GR2.IMG

HC150_0000_IR2.IMG

HC173_0000_RE2.IMG

HC173_0000_BL2.IMG

HC173_0000_GR2.IMG

HC173_0000_IR2.IMG

HC196_0000_RE2.IMG

HC196_0000_BL2.IMG

HC196_0000_GR2.IMG

HC208_0000_ND2.IMG

HC266_0000_ND2.IMG

HC301_0000_ND2.IMG

HC348_0000_ND2.IMG

HC525_0000_ND2.IMG

HC583_0000_ND2.IMG

HC629_0000_RE2.IMG

HC629_0000_GR2.IMG

HC652_0000_RE2.IMG

HC652_0000_GR2.IMG

HC664_0000_ND2.IMG

HC675_0000_S22.IMG

HC675_0000_P22.IMG

HC675_0000_P12.IMG

HC675_0000_S12.IMG

HC687_0000_ND2.IMG

HC710_0000_RE2.IMG

HC710_0000_BL2.IMG

HC733_0000_RE2.IMG

HC733_0000_BL2.IMG

HC733_0000_GR2.IMG

HC756_0000_RE2.IMG

HC756_0000_BL2.IMG

HC756_0000_GR2.IMG

HC779_0000_RE2.IMG

HC779_0000_BL2.IMG

HC779_0000_GR2.IMG

HC791_0000_ND2.IMG

HC959_0000_ND2.IMG

HC975_0000_ND2.IMG

HD021_0000_ND2.IMG

HD098_0000_RE2.IMG

HD098_0000_BL2.IMG

HD098_0000_GR2.IMG

HD098_0000_IR2.IMG

HD121_0000_RE2.IMG

HD121_0000_BL2.IMG

HD121_0000_GR2.IMG

HD144_0000_S22.IMG

HD144_0000_P22.IMG

HD144_0000_ND2.IMG

HD144_0000_P12.IMG

HD144_0000_S12.IMG

HD167_0000_S22.IMG

HD167_0000_P22.IMG

HD167_0000_ND2.IMG

HD179_0000_S22.IMG

HD179_0000_P22.IMG

HD179_0000_ND2.IMG

HD202_0000_RE2.IMG

HD202_0000_BL2.IMG

HD202_0000_GR2.IMG

HD225_0000_GR2.IMG

HD567_0000_ND2.IMG

HD648_0000_ND2.IMG

HD683_0000_P12.IMG

HD683_0000_ND2.IMG

HD683_0000_P22.IMG

HD741_0000_GR2.IMG

HD764_0000_S22.IMG

HD764_0000_P22.IMG

HD764_0000_ND2.IMG

HD764_0000_P12.IMG

HD764_0000_S12.IMG

HD787_0000_BL2.IMG

HD787_0000_GR2.IMG

HD822_0000_RE2.IMG

HD822_0000_BL2.IMG

HD864_0000_ND2.IMG

HE155_0000_GR2.IMG

HE178_0000_GR2.IMG

HE236_0000_ND2.IMG

HE271_0000_RE2.IMG

HE329_0000_ND2.IMG

HE388_0000_ND2.IMG

HE637_0000_ND2.IMG

HE718_0000_GR2.IMG

HE811_0000_S12.IMG

HE811_0000_ND2.IMG

HE811_0000_P22.IMG

HE811_0000_S22.IMG

HF052_0000_ND2.IMG

HF136_0000_ND2.IMG

HF179_0000_RE2.IMG

HF179_0000_BL2.IMG

HF179_0000_GR2.IMG

HF237_0000_RE2.IMG

HF237_0000_BL2.IMG

HF237_0000_GR2.IMG

HF237_0000_IR2.IMG

HF272_0000_RE2.IMG

HF272_0000_BL2.IMG

HF272_0000_GR2.IMG

HF272_0000_IR2.IMG

HF330_0000_RE2.IMG

HF815_0000_S12.IMG

HF815_0000_P12.IMG

HF815_0000_ND2.IMG

HF815_0000_P22.IMG

HF815_0000_S22.IMG

HF885_0000_GR2.IMG

HF920_0000_ND2.IMG

**# HiRISE**

PSP_007769_9010_BG13_0.IMG

PSP_007769_9010_BG13_1.IMG

PSP_007769_9010_RED5_0.IMG

PSP_007769_9010_RED5_1.IMG

PSP_007769_9010_BG12_0.IMG

PSP_007769_9010_BG12_1.IMG

PSP_007769_9010_RED4_0.IMG

PSP_007769_9010_RED4_1.IMG

PSP_007769_9010_IR11_0.IMG

PSP_007769_9010_IR11_1.IMG

PSP_007769_9010_IR10_0.IMG

PSP_007769_9010_IR10_1.IMG

PSP_007769_9015_BG13_0.IMG

PSP_007769_9015_BG13_1.IMG

PSP_007769_9015_RED5_0.IMG

PSP_007769_9015_RED5_1.IMG

PSP_007769_9015_BG12_0.IMG

PSP_007769_9015_BG12_1.IMG

PSP_007769_9015_RED4_0.IMG

PSP_007769_9015_RED4_1.IMG

PSP_007769_9015_RED6_1.IMG

PSP_007769_9015_IR11_0.IMG

PSP_007769_9015_IR11_1.IMG

PSP_007769_9015_IR10_0.IMG

**Images registered to the Phobos shape model but not used in its construction**

**# Viking**

f034a97.imq

f034a98.imq

f034a99.imq

f126a83.imq

f130a13.imq

f133a31.imq

f136b03.imq

f203a32.imq

f203a34.imq

f244a02.imq

f244a08.imq

f244a09.imq

f304b56.imq

f304b58.imq

f304b78.imq

f304b88.imq

f304b90.imq

f332b02.imq

f334b02.imq

f382a01.imq

f244a68.imq

f246a69.imq

**# Phobos2**

All Phobos2 images were used or considered but not used or registered.

**# MOC**

All MOC images were used or considered but not used or registered.

**# SRC (HRSC framing)**

H7492_0035_SR2.IMG

H7492_0036_SR2.IMG

H7492_0037_SR2.IMG

H7492_0038_SR2.IMG

H7492_0039_SR2.IMG

H7492_0040_SR2.IMG

H7492_0041_SR2.IMG

HC563_0002_SR2.IMG

HC563_0003_SR2.IMG

HC563_0004_SR2.IMG

HC563_0005_SR2.IMG

HC563_0006_SR2.IMG

HC563_0008_SR2.IMG

HC563_0009_SR2.IMG

HC563_0010_SR2.IMG

HC563_0011_SR2.IMG

HC563_0012_SR2.IMG

HC563_0013_SR2.IMG

HC563_0014_SR2.IMG

HC563_0015_SR2.IMG

**# HRSC (HRSC linescan)**

H0413_0000_RE2.IMG

H0413_0000_BL2.IMG

H0413_0000_GR2.IMG

H0413_0000_IR2.IMG

H0649_0000_RE2.IMG

H0649_0000_P22.IMG

H0649_0000_BL2.IMG

H0649_0000_GR2.IMG

H0682_0000_RE2.IMG

H0682_0000_BL2.IMG

H0682_0000_GR2.IMG

H0682_0000_IR2.IMG

H1212_0000_IR2.IMG

H1212_0000_GR2.IMG

H1212_0000_BL2.IMG

H1558_0000_S12.IMG

H1558_0000_IR2.IMG

H1558_0000_P12.IMG

H1558_0000_GR2.IMG

H1558_0000_P22.IMG

H1558_0000_RE2.IMG

H1558_0000_S22.IMG

H1607_0000_S12.IMG

H1607_0000_P12.IMG

H1607_0000_GR2.IMG

H1607_0000_BL2.IMG

H1607_0000_P22.IMG

H1607_0000_RE2.IMG

H1607_0000_S22.IMG

H1901_0009_BL2.IMG

H1901_0009_GR2.IMG

H2151_0000_S22.IMG

H2151_0000_RE2.IMG

H2151_0000_BL2.IMG

H2151_0000_GR2.IMG

H2151_0000_IR2.IMG

H2151_0000_S12.IMG

H2233_0000_BL2.IMG

H2233_0000_GR2.IMG

H2233_0000_IR2.IMG

H2397_0000_RE2.IMG

H2397_0000_BL2.IMG

H2397_0000_GR2.IMG

H2487_0000_S22.IMG

H2487_0000_RE2.IMG

H2487_0000_BL2.IMG

H2487_0000_GR2.IMG

H2487_0000_IR2.IMG

H2487_0000_S12.IMG

H2601_0000_ND2.IMG

H2756_0000_RE2.IMG

H2846_0001_S12.IMG

H2846_0001_P12.IMG

H2846_0001_P22.IMG

H2846_0001_S22.IMG

H2912_0000_S12.IMG

H2912_0000_IR2.IMG

H2912_0000_P12.IMG

H2912_0000_GR2.IMG

H2912_0000_ND2.IMG

H2912_0000_BL2.IMG

H2912_0000_P22.IMG

H2912_0000_RE2.IMG

H2912_0000_S22.IMG

H3245_0000_ND2.IMG

H4030_0000_ND2.IMG

H4348_0000_P22.IMG

H4529_0000_S12.IMG

H4529_0000_IR2.IMG

H4529_0000_P12.IMG

H4529_0000_GR2.IMG

H4529_0000_BL2.IMG

H4529_0000_P22.IMG

H4529_0000_S22.IMG

H4554_0000_ND2.IMG

H4568_0000_ND2.IMG

H4698_0001_ND2.IMG

H4765_0001_P12.IMG

H4765_0001_S12.IMG

H4855_0000_P22.IMG

H4880_0000_RE2.IMG

H4913_0000_S12.IMG

H4913_0000_IR2.IMG

H4913_0000_P12.IMG

H4913_0000_P22.IMG

H4913_0000_RE2.IMG

H4913_0000_S22.IMG

H4946_0000_IR2.IMG

H5305_0000_S12.IMG

H5305_0000_P12.IMG

H5305_0000_P22.IMG

H5305_0000_S22.IMG

H5381_0000_S12.IMG

H5381_0000_P12.IMG

H5381_0000_P22.IMG

H5381_0000_S22.IMG

H5552_0000_ND2.IMG

H5850_0000_ND2.IMG

H5861_0000_S12.IMG

H5861_0000_P12.IMG

H5861_0000_GR2.IMG

H5861_0000_BL2.IMG

H5861_0000_P22.IMG

H5861_0000_RE2.IMG

H6551_0000_ND2.IMG

H6637_0000_ND2.IMG

H6748_0000_ND2.IMG

H6987_0000_S12.IMG

H7225_0000_ND2.IMG

H7742_0000_ND2.IMG

H7813_0000_ND2.IMG

H7959_0000_RE2.IMG

H7959_0000_P22.IMG

H7959_0000_BL2.IMG

H7959_0000_GR2.IMG

H7959_0000_P12.IMG

H7959_0000_IR2.IMG

H8535_0000_IR2.IMG

H9208_0000_ND2.IMG

H9290_0000_ND2.IMG

H9365_0000_ND2.IMG

H9435_0000_ND2.IMG

H9517_0000_ND2.IMG

H9551_0000_IR2.IMG

H9586_0000_P22.IMG

H9586_0000_S22.IMG

H9738_0000_ND2.IMG

H9828_0000_ND2.IMG

HB330_0000_ND2.IMG

HB399_0000_ND2.IMG

HB438_0000_ND2.IMG

HB554_0000_ND2.IMG

HB908_0000_ND2.IMG

HB940_0000_ND2.IMG

HB963_0000_ND2.IMG

HB992_0000_ND2.IMG

HC383_0000_ND2.IMG

HC808_0000_ND2.IMG

HC825_0000_IR2.IMG

HC901_0000_ND2.IMG

HD010_0000_ND2.IMG

HD159_0000_ND2.IMG

HD306_0000_ND2.IMG

HD330_0000_ND2.IMG

HD412_0000_ND2.IMG

HD473_0000_ND2.IMG

HD496_0000_ND2.IMG

HE147_0000_ND2.IMG

HE274_0000_ND2.IMG

HF102_0000_ND2.IMG

HF412_0000_ND2.IMG

HF473_0000_ND2.IMG

HF578_0000_ND2.IMG

HF880_0013_ND2.IMG

HF967_0000_S12.IMG

HF967_0000_P12.IMG

HF967_0000_P22.IMG

HF967_0000_S22.IMG

**# HiRISE**

PSP_007769_9015_IR10_1.IMG

**Images considered but not used or registered**

**# Viking**

f039b80.imq

f039b81.imq

f065a32.imq

f111a03.imq

f203a11.imq

f203a13.imq

f203a19.imq

f203a36.imq

f209a44.imq

f242a02.imq

f242a16.imq

f243a07.imq

f243a09.imq

f243a54.imq

f244a52.imq

f244a54.imq

f246a01.imq

f246a02.imq

f246a10.imq

f246a53.imq

f246a71.imq

f248a07.imq

f249a07.imq

f250a05.imq

f250a07.imq

f250a08.imq

f250a57.imq

f252a11.imq

f252a14.imq

f252a57.imq

f314a01.imq

f314a03.imq

f314a05.imq

f314a07.imq

f314a09.imq

f315a14.imq

f315a32.imq

f329a87.imq

f332b04.imq

f343a06.imq

f343a28.imq

f343a34.imq

f357a10.imq

f357a11.imq

f357a12.imq

f357a13.imq

f357a14.imq

f357a15.imq

f357a33.imq

f357a36.imq

f357a37.imq

f371a09.imq

f371a42.imq

f371a43.imq

f390a05.imq

f390a10.imq

f450a11.imq

f450a13.imq

f488a81.imq

f488a83.imq

f718a53.imq

f718a54.imq

f718a55.imq

f794a53.imq

f794a54.imq

f794a56.imq

f831a56.imq

f831a57.imq

f849a61.imq

f849a62.imq

f849a82.imq

f466a01.imq

f466a02.imq

f466a04.imq

f466a15.imq

f466a16.imq

f466a18.imq

f244a68.imq

f246a69.imq

**#Phobos2**

c2230011.img

c2230022.img

c2230033.img

c2230041.img

c2230063.img

c2230071.img

c2230082.img

c2300021.img

c2300033.img

c2300051.img

c2300063.img

c2300081.img

c2300093.img

c2300102.img

c2300111.img

c2300123.img

c2300132.img

c2300141.img

c2300153.img

c2550021.img

c2550033.img

c2550051.img

c2550063.img

c2550072.img

c2550081.img

c2550093.img

c2550102.img

c2550111.img

c2550141.img

c2550153.img

**#MOC**

SP255103.img

R0600044.img

**#SRC (HRSC framing)**

H0649_0004_SR2.IMG

H1163_0005_SR2.IMG

H1558_0006_SR2.IMG

H2381_0004_SR2.IMG

H2405_0002_SR2.IMG

H2405_0003_SR2.IMG

H2446_0006_SR2.IMG

H2463_0006_SR2.IMG

H2706_0006_SR2.IMG

H2780_0007_SR2.IMG

H2846_0005_SR2.IMG

H2854_0006_SR2.IMG

H2912_0007_SR2.IMG

H2979_0005_SR2.IMG

H3868_0006_SR2.IMG

H3909_0006_SR2.IMG

H3942_0006_SR2.IMG

H4233_0003_SR2.IMG

H4274_0006_SR2.IMG

H4348_0005_SR2.IMG

H4381_0003_SR2.IMG

H4529_0004_SR2.IMG

H4529_0005_SR2.IMG

H4554_0006_SR2.IMG

H4568_0004_SR2.IMG

H4568_0005_SR2.IMG

H4568_0006_SR2.IMG

H4568_0007_SR2.IMG

H4603_0004_SR2.IMG

H4636_0006_SR2.IMG

H4683_0004_SR2.IMG

H4698_0005_SR2.IMG

H4698_0006_SR2.IMG

H4698_0007_SR2.IMG

H4773_0006_SR2.IMG

H4847_0003_SR2.IMG

H4855_0003_SR2.IMG

H4880_0006_SR2.IMG

H5305_0005_SR2.IMG

H5305_0006_SR2.IMG

H5305_0007_SR2.IMG

H5381_0002_SR2.IMG

H5409_0002_SR2.IMG

H5409_0003_SR2.IMG

H5409_0005_SR2.IMG

H5409_0006_SR2.IMG

H5447_0003_SR2.IMG

H5604_0005_SR2.IMG

H5604_0006_SR2.IMG

H5699_0003_SR2.IMG

H5699_0004_SR2.IMG

H5699_0005_SR2.IMG

H5850_0003_SR2.IMG

H5851_0002_SR2.IMG

H5851_0003_SR2.IMG

H5851_0004_SR2.IMG

H5861_0006_SR2.IMG

H5908_0003_SR2.IMG

H5984_0006_SR2.IMG

H6042_0003_SR2.IMG

H6217_0002_SR2.IMG

H6637_0003_SR2.IMG

H6637_0006_SR2.IMG

H6745_0003_SR2.IMG

H6745_0006_SR2.IMG

H6896_0021_SR2.IMG

H6896_0022_SR2.IMG

H6896_0023_SR2.IMG

H6896_0024_SR2.IMG

H6896_0025_SR2.IMG

H6896_0026_SR2.IMG

H6906_0004_SR2.IMG

H6906_0007_SR2.IMG

H6916_0007_SR2.IMG

H6926_0004_SR2.IMG

H6926_0007_SR2.IMG

H7038_0004_SR2.IMG

H7038_0005_SR2.IMG

H7038_0006_SR2.IMG

H7048_0004_SR2.IMG

H7048_0007_SR2.IMG

H7225_0007_SR2.IMG

H7492_0033_SR2.IMG

H7492_0034_SR2.IMG

H7492_0079_SR2.IMG

H7492_0080_SR2.IMG

H7492_0081_SR2.IMG

H7492_0082_SR2.IMG

H7492_0083_SR2.IMG

H7492_0084_SR2.IMG

H7492_0085_SR2.IMG

H7719_0004_SR2.IMG

H7872_0002_SR2.IMG

H7872_0006_SR2.IMG

H7926_0012_SR2.IMG

H7948_0002_SR2.IMG

H7959_0003_SR2.IMG

H8017_0003_SR2.IMG

H8193_0003_SR2.IMG

H8217_0003_SR2.IMG

H8396_0003_SR2.IMG

H8535_0003_SR2.IMG

H8746_0007_SR2.IMG

H8974_0002_SR2.IMG

H8974_0003_SR2.IMG

H8974_0004_SR2.IMG

H8974_0005_SR2.IMG

H8974_0006_SR2.IMG

H8974_0007_SR2.IMG

H8974_0008_SR2.IMG

H8986_0006_SR2.IMG

H9290_0003_SR2.IMG

H9365_0003_SR2.IMG

H9365_0007_SR2.IMG

H9435_0003_SR2.IMG

H9435_0007_SR2.IMG

H9463_0019_SR2.IMG

H9463_0020_SR2.IMG

H9463_0088_SR2.IMG

H9463_0089_SR2.IMG

H9463_0090_SR2.IMG

H9463_0091_SR2.IMG

H9463_0092_SR2.IMG

H9463_0093_SR2.IMG

H9517_0007_SR2.IMG

H9551_0003_SR2.IMG

H9574_0003_SR2.IMG

H9586_0003_SR2.IMG

H9586_0006_SR2.IMG

H9609_0003_SR2.IMG

HB908_0006_SR2.IMG

HB940_0003_SR2.IMG

HB963_0003_SR2.IMG

HC069_0007_SR2.IMG

HC092_0004_SR2.IMG

HC092_0005_SR2.IMG

HC092_0010_SR2.IMG

HC092_0011_SR2.IMG

HC103_0004_SR2.IMG

HC103_0007_SR2.IMG

HC146_0011_SR2.IMG

HC146_0012_SR2.IMG

HC146_0013_SR2.IMG

HC146_0014_SR2.IMG

HC146_0015_SR2.IMG

HC146_0064_SR2.IMG

HC146_0065_SR2.IMG

HC150_0003_SR2.IMG

HC151_0013_SR2.IMG

HC151_0014_SR2.IMG

HC151_0015_SR2.IMG

HC151_0016_SR2.IMG

HC151_0017_SR2.IMG

HC151_0018_SR2.IMG

HC151_0019_SR2.IMG

HC151_0020_SR2.IMG

HC151_0021_SR2.IMG

HC151_0120_SR2.IMG

HC151_0121_SR2.IMG

HC173_0003_SR2.IMG

HC173_0007_SR2.IMG

HC196_0006_SR2.IMG

HC266_0007_SR2.IMG

HC279_0016_SR2.IMG

HC279_0017_SR2.IMG

HC279_0018_SR2.IMG

HC279_0019_SR2.IMG

HC279_0020_SR2.IMG

HC279_0021_SR2.IMG

HC279_0085_SR2.IMG

HC279_0086_SR2.IMG

HC279_0087_SR2.IMG

HC279_0088_SR2.IMG

HC301_0002_SR2.IMG

HC348_0007_SR2.IMG

HC491_0007_SR2.IMG

HC525_0003_SR2.IMG

HC525_0007_SR2.IMG

HC545_0001_SR2.IMG

HC545_0002_SR2.IMG

HC545_0003_SR2.IMG

HC545_0004_SR2.IMG

HC545_0005_SR2.IMG

HC545_0006_SR2.IMG

HC545_0007_SR2.IMG

HC545_0008_SR2.IMG

HC545_0009_SR2.IMG

HC545_0010_SR2.IMG

HC545_0012_SR2.IMG

HC545_0013_SR2.IMG

HC545_0014_SR2.IMG

HC545_0015_SR2.IMG

HC545_0016_SR2.IMG

HC545_0017_SR2.IMG

HC545_0018_SR2.IMG

HC545_0019_SR2.IMG

HC545_0020_SR2.IMG

HC545_0021_SR2.IMG

HC545_0022_SR2.IMG

HC545_0023_SR2.IMG

HC545_0024_SR2.IMG

HC545_0025_SR2.IMG

HC545_0026_SR2.IMG

HC545_0027_SR2.IMG

HC545_0028_SR2.IMG

HC545_0029_SR2.IMG

HC545_0030_SR2.IMG

HC545_0031_SR2.IMG

HC545_0032_SR2.IMG

HC545_0033_SR2.IMG

HC545_0034_SR2.IMG

HC545_0035_SR2.IMG

HC545_0036_SR2.IMG

HC545_0037_SR2.IMG

HC545_0038_SR2.IMG

HC545_0039_SR2.IMG

HC545_0040_SR2.IMG

HC545_0041_SR2.IMG

HC545_0042_SR2.IMG

HC545_0043_SR2.IMG

HC545_0044_SR2.IMG

HC545_0045_SR2.IMG

HC545_0046_SR2.IMG

HC545_0047_SR2.IMG

HC545_0048_SR2.IMG

HC545_0049_SR2.IMG

HC545_0050_SR2.IMG

HC545_0051_SR2.IMG

HC545_0052_SR2.IMG

HC545_0053_SR2.IMG

HC545_0054_SR2.IMG

HC545_0055_SR2.IMG

HC563_0000_SR2.IMG

HC563_0001_SR2.IMG

HC563_0016_SR2.IMG

HC583_0003_SR2.IMG

HC583_0006_SR2.IMG

HC583_0007_SR2.IMG

HC598_0001_SR2.IMG

HC598_0002_SR2.IMG

HC598_0003_SR2.IMG

HC598_0004_SR2.IMG

HC598_0005_SR2.IMG

HC598_0006_SR2.IMG

HC598_0008_SR2.IMG

HC598_0009_SR2.IMG

HC598_0010_SR2.IMG

HC598_0011_SR2.IMG

HC598_0012_SR2.IMG

HC598_0013_SR2.IMG

HC598_0014_SR2.IMG

HC598_0015_SR2.IMG

HC598_0016_SR2.IMG

HC606_0002_SR2.IMG

HC606_0003_SR2.IMG

HC606_0006_SR2.IMG

HC629_0004_SR2.IMG

HC652_0003_SR2.IMG

HC675_0003_SR2.IMG

HC710_0004_SR2.IMG

HC721_0005_SR2.IMG

HC733_0005_SR2.IMG

HC744_0005_SR2.IMG

HC744_0006_SR2.IMG

HC791_0002_SR2.IMG

HC808_0003_SR2.IMG

HC825_0003_SR2.IMG

HC825_0004_SR2.IMG

HC825_0005_SR2.IMG

HC836_0002_SR2.IMG

HC836_0006_SR2.IMG

HC837_0002_SR2.IMG

HC901_0003_SR2.IMG

HC917_0003_SR2.IMG

HC959_0003_SR2.IMG

HC963_0010_SR2.IMG

HC963_0011_SR2.IMG

HC963_0012_SR2.IMG

HC963_0013_SR2.IMG

HC963_0014_SR2.IMG

HC963_0098_SR2.IMG

HC963_0099_SR2.IMG

HC963_0100_SR2.IMG

HC963_0101_SR2.IMG

HD090_0016_SR2.IMG

HD090_0017_SR2.IMG

HD090_0018_SR2.IMG

HD090_0019_SR2.IMG

HD090_0073_SR2.IMG

HD090_0074_SR2.IMG

HD090_0075_SR2.IMG

HD090_0076_SR2.IMG

HD098_0006_SR2.IMG

HD144_0002_SR2.IMG

HD144_0003_SR2.IMG

HD144_0004_SR2.IMG

HD167_0003_SR2.IMG

HD213_0003_SR2.IMG

HD213_0004_SR2.IMG

HD225_0007_SR2.IMG

HD320_0096_SR2.IMG

HD320_0097_SR2.IMG

HD320_0098_SR2.IMG

HD320_0099_SR2.IMG

HD320_0100_SR2.IMG

HD412_0003_SR2.IMG

HD412_0007_SR2.IMG

HD473_0003_SR2.IMG

HD567_0007_SR2.IMG

HD648_0007_SR2.IMG

HD683_0002_SR2.IMG

HD764_0007_SR2.IMG

HD787_0002_SR2.IMG

HD799_0007_SR2.IMG

HD864_0003_SR2.IMG

HE051_0007_SR2.IMG

HE079_0015_SR2.IMG

HE079_0016_SR2.IMG

HE079_0017_SR2.IMG

HE079_0018_SR2.IMG

HE079_0073_SR2.IMG

HE079_0074_SR2.IMG

HE079_0075_SR2.IMG

HE236_0003_SR2.IMG

HE354_0012_SR2.IMG

HE354_0013_SR2.IMG

HE354_0014_SR2.IMG

HE354_0015_SR2.IMG

HE354_0016_SR2.IMG

HE354_0062_SR2.IMG

HE354_0063_SR2.IMG

HE354_0064_SR2.IMG

HE388_0003_SR2.IMG

HE388_0007_SR2.IMG

HE776_0002_SR2.IMG

HE776_0003_SR2.IMG

HE776_0004_SR2.IMG

HE811_0004_SR2.IMG

HE811_0005_SR2.IMG

HF043_0003_SR2.IMG

HF272_0007_SR2.IMG

HF353_0003_SR2.IMG

HF365_0002_SR2.IMG

HF365_0006_SR2.IMG

HF412_0003_SR2.IMG

HF412_0006_SR2.IMG

HF412_0007_SR2.IMG

HF424_0006_SR2.IMG

HF424_0007_SR2.IMG

HF425_0003_SR2.IMG

HF496_0007_SR2.IMG

HF565_0003_SR2.IMG

HF565_0004_SR2.IMG

HF565_0005_SR2.IMG

HF565_0006_SR2.IMG

HF565_0007_SR2.IMG

HF578_0007_SR2.IMG

HF605_0003_SR2.IMG

HF605_0007_SR2.IMG

HF639_0003_SR2.IMG

HF639_0007_SR2.IMG

HF675_0003_SR2.IMG

HF768_0006_SR2.IMG

HF768_0007_SR2.IMG

HF880_0006_SR2.IMG

HF880_0007_SR2.IMG

HF880_0008_SR2.IMG

HF880_0037_SR2.IMG

HF880_0038_SR2.IMG

HF880_0039_SR2.IMG

HF897_0006_SR2.IMG

HF908_0006_SR2.IMG

HF920_0006_SR2.IMG

HF967_0004_SR2.IMG

HF967_0006_SR2.IMG

**#HRSC (HRSC linescan)**

H0649_0000_S22.IMG

H0649_0000_IR2.IMG

H0649_0000_S12.IMG

H0756_0000_BL2.IMG

H0756_0000_IR2.IMG

H1064_0000_ND2.IMG

H1163_0000_ND2.IMG

H1558_0000_ND2.IMG

H1558_0000_BL2.IMG

H1574_0000_ND2.IMG

H1607_0000_IR2.IMG

H1607_0000_ND2.IMG

H2192_0000_S12.IMG

H2397_0000_S22.IMG

H2397_0000_P22.IMG

H2397_0000_ND2.IMG

H2405_0000_ND2.IMG

H2463_0000_ND2.IMG

H2479_0000_ND2.IMG

H2501_0001_ND2.IMG

H2583_0001_ND2.IMG

H2673_0000_S22.IMG

H2673_0000_P22.IMG

H2673_0000_P12.IMG

H2805_0018_IR2.IMG

H2805_0018_BL2.IMG

H2813_0000_S22.IMG

H2854_0000_S12.IMG

H2979_0001_ND2.IMG

H3005_0000_ND2.IMG

H3761_0000_IR2.IMG

H3761_0000_BL2.IMG

H3761_0000_RE2.IMG

H3769_0000_S12.IMG

H3769_0000_P12.IMG

H3769_0000_P22.IMG

H3835_0000_IR2.IMG

H3835_0000_BL2.IMG

H3835_0000_RE2.IMG

H3909_0000_ND2.IMG

H3909_0000_P12.IMG

H3909_0000_S12.IMG

H3942_0000_IR2.IMG

H4233_0000_IR2.IMG

H4348_0000_S22.IMG

H4373_0000_IR2.IMG

H4414_0000_S12.IMG

H4414_0000_ND2.IMG

H4414_0000_P22.IMG

H4414_0000_S22.IMG

H4447_0000_IR2.IMG

H4447_0000_BL2.IMG

H4447_0000_RE2.IMG

H4529_0000_ND2.IMG

H4529_0000_RE2.IMG

H4603_0000_ND2.IMG

H4765_0001_S22.IMG

H4765_0001_RE2.IMG

H4765_0001_P22.IMG

H4765_0001_IR2.IMG

H4814_0000_S22.IMG

H4814_0000_ND2.IMG

H4814_0000_S12.IMG

H4855_0000_S22.IMG

H4880_0000_IR2.IMG

H4880_0000_BL2.IMG

H4888_0001_IR2.IMG

H4913_0000_GR2.IMG

H4913_0000_ND2.IMG

H4913_0000_BL2.IMG

H4946_0000_GR2.IMG

H4946_0000_BL2.IMG

H4946_0000_RE2.IMG

H5163_0000_ND2.IMG

H5277_0000_ND2.IMG

H5305_0000_IR2.IMG

H5305_0000_BL2.IMG

H5362_0000_S12.IMG

H5381_0000_IR2.IMG

H5381_0000_ND2.IMG

H5504_0000_RE2.IMG

H5504_0000_BL2.IMG

H5604_0000_ND2.IMG

H5699_0000_ND2.IMG

H5766_0000_ND2.IMG

H5861_0000_IR2.IMG

H5861_0000_ND2.IMG

H5861_0000_S22.IMG

H6757_0000_ND2.IMG

H6896_0000_ND2.IMG

H6926_0000_S12.IMG

H6926_0000_IR2.IMG

H6926_0000_BL2.IMG

H6926_0000_RE2.IMG

H6987_0000_IR2.IMG

H6987_0000_ND2.IMG

H6987_0000_BL2.IMG

H6987_0000_RE2.IMG

H7017_0000_ND2.IMG

H7038_0000_ND2.IMG

H7048_0000_ND2.IMG

H7088_0000_ND2.IMG

H7109_0001_ND2.IMG

H7407_0000_IR2.IMG

H7492_0005_ND2.IMG

H7800_0000_ND2.IMG

H7872_0000_ND2.IMG

H7948_0000_RE2.IMG

H7948_0000_BL2.IMG

H7948_0000_IR2.IMG

H8088_0000_ND2.IMG

H8193_0000_ND2.IMG

H8477_0000_RE2.IMG

H8477_0000_BL2.IMG

H8477_0000_IR2.IMG

H8512_0000_IR2.IMG

H8535_0000_BL2.IMG

H8535_0000_RE2.IMG

H8570_0000_IR2.IMG

H8570_0000_BL2.IMG

H8570_0000_RE2.IMG

H8746_0000_ND2.IMG

H8951_0000_IR2.IMG

H9161_0000_ND2.IMG

H9463_0028_ND2.IMG

H9551_0000_GR2.IMG

H9551_0000_BL2.IMG

H9551_0000_RE2.IMG

H9586_0000_IR2.IMG

H9609_0000_RE2.IMG

H9609_0000_BL2.IMG

H9609_0000_IR2.IMG

H9909_0000_ND2.IMG

H9921_0000_ND2.IMG

HA579_0000_RE2.IMG

HA579_0000_BL2.IMG

HA579_0000_GR2.IMG

HA579_0000_IR2.IMG

HA602_0000_RE2.IMG

HA602_0000_BL2.IMG

HA602_0000_GR2.IMG

HA602_0000_IR2.IMG

HA625_0000_IR2.IMG

HA625_0000_GR2.IMG

HA625_0000_BL2.IMG

HA625_0000_RE2.IMG

HA660_0000_IR2.IMG

HA660_0000_BL2.IMG

HA660_0000_RE2.IMG

HB027_0000_RE2.IMG

HB027_0000_BL2.IMG

HB027_0000_GR2.IMG

HB027_0000_IR2.IMG

HB131_0000_IR2.IMG

HB131_0000_BL2.IMG

HB131_0000_RE2.IMG

HB143_0000_IR2.IMG

HB143_0000_GR2.IMG

HB143_0000_BL2.IMG

HB143_0000_RE2.IMG

HB189_0000_IR2.IMG

HB189_0000_BL2.IMG

HB189_0000_RE2.IMG

HB247_0000_ND2.IMG

HB294_0000_ND2.IMG

HB646_0000_IR2.IMG

HB646_0000_BL2.IMG

HB646_0000_RE2.IMG

HB657_0000_RE2.IMG

HB657_0000_BL2.IMG

HB657_0000_GR2.IMG

HB657_0000_IR2.IMG

HB669_0012_IR2.IMG

HB669_0012_BL2.IMG

HB669_0012_RE2.IMG

HB692_0000_IR2.IMG

HB692_0000_BL2.IMG

HB692_0000_RE2.IMG

HB911_0000_ND2.IMG

HC046_0000_IR2.IMG

HC046_0000_GR2.IMG

HC046_0000_BL2.IMG

HC046_0000_RE2.IMG

HC069_0000_IR2.IMG

HC069_0000_BL2.IMG

HC069_0000_RE2.IMG

HC092_0000_ND2.IMG

HC103_0000_ND2.IMG

HC127_0000_IR2.IMG

HC127_0000_GR2.IMG

HC127_0000_BL2.IMG

HC127_0000_RE2.IMG

HC146_0004_ND2.IMG

HC151_0032_ND2.IMG

HC196_0000_IR2.IMG

HC491_0000_ND2.IMG

HC563_0007_ND2.IMG

HC598_0007_ND2.IMG

HC606_0000_ND2.IMG

HC629_0000_BL2.IMG

HC629_0000_IR2.IMG

HC652_0000_BL2.IMG

HC652_0000_IR2.IMG

HC675_0000_ND2.IMG

HC710_0000_GR2.IMG

HC710_0000_IR2.IMG

HC721_0000_IR2.IMG

HC721_0000_GR2.IMG

HC721_0000_BL2.IMG

HC721_0000_RE2.IMG

HC733_0000_IR2.IMG

HC744_0000_IR2.IMG

HC744_0000_GR2.IMG

HC744_0000_BL2.IMG

HC744_0000_RE2.IMG

HC756_0000_IR2.IMG

HC779_0000_IR2.IMG

HC802_0000_IR2.IMG

HC802_0000_GR2.IMG

HC802_0000_BL2.IMG

HC802_0000_RE2.IMG

HC825_0000_GR2.IMG

HC825_0000_BL2.IMG

HC825_0000_RE2.IMG

HC836_0000_ND2.IMG

HC837_0000_ND2.IMG

HC871_0000_ND2.IMG

HC917_0000_ND2.IMG

HC928_0000_ND2.IMG

HD121_0000_IR2.IMG

HD202_0000_IR2.IMG

HD213_0000_IR2.IMG

HD213_0000_GR2.IMG

HD213_0000_BL2.IMG

HD225_0000_IR2.IMG

HD225_0000_BL2.IMG

HD225_0000_RE2.IMG

HD248_0000_IR2.IMG

HD271_0000_IR2.IMG

HD271_0000_GR2.IMG

HD271_0000_BL2.IMG

HD271_0000_RE2.IMG

HD683_0000_S12.IMG

HD683_0000_S22.IMG

HD741_0000_IR2.IMG

HD741_0000_BL2.IMG

HD741_0000_RE2.IMG

HD787_0000_RE2.IMG

HD787_0000_IR2.IMG

HD799_0000_IR2.IMG

HD799_0000_GR2.IMG

HD799_0000_BL2.IMG

HD799_0000_RE2.IMG

HD857_0000_IR2.IMG

HD857_0000_GR2.IMG

HD857_0000_BL2.IMG

HD857_0000_RE2.IMG

HD915_0000_ND2.IMG

HD950_0000_ND2.IMG

HD996_0000_ND2.IMG

HE051_0000_ND2.IMG

HE100_0000_ND2.IMG

HE155_0000_IR2.IMG

HE155_0000_BL2.IMG

HE155_0000_RE2.IMG

HE178_0000_IR2.IMG

HE178_0000_BL2.IMG

HE178_0000_RE2.IMG

HE213_0000_IR2.IMG

HE213_0000_GR2.IMG

HE213_0000_BL2.IMG

HE213_0000_RE2.IMG

HE718_0000_RE2.IMG

HE718_0000_BL2.IMG

HE718_0000_IR2.IMG

HE776_0000_IR2.IMG

HE776_0000_GR2.IMG

HE776_0000_BL2.IMG

HE776_0000_RE2.IMG

HE811_0000_P12.IMG

HF043_0000_ND2.IMG

HF179_0000_IR2.IMG

HF202_0000_RE2.IMG

HF202_0000_BL2.IMG

HF330_0000_IR2.IMG

HF330_0000_GR2.IMG

HF330_0000_BL2.IMG

HF342_0000_IR2.IMG

HF342_0000_GR2.IMG

HF342_0000_BL2.IMG

HF342_0000_RE2.IMG

HF353_0000_IR2.IMG

HF353_0000_GR2.IMG

HF353_0000_ND2.IMG

HF353_0000_BL2.IMG

HF353_0000_RE2.IMG

HF365_0000_IR2.IMG

HF365_0000_GR2.IMG

HF365_0000_BL2.IMG

HF365_0000_RE2.IMG

HF425_0000_ND2.IMG

HF496_0000_ND2.IMG

HF565_0000_ND2.IMG

HF639_0000_ND2.IMG

HF675_0000_ND2.IMG

HF733_0000_IR2.IMG

HF733_0000_GR2.IMG

HF733_0000_BL2.IMG

HF733_0000_RE2.IMG

HF768_0000_IR2.IMG

HF768_0000_GR2.IMG

HF768_0000_BL2.IMG

HF768_0000_RE2.IMG

HF780_0000_IR2.IMG

HF780_0000_GR2.IMG

HF780_0000_BL2.IMG

HF780_0000_RE2.IMG

HF827_0000_IR2.IMG

HF827_0000_GR2.IMG

HF827_0000_BL2.IMG

HF827_0000_RE2.IMG

HF885_0000_RE2.IMG

HF885_0000_BL2.IMG

HF885_0000_IR2.IMG

HF897_0000_IR2.IMG

HF897_0000_GR2.IMG

HF897_0000_BL2.IMG

HF897_0000_RE2.IMG

HF967_0000_IR2.IMG

HF967_0000_GR2.IMG

HF967_0000_ND2.IMG

HF967_0000_BL2.IMG

HF967_0000_RE2.IMG

**# HiRISE**

None. All HiRISE images were used and/or registered.
