# Supplementary material for: High-resolution shape models of Phobos and Deimos from stereophotoclinometry
Source: Earth Planets Space. 2023 Jun 25;75(1):103. doi: 10.1186/s40623-023-01814-7 (PMC10290967; doi:10.1186/s40623-023-01814-7)
Supplement: Supplementary file 2 — Additional file 2: Deimos Image Lists. [file 40623_2023_1814_MOESM2_ESM.docx]

**Additional File 2: Deimos Image Lists**

This file contains three lists of images:

1. Images used to construct the Deimos shape model

These images were fully incorporated into the model and were used to construct topography. These images will be available in the coregistered SBMT dataset, and smithed spacecraft position and attitude for each image will be available at the PDS (see Table 7).

1. Images registered to the Deimos shape model but not used in its construction

These images had one of the following limitations: the image resolution was such that the body was too small to support more than three SPC landmarks; only part of the body was in the scene, and the extent or orientation of the body was such that the image could not support more than three landmarks; minor artifacts were visible that might affect topography but would not interfere with image interpretation; the image was high-phase such that the body appeared as a crescent; poor SNR; minimal visible surface features. These images will be available in the coregistered SBMT dataset, and smithed spacecraft position and attitude for each image will be available at the PDS (see Table 7).

1. Images considered but not used or registered

These images had one of the following limitations: too small to support more than three SPC landmarks; insufficient SPICE information; artifacts across image; only portion of the body is in the image; only a small portion of the image is lit; very blurry; low SNR; missing data; partial to complete saturation; body was small and part of a long sequence of effectively redundant images. This list is supplied as a resource for someone wishing to use Deimos images in the future. These images will not be available in the SBMT, nor will smithed spacecraft position and altitude be available at the PDS.

**Images used to construct the Deimos shape model**

**#Viking**

f056a93.imq

f056a95.imq

f056a96.imq

f056a98.imq

f124a87.imq

f124a90.imq

f124a92.imq

f339b02.imq

f339b04.imq

f339b11.imq

f339b13.imq

f339b15.imq

f339b17.imq

f339b19.imq

f339b22.imq

f339b24.imq

f347b02.imq

f347b04.imq

f347b11.imq

f347b13.imq

f347b15.imq

f347b17.imq

f347b19.imq

f355b41.imq

f355b43.imq

f355b45.imq

f355b47.imq

f355b49.imq

f355b51.imq

f355b53.imq

f355b55.imq

f355b57.imq

f355b59.imq

f371b41.imq

f371b43.imq

f371b61.imq

f371b63.imq

f371b65.imq

f371b67.imq

f371b69.imq

f375b61.imq

f375b63.imq

f375b65.imq

f375b81.imq

f375b83.imq

f387b41.imq

f387b43.imq

f387b45.imq

f391b41.imq

f391b43.imq

f391b45.imq

f391b47.imq

f391b49.imq

f395b01.imq

f395b03.imq

f395b05.imq

f395b07.imq

f395b09.imq

f413b81.imq

f413b83.imq

f413b84.imq

f413b85.imq

f413b86.imq

f413b87.imq

f428b21.imq

f428b22.imq

f428b34.imq

f428b35.imq

f428b36.imq

f428b59.imq

f428b60.imq

f428b61.imq

f428b62.imq

f433b31.imq

f433b32.imq

f433b33.imq

f433b34.imq

f433b37.imq

f433b38.imq

f433b39.imq

f433b40.imq

f433b55.imq

f433b56.imq

f433b76.imq

f433b78.imq

f464a03.imq

f464a05.imq

f464a07.imq

f507a01.imq

f512a01.imq

f512a03.imq

f715a03.imq

f715a05.imq

f715a07.imq

f715a09.imq

**#MOC**

s2000602.imq

**#SRC (HRSC framing)**

H0973_0002_SR2.IMG

H0973_0003_SR2.IMG

H0973_0004_SR2.IMG

H1010_0003_SR2.IMG

H1010_0004_SR2.IMG

H1222_0003_SR2.IMG

H1222_0004_SR2.IMG

H1913_0003_SR2.IMG

H1913_0004_SR2.IMG

H1913_0005_SR2.IMG

H1985_0005_SR2.IMG

H1985_0006_SR2.IMG

H2126_0004_SR2.IMG

H2126_0005_SR2.IMG

H2139_0004_SR2.IMG

H2139_0005_SR2.IMG

H2799_0003_SR2.IMG

H2799_0004_SR2.IMG

H2799_0005_SR2.IMG

H2799_0006_SR2.IMG

H2808_0004_SR2.IMG

H2808_0005_SR2.IMG

H2808_0006_SR2.IMG

H2835_0004_SR2.IMG

H2835_0005_SR2.IMG

H2835_0006_SR2.IMG

H2853_0005_SR2.IMG

H2853_0006_SR2.IMG

H2853_0007_SR2.IMG

H2862_0003_SR2.IMG

H2862_0004_SR2.IMG

H2862_0005_SR2.IMG

H2862_0006_SR2.IMG

H2880_0005_SR2.IMG

H2880_0006_SR2.IMG

H2880_0007_SR2.IMG

H2961_0004_SR2.IMG

H2961_0005_SR2.IMG

H2961_0006_SR2.IMG

H2988_0004_SR2.IMG

H2988_0005_SR2.IMG

H2988_0006_SR2.IMG

H3196_0004_SR2.IMG

H3196_0005_SR2.IMG

H3196_0006_SR2.IMG

H3946_0004_SR2.IMG

H3946_0005_SR2.IMG

H3946_0006_SR2.IMG

H4045_0004_SR2.IMG

H4045_0005_SR2.IMG

H4045_0006_SR2.IMG

H4217_0005_SR2.IMG

H4217_0006_SR2.IMG

H4904_0003_SR2.IMG

H4904_0004_SR2.IMG

H4904_0005_SR2.IMG

H4904_0006_SR2.IMG

H4949_0003_SR2.IMG

H4949_0004_SR2.IMG

H4949_0005_SR2.IMG

H4949_0006_SR2.IMG

H4967_0003_SR2.IMG

H4967_0004_SR2.IMG

H4967_0005_SR2.IMG

H4967_0006_SR2.IMG

H5124_0004_SR2.IMG

H5124_0005_SR2.IMG

H5124_0006_SR2.IMG

H5146_0004_SR2.IMG

H5146_0005_SR2.IMG

H5146_0006_SR2.IMG

H5164_0004_SR2.IMG

H5164_0005_SR2.IMG

H5164_0006_SR2.IMG

H5239_0005_SR2.IMG

H5239_0006_SR2.IMG

H5239_0007_SR2.IMG

H5319_0005_SR2.IMG

H5372_0004_SR2.IMG

H5372_0006_SR2.IMG

H5425_0004_SR2.IMG

H6060_0005_SR2.IMG

H6060_0006_SR2.IMG

H6060_0007_SR2.IMG

H6122_0005_SR2.IMG

H6122_0006_SR2.IMG

H6122_0007_SR2.IMG

H6184_0005_SR2.IMG

H6184_0006_SR2.IMG

H6184_0007_SR2.IMG

H6943_0003_SR2.IMG

H6943_0004_SR2.IMG

H6943_0005_SR2.IMG

H6943_0006_SR2.IMG

H6943_0007_SR2.IMG

H7031_0004_SR2.IMG

H7031_0005_SR2.IMG

H7031_0006_SR2.IMG

H7124_0004_SR2.IMG

H7124_0005_SR2.IMG

H7124_0006_SR2.IMG

H7159_0005_SR2.IMG

H7159_0006_SR2.IMG

H7159_0007_SR2.IMG

H7401_0004_SR2.IMG

H7401_0005_SR2.IMG

H7401_0006_SR2.IMG

H8141_0005_SR2.IMG

H8141_0006_SR2.IMG

H8141_0007_SR2.IMG

H8246_0004_SR2.IMG

H8246_0005_SR2.IMG

H8246_0006_SR2.IMG

H8263_0004_SR2.IMG

H8263_0005_SR2.IMG

H8263_0006_SR2.IMG

H8324_0004_SR2.IMG

H8324_0005_SR2.IMG

H8324_0006_SR2.IMG

H9148_0004_SR2.IMG

H9148_0005_SR2.IMG

H9253_0004_SR2.IMG

H9253_0005_SR2.IMG

H9253_0006_SR2.IMG

H9257_0004_SR2.IMG

H9257_0005_SR2.IMG

H9257_0006_SR2.IMG

H9309_0004_SR2.IMG

H9309_0005_SR2.IMG

H9309_0006_SR2.IMG

H9409_0004_SR2.IMG

H9409_0005_SR2.IMG

H9409_0006_SR2.IMG

H9461_0003_SR2.IMG

H9461_0004_SR2.IMG

H9461_0005_SR2.IMG

H9461_0006_SR2.IMG

H9487_0004_SR2.IMG

H9487_0005_SR2.IMG

H9487_0006_SR2.IMG

H9556_0005_SR2.IMG

H9569_0004_SR2.IMG

H9569_0005_SR2.IMG

H9569_0006_SR2.IMG

HC326_0004_SR2.IMG

HC326_0005_SR2.IMG

HC326_0006_SR2.IMG

HC339_0004_SR2.IMG

HC339_0005_SR2.IMG

HC339_0006_SR2.IMG

HC405_0004_SR2.IMG

HC405_0005_SR2.IMG

HC405_0006_SR2.IMG

HC500_0003_SR2.IMG

HC500_0004_SR2.IMG

HC500_0005_SR2.IMG

HC500_0006_SR2.IMG

HC535_0004_SR2.IMG

HC535_0005_SR2.IMG

HC535_0006_SR2.IMG

HC552_0003_SR2.IMG

HC552_0004_SR2.IMG

HC552_0005_SR2.IMG

HC552_0006_SR2.IMG

HC665_0004_SR2.IMG

HC665_0005_SR2.IMG

HC665_0006_SR2.IMG

HC730_0004_SR2.IMG

HC730_0005_SR2.IMG

HC730_0006_SR2.IMG

HD469_0003_SR2.IMG

HD469_0004_SR2.IMG

HD469_0005_SR2.IMG

HD469_0006_SR2.IMG

HD482_0003_SR2.IMG

HD482_0004_SR2.IMG

HD482_0005_SR2.IMG

HD482_0006_SR2.IMG

HD560_0003_SR2.IMG

HD560_0004_SR2.IMG

HD560_0005_SR2.IMG

HD560_0006_SR2.IMG

HD612_0003_SR2.IMG

HD612_0004_SR2.IMG

HD612_0005_SR2.IMG

HD612_0006_SR2.IMG

HD742_0004_SR2.IMG

HD742_0005_SR2.IMG

HD742_0006_SR2.IMG

HE259_0005_SR2.IMG

HE259_0006_SR2.IMG

HE259_0007_SR2.IMG

HE285_0004_SR2.IMG

HE285_0005_SR2.IMG

HE285_0006_SR2.IMG

HE285_0007_SR2.IMG

HE337_0004_SR2.IMG

HE337_0005_SR2.IMG

HE337_0006_SR2.IMG

HE433_0004_SR2.IMG

HE433_0005_SR2.IMG

HE433_0006_SR2.IMG

HE441_0004_SR2.IMG

HE441_0005_SR2.IMG

HE441_0006_SR2.IMG

HE615_0004_SR2.IMG

HE615_0005_SR2.IMG

HE615_0006_SR2.IMG

HE693_0004_SR2.IMG

HE693_0005_SR2.IMG

HE693_0006_SR2.IMG

HF474_0005_SR2.IMG

HF596_0004_SR2.IMG

HF596_0005_SR2.IMG

HF596_0006_SR2.IMG

HF700_0004_SR2.IMG

HF700_0005_SR2.IMG

HF700_0006_SR2.IMG

HF713_0004_SR2.IMG

HF713_0005_SR2.IMG

HF713_0006_SR2.IMG

HF739_0003_SR2.IMG

HF739_0004_SR2.IMG

HF739_0005_SR2.IMG

HF739_0006_SR2.IMG

HF812_0022_SR2.IMG

HF812_0030_SR2.IMG

**# HiRISE**

ESP_012065_9000_BG13_1.IMG

ESP_012065_9000_RED5_1.IMG

ESP_012065_9000_BG12_0.IMG

ESP_012065_9000_RED4_0.IMG

ESP_012065_9000_IR11_1.IMG

ESP_012065_9000_IR10_0.IMG

ESP_012068_9000_BG13_1.IMG

ESP_012068_9000_RED5_1.IMG

ESP_012068_9000_IR11_1.IMG

**Images registered to the Deimos shape model but not used in its construction**

**#Viking**

f423b61.imq

f423b62.imq

f423b63.imq

f464a01.imq

f464a09.imq

f507a03.imq

f507a21.imq

f507a27.imq

f531a01.imq

f531a03.imq

**#MOC**

None. All MOC images were used to construct the model.

**#SRC (HRSC framing)**

H1010_0002_SR2.IMG

H2180_0004_SR2.IMG

H2180_0005_SR2.IMG

H2781_0005_SR2.IMG

H2781_0006_SR2.IMG

H2781_0007_SR2.IMG

H4122_0005_SR2.IMG

H4122_0006_SR2.IMG

H4122_0007_SR2.IMG

H5319_0004_SR2.IMG

H5319_0006_SR2.IMG

H5372_0003_SR2.IMG

H5372_0005_SR2.IMG

H5425_0003_SR2.IMG

H5425_0005_SR2.IMG

H5425_0006_SR2.IMG

H9556_0004_SR2.IMG

H9556_0006_SR2.IMG

HC222_0004_SR2.IMG

HC222_0005_SR2.IMG

HC222_0006_SR2.IMG

HC235_0004_SR2.IMG

HC235_0005_SR2.IMG

HC235_0006_SR2.IMG

HC921_0031_SR2.IMG

HD365_0004_SR2.IMG

HD365_0005_SR2.IMG

HD365_0006_SR2.IMG

HD647_0004_SR2.IMG

HD647_0005_SR2.IMG

HD647_0006_SR2.IMG

HF396_0004_SR2.IMG

HF396_0005_SR2.IMG

HF396_0006_SR2.IMG

HF396_0007_SR2.IMG

HF409_0004_SR2.IMG

HF409_0005_SR2.IMG

HF409_0006_SR2.IMG

HF474_0004_SR2.IMG

HF474_0006_SR2.IMG

HF487_0004_SR2.IMG

HF487_0005_SR2.IMG

HF487_0006_SR2.IMG

HF527_0004_SR2.IMG

HF527_0005_SR2.IMG

HF527_0006_SR2.IMG

**# HiRISE**

None. All HiRISE images were used to construct the model.

**Images considered but not used or registered**

**#Viking**

f371b45.imq

f375b67.imq

f375b85.imq

f387b47.imq

f387b49.imq

f395b11.imq

f413b91.imq

f413b93.imq

f418b62.imq

f427b90.imq

f427b92.imq

f427b98.imq

f428b80.imq

f428b82.imq

f433b41.imq

f433b42.imq

f433b71.imq

f433b72.imq

f433b73.imq

f433b75.imq

f433b77.imq

f507a25.imq

f710a14.imq

f853a41.imq

f853a51.imq

f853a61.imq

f853a71.imq

f853a81.imq

**#MOC**

None. All MOC images were used to construct the model.

**#SRC (HRSC framing)**

H2835_0003_SR2.IMG

H2880_0004_SR2.IMG

H3946_0007_SR2.IMG

H4217_0004_SR2.IMG

H8263_0003_SR2.IMG

H9487_0003_SR2.IMG

H9700_0009_SR2.IMG

H9700_0010_SR2.IMG

H9700_0011_SR2.IMG

H9700_0012_SR2.IMG

H9700_0013_SR2.IMG

H9700_0014_SR2.IMG

H9700_0015_SR2.IMG

H9700_0016_SR2.IMG

H9700_0017_SR2.IMG

H9700_0018_SR2.IMG

H9700_0019_SR2.IMG

H9700_0020_SR2.IMG

H9700_0021_SR2.IMG

H9700_0022_SR2.IMG

H9700_0023_SR2.IMG

H9700_0024_SR2.IMG

H9700_0025_SR2.IMG

H9700_0026_SR2.IMG

H9700_0027_SR2.IMG

H9700_0028_SR2.IMG

H9700_0029_SR2.IMG

H9700_0030_SR2.IMG

H9700_0031_SR2.IMG

H9700_0032_SR2.IMG

H9700_0033_SR2.IMG

H9700_0034_SR2.IMG

H9700_0035_SR2.IMG

H9700_0036_SR2.IMG

H9700_0037_SR2.IMG

H9700_0038_SR2.IMG

H9700_0039_SR2.IMG

H9700_0040_SR2.IMG

H9700_0041_SR2.IMG

H9700_0042_SR2.IMG

H9700_0043_SR2.IMG

H9700_0044_SR2.IMG

H9700_0045_SR2.IMG

H9700_0046_SR2.IMG

H9700_0047_SR2.IMG

H9700_0048_SR2.IMG

H9700_0049_SR2.IMG

H9700_0050_SR2.IMG

H9700_0051_SR2.IMG

H9700_0052_SR2.IMG

H9700_0053_SR2.IMG

H9700_0054_SR2.IMG

H9700_0055_SR2.IMG

H9700_0056_SR2.IMG

H9700_0057_SR2.IMG

H9700_0058_SR2.IMG

H9700_0059_SR2.IMG

H9700_0060_SR2.IMG

H9700_0061_SR2.IMG

H9700_0062_SR2.IMG

H9700_0063_SR2.IMG

H9700_0064_SR2.IMG

H9700_0065_SR2.IMG

H9700_0066_SR2.IMG

H9700_0067_SR2.IMG

H9700_0068_SR2.IMG

H9700_0069_SR2.IMG

H9700_0070_SR2.IMG

H9700_0071_SR2.IMG

H9700_0072_SR2.IMG

H9700_0073_SR2.IMG

H9700_0074_SR2.IMG

H9700_0075_SR2.IMG

H9700_0076_SR2.IMG

H9700_0077_SR2.IMG

H9700_0078_SR2.IMG

H9700_0079_SR2.IMG

H9700_0080_SR2.IMG

H9700_0081_SR2.IMG

H9700_0082_SR2.IMG

H9700_0083_SR2.IMG

H9700_0084_SR2.IMG

H9700_0085_SR2.IMG

H9700_0086_SR2.IMG

H9700_0087_SR2.IMG

H9700_0088_SR2.IMG

H9700_0089_SR2.IMG

H9700_0090_SR2.IMG

H9700_0091_SR2.IMG

H9700_0092_SR2.IMG

H9700_0093_SR2.IMG

H9700_0094_SR2.IMG

H9700_0095_SR2.IMG

H9700_0096_SR2.IMG

H9700_0097_SR2.IMG

H9700_0098_SR2.IMG

H9700_0099_SR2.IMG

H9700_0100_SR2.IMG

H9700_0101_SR2.IMG

H9700_0102_SR2.IMG

H9700_0103_SR2.IMG

H9700_0104_SR2.IMG

H9700_0105_SR2.IMG

H9700_0106_SR2.IMG

H9700_0107_SR2.IMG

H9700_0108_SR2.IMG

H9700_0109_SR2.IMG

H9700_0110_SR2.IMG

H9700_0111_SR2.IMG

H9700_0112_SR2.IMG

H9700_0113_SR2.IMG

H9700_0114_SR2.IMG

H9700_0115_SR2.IMG

H9700_0116_SR2.IMG

H9700_0117_SR2.IMG

H9700_0118_SR2.IMG

H9700_0119_SR2.IMG

H9700_0120_SR2.IMG

H9700_0121_SR2.IMG

H9700_0122_SR2.IMG

H9700_0123_SR2.IMG

H9700_0124_SR2.IMG

H9700_0125_SR2.IMG

H9700_0126_SR2.IMG

H9700_0127_SR2.IMG

H9700_0128_SR2.IMG

H9700_0129_SR2.IMG

H9700_0130_SR2.IMG

H9700_0131_SR2.IMG

HC144_0004_SR2.IMG

HC144_0005_SR2.IMG

HC144_0006_SR2.IMG

HC222_0003_SR2.IMG

HC235_0003_SR2.IMG

HC326_0003_SR2.IMG

HC552_0007_SR2.IMG

HC921_0015_SR2.IMG

HC921_0016_SR2.IMG

HC921_0017_SR2.IMG

HC921_0018_SR2.IMG

HC921_0019_SR2.IMG

HC921_0020_SR2.IMG

HC921_0021_SR2.IMG

HC921_0022_SR2.IMG

HC921_0023_SR2.IMG

HC921_0024_SR2.IMG

HC921_0025_SR2.IMG

HC921_0026_SR2.IMG

HC921_0027_SR2.IMG

HC921_0028_SR2.IMG

HC921_0029_SR2.IMG

HC921_0030_SR2.IMG

HC921_0032_SR2.IMG

HC921_0033_SR2.IMG

HC921_0034_SR2.IMG

HC921_0035_SR2.IMG

HC921_0036_SR2.IMG

HC921_0037_SR2.IMG

HC921_0038_SR2.IMG

HC921_0039_SR2.IMG

HC921_0040_SR2.IMG

HC921_0041_SR2.IMG

HC921_0042_SR2.IMG

HC921_0043_SR2.IMG

HC921_0044_SR2.IMG

HC921_0045_SR2.IMG

HC921_0046_SR2.IMG

HC921_0047_SR2.IMG

HC921_0048_SR2.IMG

HC921_0049_SR2.IMG

HC921_0050_SR2.IMG

HC921_0051_SR2.IMG

HC921_0052_SR2.IMG

HC921_0053_SR2.IMG

HC921_0054_SR2.IMG

HC921_0055_SR2.IMG

HC921_0056_SR2.IMG

HC921_0057_SR2.IMG

HC921_0058_SR2.IMG

HC921_0059_SR2.IMG

HC921_0060_SR2.IMG

HC921_0061_SR2.IMG

HC921_0062_SR2.IMG

HC921_0063_SR2.IMG

HC921_0064_SR2.IMG

HC921_0065_SR2.IMG

HC921_0066_SR2.IMG

HC921_0067_SR2.IMG

HC921_0068_SR2.IMG

HC921_0069_SR2.IMG

HC921_0070_SR2.IMG

HC921_0071_SR2.IMG

HC921_0072_SR2.IMG

HC921_0073_SR2.IMG

HD469_0002_SR2.IMG

HD469_0007_SR2.IMG

HD647_0003_SR2.IMG

HD742_0003_SR2.IMG

HE084_0005_SR2.IMG

HE084_0006_SR2.IMG

HE084_0007_SR2.IMG

HE084_0008_SR2.IMG

HE084_0009_SR2.IMG

HE084_0010_SR2.IMG

HE084_0011_SR2.IMG

HE084_0012_SR2.IMG

HE084_0013_SR2.IMG

HE084_0014_SR2.IMG

HE084_0015_SR2.IMG

HE084_0016_SR2.IMG

HE084_0017_SR2.IMG

HE084_0018_SR2.IMG

HE084_0019_SR2.IMG

HE084_0020_SR2.IMG

HE084_0021_SR2.IMG

HE084_0022_SR2.IMG

HE084_0023_SR2.IMG

HE084_0024_SR2.IMG

HE084_0025_SR2.IMG

HE084_0026_SR2.IMG

HE084_0027_SR2.IMG

HE084_0028_SR2.IMG

HE084_0029_SR2.IMG

HE084_0030_SR2.IMG

HE084_0031_SR2.IMG

HE084_0032_SR2.IMG

HE084_0033_SR2.IMG

HE084_0034_SR2.IMG

HE084_0035_SR2.IMG

HE084_0036_SR2.IMG

HE084_0037_SR2.IMG

HE084_0038_SR2.IMG

HE084_0039_SR2.IMG

HE084_0040_SR2.IMG

HE084_0041_SR2.IMG

HE084_0042_SR2.IMG

HE084_0043_SR2.IMG

HE084_0044_SR2.IMG

HE084_0045_SR2.IMG

HE084_0046_SR2.IMG

HE084_0047_SR2.IMG

HE084_0048_SR2.IMG

HE084_0049_SR2.IMG

HE084_0050_SR2.IMG

HE084_0051_SR2.IMG

HE084_0052_SR2.IMG

HE084_0053_SR2.IMG

HE084_0054_SR2.IMG

HE084_0055_SR2.IMG

HE084_0056_SR2.IMG

HE084_0057_SR2.IMG

HE084_0058_SR2.IMG

HE084_0064_SR2.IMG

HE084_0065_SR2.IMG

HE084_0066_SR2.IMG

HE084_0067_SR2.IMG

HE084_0068_SR2.IMG

HE084_0069_SR2.IMG

HE084_0070_SR2.IMG

HE084_0071_SR2.IMG

HE084_0072_SR2.IMG

HE084_0073_SR2.IMG

HE084_0074_SR2.IMG

HE084_0075_SR2.IMG

HE084_0076_SR2.IMG

HE084_0077_SR2.IMG

HE084_0078_SR2.IMG

HE084_0079_SR2.IMG

HE084_0080_SR2.IMG

HE084_0081_SR2.IMG

HE084_0082_SR2.IMG

HE084_0083_SR2.IMG

HE084_0084_SR2.IMG

HE084_0085_SR2.IMG

HE084_0086_SR2.IMG

HE084_0087_SR2.IMG

HE084_0088_SR2.IMG

HE084_0089_SR2.IMG

HE084_0090_SR2.IMG

HE084_0091_SR2.IMG

HE084_0092_SR2.IMG

HE084_0093_SR2.IMG

HE084_0094_SR2.IMG

HE084_0095_SR2.IMG

HE084_0096_SR2.IMG

HE084_0097_SR2.IMG

HE084_0098_SR2.IMG

HE084_0099_SR2.IMG

HE084_0100_SR2.IMG

HE084_0101_SR2.IMG

HE084_0102_SR2.IMG

HE084_0103_SR2.IMG

HE084_0104_SR2.IMG

HE084_0105_SR2.IMG

HE084_0106_SR2.IMG

HE084_0107_SR2.IMG

HE084_0108_SR2.IMG

HE084_0109_SR2.IMG

HE084_0110_SR2.IMG

HE084_0111_SR2.IMG

HE084_0112_SR2.IMG

HE084_0113_SR2.IMG

HE084_0114_SR2.IMG

HE084_0115_SR2.IMG

HE084_0116_SR2.IMG

HE084_0117_SR2.IMG

HE433_0003_SR2.IMG

HE454_0007_SR2.IMG

HE454_0008_SR2.IMG

HE454_0009_SR2.IMG

HE454_0010_SR2.IMG

HE454_0011_SR2.IMG

HE454_0012_SR2.IMG

HE454_0013_SR2.IMG

HE454_0014_SR2.IMG

HE454_0015_SR2.IMG

HE454_0016_SR2.IMG

HE454_0017_SR2.IMG

HE454_0018_SR2.IMG

HE454_0019_SR2.IMG

HE454_0020_SR2.IMG

HE454_0021_SR2.IMG

HE454_0022_SR2.IMG

HE454_0023_SR2.IMG

HE454_0024_SR2.IMG

HE454_0025_SR2.IMG

HE454_0026_SR2.IMG

HE454_0027_SR2.IMG

HE454_0028_SR2.IMG

HE454_0029_SR2.IMG

HE454_0030_SR2.IMG

HE454_0031_SR2.IMG

HE454_0032_SR2.IMG

HE454_0033_SR2.IMG

HE454_0034_SR2.IMG

HE454_0035_SR2.IMG

HE454_0036_SR2.IMG

HE454_0037_SR2.IMG

HE454_0038_SR2.IMG

HE454_0039_SR2.IMG

HE454_0040_SR2.IMG

HE454_0041_SR2.IMG

HE454_0042_SR2.IMG

HE454_0043_SR2.IMG

HE454_0044_SR2.IMG

HE454_0045_SR2.IMG

HE454_0046_SR2.IMG

HE454_0047_SR2.IMG

HE454_0048_SR2.IMG

HE454_0049_SR2.IMG

HE454_0050_SR2.IMG

HE454_0051_SR2.IMG

HE454_0052_SR2.IMG

HE454_0053_SR2.IMG

HE454_0054_SR2.IMG

HE454_0055_SR2.IMG

HE454_0056_SR2.IMG

HE454_0057_SR2.IMG

HE454_0058_SR2.IMG

HE454_0059_SR2.IMG

HE454_0060_SR2.IMG

HE454_0061_SR2.IMG

HE454_0062_SR2.IMG

HE454_0063_SR2.IMG

HE454_0064_SR2.IMG

HE454_0065_SR2.IMG

HE454_0066_SR2.IMG

HE454_0067_SR2.IMG

HE454_0068_SR2.IMG

HE454_0069_SR2.IMG

HE454_0070_SR2.IMG

HE454_0071_SR2.IMG

HE454_0072_SR2.IMG

HE454_0073_SR2.IMG

HE454_0074_SR2.IMG

HE454_0075_SR2.IMG

HE454_0076_SR2.IMG

HE454_0077_SR2.IMG

HE454_0078_SR2.IMG

HE454_0079_SR2.IMG

HE454_0080_SR2.IMG

HE454_0081_SR2.IMG

HE454_0082_SR2.IMG

HE454_0083_SR2.IMG

HE454_0084_SR2.IMG

HE454_0085_SR2.IMG

HE454_0086_SR2.IMG

HE454_0087_SR2.IMG

HE454_0088_SR2.IMG

HE454_0089_SR2.IMG

HE454_0090_SR2.IMG

HE454_0091_SR2.IMG

HE454_0092_SR2.IMG

HE454_0093_SR2.IMG

HF396_0003_SR2.IMG

HF527_0003_SR2.IMG

HF566_0004_SR2.IMG

HF566_0005_SR2.IMG

HF566_0006_SR2.IMG

HF596_0003_SR2.IMG

HF812_0019_SR2.IMG

HF812_0020_SR2.IMG

HF812_0021_SR2.IMG

HF812_0023_SR2.IMG

HF812_0024_SR2.IMG

HF812_0025_SR2.IMG

HF812_0026_SR2.IMG

HF812_0027_SR2.IMG

HF812_0028_SR2.IMG

HF812_0029_SR2.IMG

HF812_0031_SR2.IMG

HF812_0032_SR2.IMG

HF812_0033_SR2.IMG

HF812_0034_SR2.IMG

HF812_0035_SR2.IMG

HF812_0036_SR2.IMG

HF812_0037_SR2.IMG

HF812_0038_SR2.IMG

HF812_0039_SR2.IMG

HF812_0040_SR2.IMG

HF812_0041_SR2.IMG

HF812_0042_SR2.IMG

HF812_0043_SR2.IMG

HF812_0044_SR2.IMG

HF812_0045_SR2.IMG

HF812_0046_SR2.IMG

HF812_0047_SR2.IMG

HF812_0048_SR2.IMG

HF812_0049_SR2.IMG

HF812_0050_SR2.IMG

HF812_0051_SR2.IMG

HF812_0052_SR2.IMG

HF812_0053_SR2.IMG

HF812_0054_SR2.IMG

HF812_0055_SR2.IMG

HF812_0056_SR2.IMG

HF812_0057_SR2.IMG

HF812_0058_SR2.IMG

HF812_0059_SR2.IMG

HF812_0060_SR2.IMG

HF812_0061_SR2.IMG

HF812_0062_SR2.IMG

HF812_0063_SR2.IMG

HF812_0064_SR2.IMG

HF812_0065_SR2.IMG

HF812_0066_SR2.IMG

HF812_0067_SR2.IMG

HF812_0068_SR2.IMG

HF812_0069_SR2.IMG

HF812_0070_SR2.IMG

HF812_0071_SR2.IMG

HF812_0072_SR2.IMG

HF812_0073_SR2.IMG

HF812_0074_SR2.IMG

HF812_0075_SR2.IMG

HF812_0076_SR2.IMG

HF898_0001_SR2.IMG

HF898_0002_SR2.IMG

HF898_0003_SR2.IMG

HF898_0004_SR2.IMG

HF898_0005_SR2.IMG

HF898_0006_SR2.IMG

HF898_0007_SR2.IMG

HF898_0008_SR2.IMG

HF898_0009_SR2.IMG

HF898_0010_SR2.IMG

HF898_0011_SR2.IMG

HF898_0012_SR2.IMG

HF898_0013_SR2.IMG

HF898_0014_SR2.IMG

HF898_0015_SR2.IMG

HF898_0016_SR2.IMG

HF898_0017_SR2.IMG

HF898_0018_SR2.IMG

HF898_0019_SR2.IMG

HF898_0020_SR2.IMG

HF898_0021_SR2.IMG

HF898_0022_SR2.IMG

HF898_0023_SR2.IMG

HF898_0024_SR2.IMG

HF898_0025_SR2.IMG

HF898_0026_SR2.IMG

HF898_0027_SR2.IMG

HF898_0028_SR2.IMG

HF898_0029_SR2.IMG

HF898_0030_SR2.IMG

HF898_0031_SR2.IMG

HF898_0032_SR2.IMG

HF898_0033_SR2.IMG

HF898_0034_SR2.IMG

HF898_0035_SR2.IMG

HF898_0036_SR2.IMG

HF898_0037_SR2.IMG

HF898_0038_SR2.IMG

HF898_0039_SR2.IMG

HF898_0040_SR2.IMG

HF898_0041_SR2.IMG

HF898_0042_SR2.IMG

HF898_0043_SR2.IMG

HF898_0044_SR2.IMG

HF898_0045_SR2.IMG

HF898_0046_SR2.IMG

HF898_0047_SR2.IMG

HF898_0048_SR2.IMG

HF898_0049_SR2.IMG

HF898_0050_SR2.IMG

HF898_0051_SR2.IMG

HF898_0052_SR2.IMG

HF898_0053_SR2.IMG

HF898_0054_SR2.IMG

HF898_0055_SR2.IMG

HF898_0056_SR2.IMG

HF898_0057_SR2.IMG

HF898_0058_SR2.IMG

HF898_0059_SR2.IMG

HF898_0060_SR2.IMG

HF898_0061_SR2.IMG

HF898_0062_SR2.IMG

HF898_0063_SR2.IMG

HF898_0064_SR2.IMG

HF898_0065_SR2.IMG

HF898_0066_SR2.IMG

HF898_0067_SR2.IMG

HF898_0068_SR2.IMG

HF898_0069_SR2.IMG

HF898_0070_SR2.IMG

HF898_0071_SR2.IMG

HF898_0072_SR2.IMG

HF898_0073_SR2.IMG

HF898_0074_SR2.IMG

HF898_0075_SR2.IMG

HF898_0076_SR2.IMG

**# HiRISE**

None. All HiRISE images were used to construct the model.
